# High-depth sequencing of over 750 genes supports linear progression of primary tumors and metastases in most patients with liver-limited metastatic colorectal cancer

**DOI:** 10.1186/s13059-015-0589-1

**Published:** 2015-02-12

**Authors:** Iain Beehuat Tan, Simeen Malik, Kalpana Ramnarayanan, John R McPherson, Dan Liang Ho, Yuka Suzuki, Sarah Boonhsui Ng, Su Yan, Kiat Hon Lim, Dennis Koh, Chew Min Hoe, Chung Yip Chan, Rachel Ten, Brian KP Goh, Alexander YF Chung, Joanna Tan, Cheryl Xueli Chan, Su Ting Tay, Lezhava Alexander, Niranjan Nagarajan, Axel M Hillmer, Choon Leong Tang, Clarinda Chua, Bin Tean Teh, Steve Rozen, Patrick Tan

**Affiliations:** Department of Medical Oncology, National Cancer Centre Singapore, 11 Hospital Drive, Singapore, 169610 Singapore; Cancer & Stem Cell Biology Program, Duke-National University of Singapore Graduate Medical School, Singapore, 169857 Republic of Singapore; Genome Institute of Singapore, Singapore, 138672 Singapore; National University of Singapore Graduate School of Integrative Sciences and Engineering, National University of Singapore, Singapore, 119077 Singapore; Cancer Science Institute Singapore, National University of Singapore, Singapore, 117599 Singapore; Division of Cellular and Molecular Research, National Cancer Centre Singapore, Singapore, 169610 Singapore; Institute of Cellular and Molecular Biology, Singapore, 138673 Singapore; Department of Pathology, Singapore General Hospital, Singapore, 169856 Singapore; Colorectal Surgery, Singapore General Hospital, Singapore, 169856 Singapore; General Surgery, Singapore General Hospital, Singapore, 169856 Singapore; Laboratory of Cancer Epigenome, National Cancer Centre Singapore, 11 Hospital Drive, Singapore, 169610 Singapore

## Abstract

**Background:**

Colorectal cancer with metastases limited to the liver (liver-limited mCRC) is a distinct clinical subset characterized by possible cure with surgery. We performed high-depth sequencing of over 750 cancer-associated genes and copy number profiling in matched primary, metastasis and normal tissues to characterize genomic progression in 18 patients with liver-limited mCRC.

**Results:**

High depth Illumina sequencing and use of three different variant callers enable comprehensive and accurate identification of somatic variants down to 2.5% variant allele frequency. We identify a median of 11 somatic single nucleotide variants (SNVs) per tumor. Across patients, a median of 79.3% of somatic SNVs present in the primary are present in the metastasis and 81.7% of all alterations present in the metastasis are present in the primary. Private alterations are found at lower allele frequencies; a different mutational signature characterized shared and private variants, suggesting distinct mutational processes. Using B-allele frequencies of heterozygous germline SNPs and copy number profiling, we find that broad regions of allelic imbalance and focal copy number changes, respectively, are generally shared between the primary tumor and metastasis.

**Conclusions:**

Our analyses point to high genomic concordance of primary tumor and metastasis, with a thick common trunk and smaller genomic branches in general support of the linear progression model in most patients with liver-limited mCRC. More extensive studies are warranted to further characterize genomic progression in this important clinical population.

**Electronic supplementary material:**

The online version of this article (doi:10.1186/s13059-015-0589-1) contains supplementary material, which is available to authorized users.

## Background

Colorectal cancer (CRC), the third most common cancer worldwide [1], accounts for about 10% of the global cancer burden. Colorectal cancer with metastases limited to the liver (liver-limited mCRC) is characterized by a unique treatment paradigm [2]. For most cancers, the presence of liver metastases signals disseminated cancer with a consequent palliative treatment intent. However, in a subset of patients with liver-limited mCRC, surgical resection of all visible disease, often accompanied by peri-operative chemotherapy, can lead to long-term disease control, remission and possible cure [3-5]. This striking natural history, which has motivated aggressive treatment strategies in the clinic, also raises questions about the evolving genetic determinants contributing to disease progression in this distinct subset of patients with liver-limited mCRC.

Comparative genomic studies examining the degree of genetic divergence between matched primary tumors and metastases from individual patients provide insights into the genetic events involved in tumor initiation and progression. Several models of cancer progression have been proposed [6], including linear progression [7] where genetic alterations accumulate in the primary in a step-wise manner leading to acquisition of metastatic traits. In the linear progression model, successful metastasis is an end-stage event requiring acquisition of a full complement of traits resulting in advanced clones carrying a large complement of accumulated alterations found in both the primary and the metastasis. In the parallel progression [8] model, tumor cells disseminate from the primary tumor very early in its development and may be subsequently genetically modified in the metastatic niche where they later settle. The parallel progression model predicts that disseminated tumor cells would be genetically divergent from cells found in the primary tumor as metastatic cells and primary cells would adapt to their separate environments in parallel and acquire distinct genetic alterations. Across different tumor types [9-14], recent comparative genomic studies report varying degrees of divergence of genomic profiles of primary tumors and matched metastases. Given that liver-limited metastatic colorectal cancer represents a unique clinical phenotype where the natural history of the disease makes long-term disease control and cure possible, we performed a comparative genomic study to understand the genomic determinants of cancer progression in this distinct clinical subset.

In this paper, we performed targeted next generation sequencing (NGS) on the primary colorectal tumor, liver metastasis and normal colonic tissue representing 54 tissue specimens from 18 patients with liver-limited mCRC. Our strategy first focuses on evaluating a set of 750 cancer-associated genes comprising genes biologically and clinically relevant to cancer, such as genes involved in key oncogenic signaling pathways, oncogenes, tumor suppressor genes and genes from kinase and chromatin remodeler families, which we curated from literature review and existing databases (for example, COSMIC and cancer gene census). Alterations in cancer-associated genes likely have biological significance and acquisition of these alterations is important for clonal progression of cancer. High depth sequencing of these cancer-associated genes is necessary to allow evaluation of subclonal architecture of primary and metastatic tumors as cancer progresses. We thus sequenced, with an Illimuina NGS platform, a panel of over 750 known cancer-associated genes to high depths of over 350× (several-fold higher that what is typically achieved with whole genome or exome sequencing) and employed three different bioinformatics algorithms to identify somatic alterations, including those present at low frequency. Only 39% of the variants identified were called by all three algorithms and 23% of variants were called by only one algorithm. Importantly, we validated 95% of variants identified with an orthogonal NGS platform, Ion torrent, demonstrating that one may need to perform several bioinformatics algorithms to take full advantage of high-depth sequencing data to comprehensively identify somatic variants and elucidate clonal architecture in comparative genomics studies. Using this approach, we found high concordance of genomic alterations in both the primary tumor and liver metastasis across over 750 cancer-associated genes in liver-limited mCRC. Across our patients, a median of 79.3% of alterations present in the primary tumor were present in the metastasis (range 18 to 100%) and 81.7% of all alterations present in the metastasis were already present in the primary (range 50 to 100%). Shared variants were characterized by high allele frequency whilst variants unique to the primary or metastasis were generally of low allele frequency. Next, taking advantage of the relatively large size of our targeted sequencing panel, we were able to identify, from sequencing of each patient’s non-neoplastic tissue, a sizeable number (median of 1,949 per patient) of heterozygous germline SNPs. In the corresponding primary tumor or metastatic tissue, the B-allele frequencies of these SNPs would deviate substantially from 0.5 in regions of allelic imbalance. When mapped onto the relevant chromosomal locations, broad regions of allelic imbalance were found to be generally similar in the primary tumor and in the liver metastasis. In some patients, however, there were additional small regions of allelic imbalance private to either the primary or the metastasis. Additionally, we performed copy number analysis of 87 genes commonly amplified or deleted in cancer using the Nanostring nCounter v2 Cancer Copy Number Assay on DNA extracted from the matched normal, primary tumor and metastatic tumor from our 20 patients. We found that focal copy number alterations are also generally shared between the primary tumor and metastasis. Taken together, our findings support a linear progression model in CRC with branched evolution characterized by a common thick trunk before branching off a smaller set of alterations unique to the primary or the metastasis.

Although single nucleotide variants (SNVs), broad regions of allelic imbalance and focal copy number alterations were generally similar in the primary and metastasis, supporting linear progression in the majority of our patients with liver-limited mCRC, we found evidence of a different mutational signature/context amongst ‘shared’ truncal variants present in both the primary and metastasis and ‘private’ branch alterations unique to the primary or the metastasis. This suggests that beyond linear progression in the primary and metastasis, there may be distinct mutational processes involved in different phases of cancer progression.

## Results

### Customized enrichment and targeted NGS achieves high coverage across 750 cancer-associated genes

We used the Agilent SureSelect Target Enrichment system to design a customized enrichment panel comprising all coding exons of over 750 cancer-associated genes. From Genomic DNA extracted from 24 frozen tissue specimens (matched primary tumor, adjacent normal and liver metastasis) from an initial 8 patients with liver-limited mCRC (Table 1), we performed library construction, target enrichment and Illimuina NGS. A median of 504-fold average base coverage (range 222 to 751×) was obtained across the target exons (Additional file 1). In all samples, at least 96% of target bases were spanned by at least 30 reads. Across samples, a median of 86.4% of target bases were spanned by at least 200 sequence reads.

**Table 1:** **Patient characteristics**

**No.**	**Temporal relationship of metastasis to primary tumor**	**MSI status**	**KRAS/BRAF mutational status**	**Stage at diagnosis**	**Month of surgery for primary tumor**	**Adjuvant therapy**	**Disease-free interval**	**Date of diagnosis of recurrence**	**Chemotherapy for metastasis**	**Objective response to chemotherapy**	**Month of liver resection**
1	Synchronous	MSS	Wild type for both	4	Jun 2012				3 cycles of XELOX	Partial response	Nov 2012
2	Synchronous	MSS	Wild type for both	4	Oct 2012						Oct 2012
3	Synchronous	MSS	Wild type for both	4	Jan 2011						Jan 2011
4	Synchronous	MSS	Wild type for both	4	May 2011				5 cycles of XELOX	Partial response	Oct 2011
5	Metachronous	MSS	Wild type for both	3	Nov 2010	XELOX	22 months	Aug 2012			Aug 2012
6	Synchronous	MSS	Wild type for both	4	Nov 2012						Nov 2012
7	Metachronous	MSS	Wild type for both	3	Nov 2011	Xeloda/RT and XELOX	8 months	Jul 2012	3 cycles of XELIRI	Stable disease	Nov 2012
8	Synchronous	MSS	Wild type for both	4	Jul 2013						Jul 2013
9	Synchronous	MSS	Wild type for KRAS	4	Jul 2011				6 cycles of XELOX and cetuximab	Stable disease	Jan 2012
10	Synchronous	MSS	Wild type for both	4	Apr 2013				8 cycles of XELOX	Stable disease	Apr 2014
11	Synchronous	MSS	Wild type for both	4	Mar 2014				6 cycles of FOLFOX with cetuximab from cycle 3 onwards	Stable disease	Apr 2014
12	Synchronous	MSS	Wild type for both	4	Aug 2013						Dec 2013
13	Metachronous	MSS	Wild type for both	2	Jun 2012	None	19 months	Jan 2014			Feb 2014
14	Synchronous	MSS	KRAS p.G12V	4	May 2014						May 2014
15	Synchronous	MSS	KRAS p.G12V	4	May 2014				8 cycles of XELOX	Partial response	May 2014
16	Metachronous	MLH-1 and PMS-2 loss	Wild type for KRAS	2	Aug 2004	None	78 months	Feb 2011			May 2012
17	Synchronous	MSS	Wild type for both	4	Mar 2012				2 cycles of XELOX and cetuximab	Partial response	May 2012
18	Synchronous	MSS	Wild type for both	4	Mar 2013						Mar 2013

Clinico-pathologic and treatment details for the 18 patients. MSI: microsattelite instability; MSS: microsattelite stable.

### Use of three different bioinformatics algorithms enables sensitive and comprehensive identification of somatic variants, including those present at low allele frequency

We used three different bioinformatics algorithms to identify somatically acquired non-synonymous SNVs in the eight primary tumors and eight metastases, using the matched normal mucosa as the reference. The three algorithms, detailed below in the Materials and methods section, are a Genome Analyzer Toolkit (GATK)-based pipeline [15], LoFreq [16] and MuTect [17]. In total, 186 variants were called by the 3 pipelines (Additional file 2); 72 variants (39%) were identified by all 3 algorithms; 43 variants (23%) were identified only by one of the algorithms.

To evaluate the reliability of variants that were called, we re-sequenced these variants using an orthogonal library construction, enrichment and sequencing strategy. We designed custom primers targeting these variants with the Ampliseq primer design software. We were able to design customized primers for 180 of the 186 variants. Samples were prepared for targeted sequencing using the Ion Torrent. One variant had very poor coverage and was thus not interpretable. Sequence reads provided sufficient coverage for 179 of the 180 variants. Of the 179 variants assessed, 170 were validated by orthogonal sequencing (Ion Torrent). The true positive rate was 95%. We could evaluate 35 variants that had been called to be present only in either the primary tumor but not the matched metastasis (17 variants) or the metastasis but not the matched primary tumor (18 variants). Of these 35 variants, the absence of the variant in the corresponding tissue was confirmed in 34 cases. Using this metric, the true negative rate was estimated to be 97%.

GATK, Lofreq and MuTect identified 136, 156 and 109 variants, respectively. The median variant allele frequency (VAF) of somatic variants identified by the algorithms was 35% (range 6 to 90%), 28% (2 to 90%) and 24% (3 to 90%), respectively. Amongst variants on which we performed orthogonal validation, the true positive rate was 100% (136/136) for GATK, 98.7% (149/151) for LoFreq and 92.3% (96/104) for MuTect. The distribution, true positive rate and mean allele frequency of variants identified by the various algorithms are summarized as a Venn diagram in Additional file 2. These results show that use of specific bioinformatics algorithms identifies additional low allele frequency variants that would otherwise be missed if only one algorithm was used. Of note, 15, 14 and 4 validated somatic variants were identified by GATK alone, Lofreq alone or MuTect alone, respectively. In particular, Lofreq and Mutect were able to identify somatic variants that were present at low allele frequency that were missed by GATK. All variants identified by the GATK pipeline were true positives. The median allele frequency of variants identified by GATK was 35% (range 6 to 90%). In contrast, the median allele frequency of variants missed by GATK was 7% (range: 2 to 54%). The true positive rate for somatic variants missed by GATK and called by either or both Lofreq and MuTect was 82% (41/50). Nine variants could not be validated. These false positive variants were called at low variant allele frequencies (median 7%; range 3 to 13%), which overlapped with the allele frequencies of some of the true positives.

The true positive rate of our combined approach was 95%. A total of 170 variants were validated; the sensitivity of each algorithm alone was 75.9% for GATK (129/170), 87.6% for LoFreq (149/170) and 56.4% for Mutect (96/170).

Use of all three algorithms allowed comprehensive identification of somatic variants in our patients. Taken together, these validation results provide confidence that our approach accurately identifies the presence or absence of somatic alterations in tumor samples, including variants present at low allele frequency.

### High depth NGS and variant calling using three algorithms performed on additional patients with liver-limited mCRC

Having validated our approach for achieving very high true positive and true negative rates, we applied the same approach on genomic DNA extracted from an additional 30 frozen tissue specimens (matched primary tumor, adjacent normal and liver metastasis) from 10 patients with liver-limited mCRC (Table 1). Across all 18 patients, a median of 385-fold average base coverage (range 131 to 751×) was obtained across the target exons (Additional file 1). In all samples, at least 92% of target bases were spanned by at least 30 reads. Across samples, a median of 77.7% of target bases were spanned by at least 200 sequence reads.

GATK, MuTect and LoFreq identified 853, 1494 and 1916 variants, respectively. The median VAF of somatic variants identified by the algorithms was 20.6% (range 6 to 95%), 15.6% (2 to 93%) and 15.8% (3 to 94%), respectively. We examined the median tumor and normal allele frequencies and median depth for mutations that were identified by only one of the three callers. The median VAF for the GATK-based algorithm is higher than those for LoFreq and MuTect. The median total depth of the variants called only by GATK is also lower than those called by LoFreq and MuTect (Additional file 3). Although methodological analysis of factors affecting the sensitivity of various algorithms is outside the scope of this current paper on genomic diversity of primary and metastatic CRC tumors, these data suggest that high coverage allows for high sensitivity in the tumor but may lead to a higher rate of false positive calls in the normal, leading to a potential loss in sensitivity which can be overcome by using three different variant calling algorithms each with different parameter settings and heuristic thresholds intrinsic to each method.

### High degree of similarity amongst variants present in matched primary tumor and liver metastases

**Figure 1:**
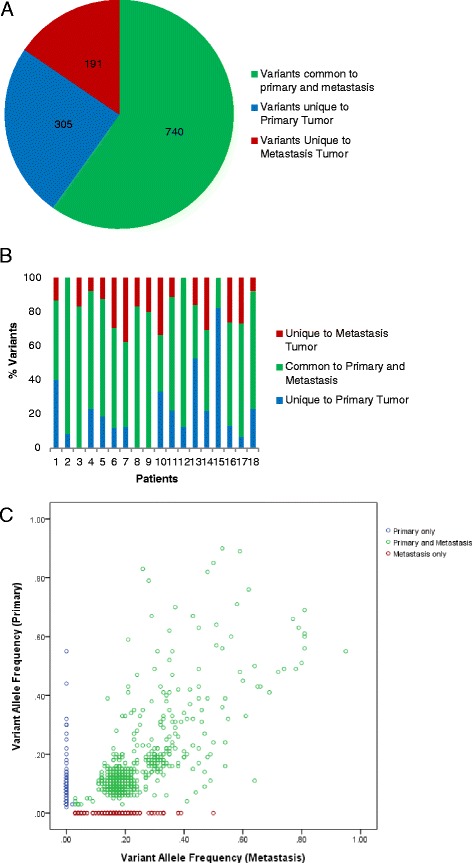
**Distribution of variants across the 18 patients. (A)** Pie chart and **(B)** stacked bar chart showing the proportion of variants that are shared (green), private to primary (blue) and private to metastasis (red). **(C)** Scatter plot of all variants found in the 18 patients. The y-axis indicates allele frequency of variants present in the primary; the x-axis indicates allele frequency of variants present in the matched liver metastasis.

**Figure 2:**
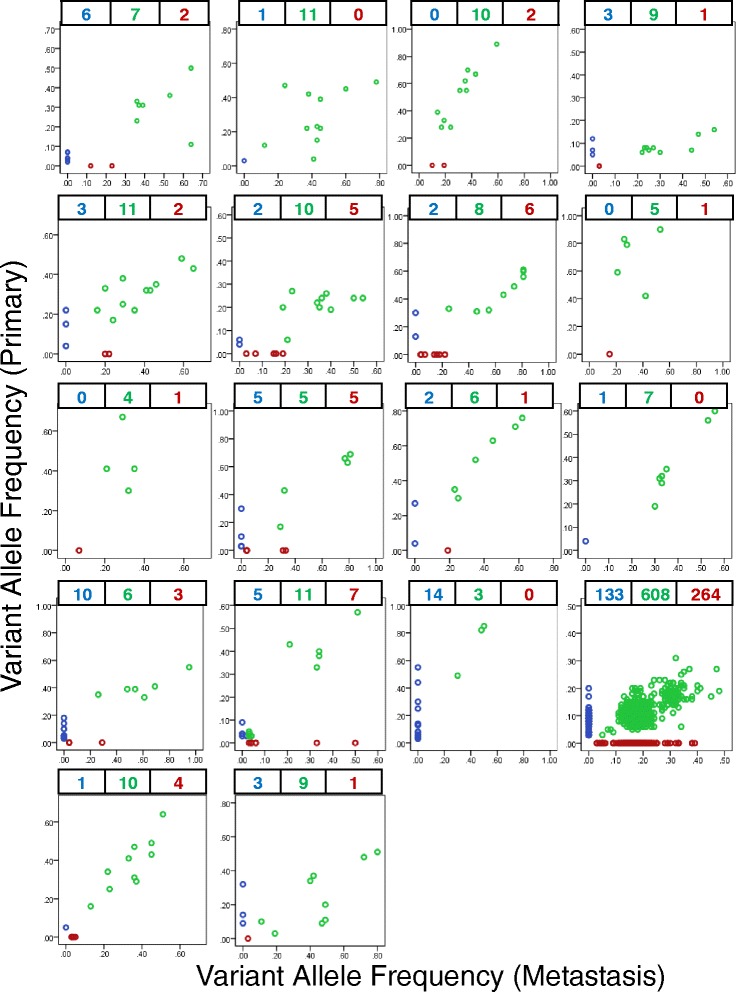
**Distribution of variants in each patient.** Scatter plots of variants found in each patient. The y-axis indicates allele frequency of variants present in the primary; the x-axis indicates allele frequency of variants present in the matched liver metastasis.

After accounting for the nine false positives and one false negative, 1,976 non-synonymous SNVs were identified amongst 36 tissue specimens comprising 18 primary and 18 metastatic tumors (median 11 per tumor, range 3 to 872). One patient was an ultra-mutant (POLE mutation) with 741 variants in the primary and 872 variants in the metastasis. Across the 18 patients, there were 1,236 distinct variants (Additional file 4; the ultra-mutant patient had 1,005 distinct variants) and a median of 79.3% of somatic SNVs present in the primary tumor were present in the metastasis (range 18 to 100%) and 81.7% of somatic SNVs present in the metastasis were already present in the primary (range 50 to 100%) (Table 2 and Figure 1). There were 740 variants shared in matched primary and metastasis pairs (median 8.5 per tumor; range 3 to 608) (Figure 1), and 191 variants were found private to the primary and 305 variants were private to the metastasis. Our patients had a median of 15 distinct variants (range 5 to 1,005), with a median of 2.5 variants unique to the primary (range 0 to 133), a median of 2 variants unique to the metastasis (range 0 to 264) and a median of 8.5 variants in common (range 3 to 608) (Figure 2).

**Table 2:** **Unique and common variants found in the primary tissue and metastasis for each patient in the study**

**Patient**	**Synchronous or metachronous**	**Receipt of chemotherapy prior to surgery for liver metastasis**	**Variants common to primary and metastasis (number (mean VAF))**	**Variants unique to primary tumor (number (mean VAF))**	**Total variants in primary (number (mean VAF))**	**Percentage of variants in primary found in metastasis**	**Variants common to primary and metastasis (number (mean VAF))**	**Variants unique to metastasis tumor (number (mean VAF))**	**Total variants in metastasis**	**Percentage of variants in metastasis found in primary**	**Total distinct variants in each patient**
1	Synchronous	Yes	7 (31%)	6 (5%)	13 (19%)	53.8%	7 (47%)	2 (18%)	9 (40%)	77.8%	15
2	Synchronous	No	11 (29%)	1 (3%)	12 (27%)	91.7%	11 (42%)	0 (NA)	11 (42%)	100.0%	12
3	Synchronous	No	10 (53%)	0 (NA)	10 (53%)	100.0%	10 (32%)	2 (15%)	12 (29%)	83.3%	12
4	Synchronous	Yes	9 (9%)	3 (8%)	12 (9%)	75.0%	9 (33%)	1 (3%)	10 (30%)	90.0%	13
5	Metachronous	Yes	11 (32%)	3 (14%)	14 (28%)	78.6%	11 (37%)	2 (21%)	13 (35%)	84.6%	16
6	Synchronous	No	10 (21%)	2 (5%)	12 (19%)	83.3%	10 (35%)	5 (12%)	15 (27%)	62.5%	17
7	Metachronous	Yes	8 (46%)	2 (22%)	10 (41%)	80.0%	8 (64%)	6 (13%)	14 (42%)	53.3%	16
8	Synchronous	No	5 (71%)	0 (NA)	5 (71%)	100.0%	5 (34%)	1 (15%)	6 (31%)	83.3%	6
9	Synchronous	Yes	4 (45%)	0 (NA)	4 (45%)	100.0%	4 (29%)	1 (7%)	5 (25%)	80.00%	5
10	Synchronous	Yes	5 (51%)	5 (10%)	10 (31%)	50.00%	5 (60%)	5 (15%)	10 (37%)	50.00%	15
11	Synchronous	Yes	6 (54%)	2 (16%)	8 (45%)	75.00%	6 (41%)	1 (19%)	7 (38%)	85.71%	9
12	Synchronous	No	7 (37%)	1 (4%)	8 (33%)	87.50%	7 (39%)	0 (NA)	7 (39%)	100.00%	8
13	Metachronous	No	6 (40%)	10 (8%)	16 (20%)	37.50%	6 (58%)	3 (12%)	9 (43%)	66.67%	19
14	Synchronous	No	11 (21%)	5 (4%)	16 (16%)	68.75%	11 (18%)	7 (15%)	18 (17%)	61.11%	23
15	Synchronous	Yes	3 (72%)	14 (17%)	17 (26%)	17.65%	3 (42%)	0 (NA)	3 (42%)	100.00%	17
16	Metachronous	No	608 (12%)	133 (9%)	741 (11%)	82.1%	608 (20%)	264 (17%)	872 (19%)	69.7%	1005
17	Synchronous	Yes	10 (38%)	1 (5%)	11 (35%)	90.91%	10 (34%)	4 (4%)	14 (25%)	71.43%	15
18	Synchronous	No	9 (25%)	3 (18%)	12 (23%)	75.00%	9 (45%)	1 (3%)	10 (41%)	90.00%	13
Total			740 (38%)	191 (10%)	931 (30%)	79.4%^a^	740 (39%)	305 (13%)	1045 (34%)	70.8%^a^	1236

^a^Across the 18 patients, a median of 79.3% of variants in the primary are found in the metastasis, and a median of 81.7% of variants found in the metastasis are found in the primary.

### No major differences in genomic similarity amongst synchronous and metachronous tumors and between patients with and without prior chemotherapy

We did not observe a major difference in diversity between metachronous and synchronous patients nor between patients who received or did not receive prior systemic chemotherapy between the resection of the primary and the metastasis, either as adjuvant treatment after primary resection or ‘neo-adjuvant’ therapy prior to resection of metastasis. Four of the patients had metachronous metastasis. In these patients, 70% of variants in the primary were found in the metastasis and 69% of variants found in the metastasis were found in the primary. This tended to be lower than for patients with synchronous metastasis where 76% of mutations in the primary were found in the metastasis and 81% of variants in the metastasis were found in the primary. Nine of the patients had received prior chemotherapy and in these 69% of variants present in the primary were present in the metastasis and 77% of variants in the metastasis were present in the primary. Amongst the nine patients without prior chemotherapy, 81% of variants present in the primary were present in the metastasis and 80% of variants in the metastasis were present in the primary. These differences were not statistically significant.

### Characteristics of shared variants and private variants

We examined characteristics of variants more likely to be shared amongst primary tumor and metastasis (shared) or those likely to be present only in the primary or metastasis. Variants were reported in 487 genes; well described colorectal cancer driver mutations [18] were often shared. Six distinct KRAS non-synonymous variants were all found in both the primary and metastasis. There were 25 and 13 distinct variants in APC and p53, respectively, reported across the cohort. Of the 25 APC variants and 13 p53 variants, 21 and 11, respectively, were found in both the primary and the metastasis (Additional file 4). Similarly, amongst genes reported by The Cancer Genome Atlas (TCGA) to be significantly mutated in CRC, 85% of non-synonymous variants in the metastasis were already present in the primary (versus 70% for all other cancer-associated genes; *P* = 0.04).

In 283 genes, variants were identified in more than one patient (recurrent alterations amongst the patients; Additional file 5). Overall, variants in genes recurrently altered amongst the patients were more likely to be shared (*P* = 0.01); 81% (634 of 786) of recurrent mutations found in the primary were found in the metastasis, and 72% (634 of 880) of recurrent mutations found in the metastasis were already found in the primary.

Mutations found at higher allele frequency were also more likely to be present in the corresponding matched tumor. In the primary tumors, the mean VAF for variants solely in the primary is 10% versus 38% for variants ‘shared’ with the metastasis (*P* < 0.01). In the metastatic tumors, the mean allele frequency for variants solely in the metastasis is 13% versus 39% for variants ‘shared’ with the primary (*P* < 0.01).

The relative VAFs of variants solely in the primary and ‘shared’ with or common to the primary and metastasis supports a model where dominant clones from the primary gave rise to the metastasis and, within each tissue, further subclones found at lower frequencies began to develop.

We did observe that certain genes were enriched amongst variants present only in the metastasis. For example, variants in *MLL3*, *FAT1* and *GNAS* were often observed private to the metastasis. We next examined if genes associated with shared or private localities were associated with specific cancer pathways, using Ingenuity Pathway Analysis (IPA). Interestingly, IPA analysis showed notch signaling and tight junction signaling pathway to be altered only amongst private alterations. A list of pathways unique to variants that are common or unique to variants that are private is provided in Additional file 6.

### Distinct mutational signatures are observed in shared variants and private variants

**Figure 3:**
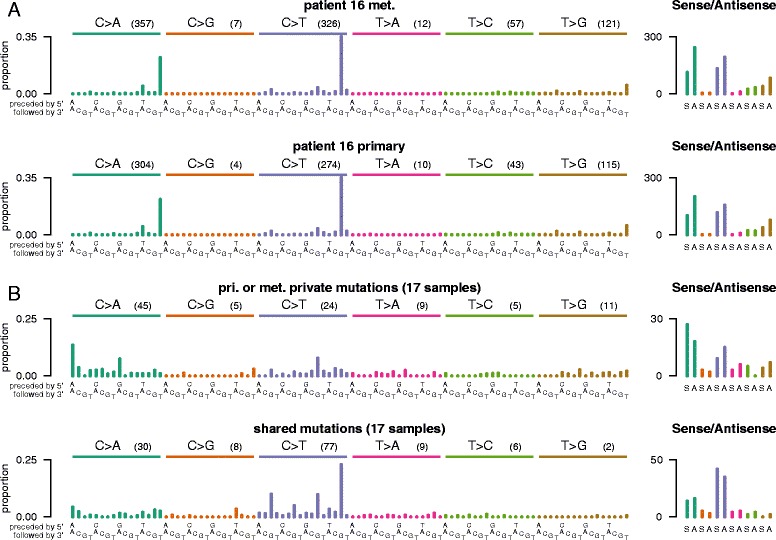
**Mutational signatures.** Trinucleotide and substitution mutational context of **(A)** primary and metastasis of patient 16 (ultra-mutant) and **(B)** mutations that are private to either the primary or metastasis and mutations that are common to the primary and metastasis in the remaining 17 patients.

We also examined if any mutational signatures were enriched amongst common mutations or mutations that are private to the primary or private to the metastasis. This analysis could provide insight into distinct mutational processes involved in cancer progression. One patient has somatic mutations in the *POLE* gene, which is involved in DNA replication and repair and mutated POLE is associated with an unusually high rate of mutations. The mutational process found in *POLE* ultra-mutants is associated with a mutational signature comprising TCT > TAT and TCG > TTG mutations. This mutational signature was observed in both the primary tumor and the metastasis of this patient as well as the shared variants found in both the primary and metastasis or private only to the primary or metastasis (Figure 3A). This suggests that the same dominant mutational process is involved in cancer progression in this patient with *POLE* mutation. In the remaining patients, known colorectal-associated mutagenic processes (microsatellite instability and *POLE* mutations) were absent. We examined the mutational contexts of variants shared in the primary and metastasis and variants private to the primary or the metastasis. Remarkably, we observed distinct mutational signatures amongst shared mutations and private mutations. Somatic mutations common to the primary and metastasis are mostly CpG > TpG mutations, which have been associated with deanimation of methylated cytosines and an ‘aging process’, while mutations private to either the primary or the metastasis were mostly C > A and other C > T mutations, suggesting that a distinct complement of mutational processes are active during different phases of cancer progression (Figure 3B).

### Regions of allelic imbalance are similar in the matched primary and metastasis

**Figure 4:**
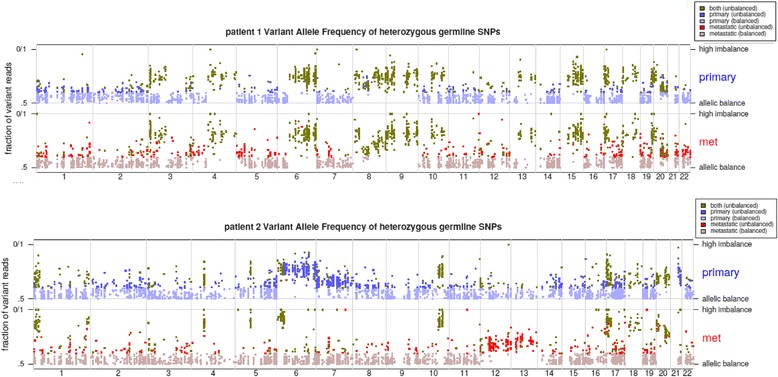
**Allellic imbalance.** B-allele frequency plots of heterozygous SNPs in tumor and normal tissue of patients 1 and 2. The y-axis indicates deviation of B-allele frequency from 0.5 towards either 0 or 1; the x-axis indicates chromosomal number.

Next, we examined if regions of allelic imbalance are similar or distinct in the primary and metastasis of the same patient. We identified heterozygous germline SNPs from each patient’s normal non-neoplastic tissue sample. In each patient, we identified a median of 1,986 SNPs (range 1,641 to 3,791). We examined the B-allele frequencies of these SNPs in the corresponding tumor or metastatic tissue of each patient, which we would expect to deviate substantially from 0.5 (that is, towards 0 or towards 1.0) in regions of allelic imbalance. We were able, therefore, to plot broad regions of allelic imbalance in each patient’s primary or metastatic tumor. Across the 18 patients, we found that broad regions of allelic imbalance were generally similar in the primary tumor and in the liver metastasis. In some patients, however, there were additional small regions of allelic imbalance private to either the primary or the metastasis. For example, in patient 1, regions of allelic imbalance were very similar in the primary and metastasis, while in patient 2, regions of allelic imbalance were generally similar, although allelic imbalance in chromosome 6q was observed in the primary and not in the metastasis (Figure 4). This similarity of regions of allelic imbalance in corresponding primary and metastasis further supports the linear progression model in patients with liver-limited mCRC.

### Focal gains and losses of genes commonly amplified or deleted in cancer are also similar in the primary and metastasis

We used the nCounter v2 Cancer CN Assay Kit, a highly multiplexed assay that enables copy number quantification for 87 genes commonly amplified or deleted in cancer to generate a copy number profiles for the primary, metastasis and normal tissues from the 18 patients (Additional file 7: Figure S2). We found that focal copy number gains and losses were also highly similar in the primary tumor and corresponding liver metastasis, in further support of the linear progression model in patients with liver-limited mCRC.

### Our data support linear progression in most patients with liver-limited mCRC

We have summarized the genomic concordance of the somatic SNVs, regions of allelic imbalance and focal amplifications or deletions of cancer-associated genes for each of the 18 patients in Additional file 7. Across the 18 patients, the large majority of variants are common to both the primary and the metastasis. VAFs of ‘shared’ variants were much higher than ‘private’ variants solely in the primary or metastasis. Broad regions of allelic imbalance are similarly present in matched primary and liver metastasis, although additional regions of allelic imbalance are observed ‘private’ to the primary or metastasis in some patients. Focal amplifications and deletions of cancer-associated genes are also similarly present in matched primary and liver metastasis with a smaller number of private copy number changes in either the primary or metastasis in some patients. Together, this supports a linear progression model with a thick common trunk where dominant clones from the primary tumor give rise to the metastasis and, within each tissue, further subclones found at lower frequencies begin to develop. Of interest, while there was a high degree of genomic concordance, we observed evidence that the mutational signatures characterize somatic mutations that are ‘shared’ versus those that are private, suggesting that different mutational processes may regulate different phases of cancer progression.

## Discussion

Our study compares genomic alterations across a large panel of cancer-associated genes in matched CRC primary tumors and liver metastasis. Using high-depth targeted NGS in matched primary CRC and liver metastases and dedicated bioinformatics algorithms, we comprehensively catalogued somatic variants across more than 750 cancer-related genes, including those present at low allele frequency. We validated the robustness of our approach with an orthogonal NGS technology (Ion Torrent) and found a high true positive rate (95%) and true negative rate (97%), although the latter is based on validation of identified variants and not all genes. Our study highlights the benefit and importance of using different variant-calling algorithms for comprehensive detection of somatic variants found in the primary or the metastasis. Only 39% of variants were called by all three algorithms and 23% of variants were only called by one algorithm. The sensitivity of each algorithm alone was 75.9% for GATK, 87.6% for LoFreq and 56.4% for Mutect. Several studies have documented varying sensitivities and concordance with multiple variant-calling algorithms and pipelines applied on the constant dataset [19-21]. This was the motivation behind using three variant calling algorithms in our analysis. Employing any one of the algorithms alone would lead to missing multiple variants and lead our study towards different conclusions. With high-depth sequencing, high coverage allows for high sensitivity in the tumor but it also leads to higher rates of false positive calls in the normal, leading to a potential loss in sensitivity in identifying somatic variants. Specific parameter settings and heuristic thresholds intrinsic to each algorithm lead to different predictions for some mutations. An in-depth analysis of factors that affect the various algorithms is outside the scope of our current paper and has been examined elsewhere [19-21]. Our analysis and validation results only support the use of three variant callers for the most comprehensive identification of somatic variants in comparative genomic studies.

We observed remarkable similarity among variants identified in the primary and the matched liver metastasis in each patient. More than three-quarters of alterations found in the primary were present in the metastasis. Similarly, more than three-quarters of alterations found in the metastasis were already present in the primary. In both the primary and metastasis, the remaining one-quarter of variants were unique to their respective sites and were generally of low allele frequency. Variants in genes recurrently altered within our dataset and variants found at higher allele frequency within the primary or metastasis were more likely to be present at the corresponding site.

As with lung cancer [14], the most well-described driver alterations in colorectal cancer (for example, ‘hotspot alterations’ in *KRAS*, *BRAF*, *NRAS* and *PIK3CA*) are concordant between primary tumors and metastatic lesions. *TP53* alterations are generally concordant provided there is minimal exposure to cytotoxic chemotherapy. Notably, 30% of the patients did not have any alterations in these five driver genes [22]. We performed a comprehensive approach with high-depth NGS across all exons of over 750 genes and 3 dedicated variant-calling algorithms. We comprehensively identified variants in each tissue, including those at low allele frequency. We found that across cancer related genes there remains a very high level of concordance between mutational profiles in matched primary CRC and liver metastases from patients undergoing surgery for liver-limited mCRC.

Several studies have compared the genomic profiles of matched primary and metastatic lesions and reported varying degrees of concordance. In kidney [9], breast [10], liver [23] and pancreatic cancer [11], 32%, 34%, 63% and 64% of mutations, respectively, were shared between the primary tumor and metastatic sites. In childhood acute lymphoblastic leukemia, subclones have variegated genetics and complex, branching evolutionary histories [12]. Epigenetically and genetically, medulloblastoma metastases are substantially diverged from the primary tumor [13]. There are two critical points to note in these studies. Firstly, they were generally performed in heavily pre-treated patients where substantial time had passed between the first emergence of metastasis and genomic profiling during which patients received cumulative cycles of chemotherapy and/or targeted therapy and further genomic alterations in the metastasis would likely have accumulated. Secondly, global characterization of the genome or epigenome was performed as opposed to focusing on sequence alterations across known cancer-related genes, which are more likely to be ‘driver’ alterations. Some of the variation observed by whole-genome analysis may be bystander mutations that are less relevant to disease biology; epigenetic alterations may also be more dynamic as opposed to sequence alterations.

Thus, comparative genomic studies must be interpreted in the context of the patient’s clinical and treatment history and the genomic platforms deployed. For instance, a genomic profiling study of paired primary and metastasis in non-small cell lung cancer [14] performed targeted NGS of 189 recurrent cancer-related genes and reported concordance of 94% for alterations recurrent across lung cancer and 63% for other alterations in cancer-related genes. Notably, these metastases were sampled and profiled when recurrence was first diagnosed so patients had limited systemic therapy except short duration adjuvant chemotherapy. Our study focused on patients with liver-limited mCRC, a unique population for whom the natural history of progression has shaped a distinctive management paradigm in clinical practice. Importantly, our patient cohort had synchronous or metachronous liver metastasis and surgery was performed with no prior systemic chemotherapy or in patients who have received a limited number of lines of previous cytotoxic chemotherapy regimens. Our study is not powered to detect small differences in genomic diversity amongst different subpopulations of patients with liver-limited mCRC. We did not observe major differences in genomic similarity between the primary and metastasis amongst patients with synchronous or metachronous tumors or amongst patients who received or did not receive prior systemic chemotherapy.

Varying reports of genomic divergence have been reported amongst patients with liver-limited mCRC. A recent study in liver-limited mCRC employed sequencing across the exome (which would not be at high sequencing depth) and high resolution copy number variation analysis across the genome and reported genomic concordance between the matched primary and metastases in half the patients but genomic divergence amongst paired tumors in half of the patients studied [24]. A study that performed shallow targeted sequencing (median coverage 87×) and only identified variants above 20% VAF reported substantial mutational divergence between paired primary and metastasis [25] while another more recent study that deeply sequenced 230 genes in matched tumors of 69 patients reported a high level of mutational concordance amongst non-synonymous mutations and indels [26]. In our study, we focused on 750 genes known to be biologically important in cancer rather than across the genome, performing sequencing to high depths (median depth 398×) and utilized three different variant-calling algorithms to identify low-frequency variants for greater sensitivity. When we focus on comprehensive characterization of cancer-related genes, we found a high degree of concordance between variants amongst primary and matched liver metastasis from all our patients with liver-limited mCRC. We also examined broad regions of allelic imbalance using information from over 1,000 SNPs per patient and documented focal copy number alterations of commonly amplified/deleted cancer genes. Thus, several lines of evidence support a linear progression model in colorectal cancer with branched evolution characterized by a common thick trunk before branching off a smaller set of alterations unique to the primary or the metastasis.

Our study provides biological insights into liver-limited mCRC. There is a fundamental difference in the natural progression of certain metastatic CRC tumors. Due to biologic characteristics not yet fully defined, certain metastatic lesions will be limited in number, slow growing, and potentially confined to the liver, thus rendering themselves targets for liver-directed therapy. We identified a high degree of concordance between sequence alterations in cancer-related genes as one of these characteristics. Whilst intra-tumoral heterogeneity and sampling might contribute to different estimates of genomic similarity, the distribution of allele frequencies amongst variants observed in our study population suggests that dominant primary and metastatic clones remain highly similar. The proportions of each variant relative to other variants in the primary and metastasis are also highly similar. This would go against tumor self-seeding where clones from the secondary may seed the primary but would form a smaller fraction of variants in the primary. Several models of cancer progression have been proposed [6]. For liver-limited mCRC, our findings support a model of linear progression [7] in liver-limited mCRC over that of parallel progression [8]. Dominant clones in metastasis closely resemble the primary. Thus, the acquisition of additional sequence alterations amongst cancer-associated genes does not appear necessary for establishment of metastases. Perhaps adaptation to anatomically different microenvironments, leading to establishment of metastasis, might be a result of transcriptomic or epigenetic changes rather than new sequence alterations. Beyond the dominant clones, in both the primary and the metastasis, a modest complement of genetic variants unique to each site, typically at low allele frequency, can also be found. This suggests that in each organ site, further subclones continue to develop independently. More extensive studies will be required to further characterize genomic progression and to substantiate linear progression as the dominant mode of cancer progression in this important clinical population of liver-limited mCRC. In particular, genomic analyses of multiple spatio-temporally separated samples within the same patient and evaluation of the impact of genomic progression on clinical phenotypes and outcomes, as has recently been performed in lung cancer, may provide a more detailed picture of clonal ancestry and tumor evolution during cancer progression [27,28]. Larger sample sizes, analyses of intra-tumoral heterogeneity and alterations in other levels of genomic diversity, such as evaluation of the epigenome and transcriptome, as well as studies in experimental systems may provide further insights into genomic alterations as cancer progresses in patients with liver-limited mCRC.

In our study, not every patient’s genomic profile is solely consistent with a linear progression model. In patient 15, three high VAF alterations (*KRAS*, *TP53* and *SMAD4*) are common to both primary and metastasis but the primary had 14 other mutations not found in the metastasis. Regions of allelic imbalance were common in patient 15’s paired primary and metastasis, although the metastasis had private regions of allelic imbalance in chromosome 8q. In patient 10, half of the mutations present in the primary and metastasis were private and there were also private focal amplifications of several genes.

The primary and metastasis of patient 16 (the *POLE* ultramutant) were separated by more than 6 years. Yet, the genomic similarity is very high at the non-synonymous SNV level. As is typical for *POLE* mutated tumors, this tumor was generally diploid with few regions of allelic imbalance and few genes focally amplified or deleted. In this patient, the same mutational signature was also observed in the primary and the metastasis, suggesting that the mutational processes regulated by *POLE* alterations are present in both the primary and the metastasis.

In the remaining patients who were microsatellite stable and did not have *POLE* alterations, despite the high degree of genomic concordance between the primary and metastasis, we are amongst the first to report evidence that the mutational signatures characterizing somatic mutations that are ‘shared’ between the primary and metastasis are different from the mutational signatures of mutations that are private to the metastasis. This suggests that different mutational processes may regulate different phases of cancer progression and that the mutational processes that occur before metastasis happens may be different from the mutational processes that occur at the site of the metastasis, perhaps due to its different micro-environment and tissue context. This biological insight could have potential impact on designing therapeutic strategies to target or prevent metastasis.

The aggressive multi-modality management of liver-limited mCRC has driven improvements in long-term disease control rates in mCRC. Clinical challenges remain, including the identification of novel therapeutic targets and selection of appropriate patients for aggressive multimodality therapy. It would be interesting to examine if the degree of genomic similarity might assist improved selection of patients with ‘better’ biology for aggressive multi-modality therapy. Specifically, the high degree of genomic similarity across cancer-associated genes between the primary and metastasis in patients with liver-limited mCRC raises the possibility that key somatic alterations identified in either tissue may provide relevant and sufficient genomic information to guide treatment decisions in this clinical subset. With largely similar molecular vulnerabilities in both tissues, pharmacologic intervention should work or not work equally well at both sites.

## Conclusions

We analyzed high-depth targeted NGS data of over 750 cancer-associated genes, using three variant-calling algorithms to comprehensively identify somatic variants from matched primary and metastatic tumors of 18 patients with liver-limited mCRC. We also performed analysis to identify regions of allelic imbalance and copy number profiling of 89 cancer-associated genes. Through our analysis, we found high genomic concordance between primary tumors and metastases, in support of the linear progression model in liver-limited mCRC.

## Materials and methods

### Patient selection

We obtained matched primary CRCs, liver metastases, and adjacent normal tissue for 18 patients from our institution’s frozen tissue repository. All patients signed written informed consent to donate their tissue samples for research. Our study was approved by Singhealth Institutional Review Board ‘Understanding in the evolution of Ca with next generation genomic tools in patients with synchronous, metachronous or metastatic neoplasms’ 2011/439/B. The experimental procedures in this study comply with the Helsinki Declaration. Clinico-pathologic information is summarized in Table 1. A pathologist performed cryosectioning analysis to ascertain adequate tumor content in each tumor sample and absence of tumor in the normal sample.

### Targeted sequencing of cancer-associated genes

We performed a comprehensive literature and database review to identify genes biologically and clinically relevant to cancer, including genes involved in key oncogenic signaling pathways, oncogenes, tumor suppressor genes and genes from kinase and chromatin remodeler families. Data repositories including the Catalogue of Somatic Mutations in Cancer (COSMIC) [29,30], mutations of kinases in cancer (MokCa), DNA tumor suppressor and Oncogene Database and Cancer Gene Census [31] were also mined to identify genes exhibiting recurrent somatic mutations in cancer. Two generations of targeted panels were employed in our study, the ‘750 panel’ and ‘800 panel’. The ‘750 panel’ comprised all exons of 766 cancer-related genes. The later generation ‘800 panel’ comprised all exons of 799 cancer-related genes and 87 introns of 28 genes involved in somatic translocations. This report focuses on alterations found in exons of cancer-related genes. A list of genes is provided in Additional file 8. We used the Agilent SureSelect E-array software to design unique RNA baits employing 41,628 and 57,682 baits for the ‘750’ and ‘800’ panels, respectively. Biotinylated RNA baits were synthesized by Agilent for the SureSelect Target Enrichment system (Agilent Technologies Santa Clara California, USA).

### DNA extraction, capture enrichment and library construction

We extracted DNA from tissue specimens with the Genomic DNA extraction kit (Qiagen Venio, Netherlands). Extracted DNA was evaluated for quality, yield and concentration. DNA samples were sheared using a Covaris S2 (Covaris: Woburn Massachusettes, USA) to a size distribution (150 to 200 bp) optimal for target enrichment. Size-selected adapter-ligated libraries were incubated with the custom-designed SureSelect baits for 24 h. Following capture, we performed further cycles of DNA amplification. Samples successfully meeting the size and concentration criteria were pooled at equimolar concentrations. Up to six samples with unique index-tag adapter sequences were combined for multiplex NGS in each lane on the Illumina HiSeq 2000 (Illumina, San Diego, California, USA). Short reads from our study has been deposited in the European Nucleotide Archive (ENA) under study accession ID PRJEB7714.

### Bioinformatics and variant detection

Our bioinformatics pipelines to identify somatic variants involved three algorithms, GATK, LoFreq and MuTect.

#### Genome Analyzer Toolkit-based algorithm

We aligned sequence reads to the human reference genome (hg19) and removed PCR duplicates. GATK [15] was used for consensus calling to identify and filter SNVs. We recalibrated base qualities (CountCovariates and TableRecalibration modules), realigned around microindels (RealignerTargetCreator, IndelRealigner) and called variants (IndelGenotyperV2, UnifiedGenotyper, VariantFiltration). We also filtered the variant list to remove any variants if the following criteria were true: (1) two variants within a window of 5 bases; (2) variant was in a homopolymer run >5; (3) ‘strand bias’ score > −0.10; (4) mapping quality <30.0; and (5) depth <5. For a variant to be called as somatic, these additional filter had to be passed: (1) minimum quality/depth = 3; (2) minimum variant depth > 2; and (3) minimum depth in normal ≥5.

#### LoFreq

LoFreq [16] is a sensitive variant caller designed to call low-frequency variants by exploiting base-call qualities. To identify variants present at low allele frequency, we employed LoFreq (lofreq-v0.5.0) using the default settings on the same realigned BAM file generated by the GATK-based algorithm. The SNVs identified were annotated using the Variant Effect Predictor (VEP, v2.8). Only missense, nonsense and splice site SNVs were retained. SNVs that were present in dbSNPv135 were removed unless they were also present in COSMIC. To determine somatic SNVs, the tumor or metastatic sample was compared with the normal sample and only the SNVs that were unique to the tumor or metastatic sample were retained.

#### MuTect

MuTect [17] is a sensitive somatic variant caller that applies a Bayesian classifier with tuned filters to retain high specificity. To identify somatic variants present at low allele frequency, we employed MuTect (muTect-1.1.4) using the default settings on the same realigned BAM file generated by the GATK-based algorithm. The SNVs identified were annotated using the Variant Effect Predictor (VEP, v2.8) [32]. Only missense, nonsense and splice site SNVs were retained. SNVs that were present in dbSNPv135 were removed unless they were also present in COSMIC.

We curated variants called by at least one of the above algorithms by manual inspection of the sequencing reads corresponding to each position with the Integrative Genomics Viewer Browser. Gene transcript annotation databases (CCDS [33], RefSeq [34], Ensembl [35], UCSC Known Genes [36]) were used for transcript identification and to determine amino acid changes. Amino acid changes corresponding to SNVs were annotated according to the largest transcript of the gene.

### Validation of somatic variants with Ion Torrent

Ion Torrent custom primers were designed by Ampliseq primer design software. Libraries were prepared using Ion Ampliseq Library kit (Life Technologies, Guilford, Connecticut). DNA (10 ng) was taken and the targets were PCR amplified with the appropriate primer pool; the primers were later partially digested and adaptor ligated. The adaptor ligated libraries were then purified by AMpure beads and the concentration was quantified by Bioanalyser (Life Technologies, Guilford, Connecticut). Samples were pooled and prepared for sequencing using the Ion PGM 200 Sequencing Kit (Ion Torrent) protocol. Pooled samples were loaded on the 318 chip and sequenced on the Ion Torrent PGM 200 (Life Technologies, Guilford, Connecticut) for 125 cycles. Data processing, filtering and base calling were done using the Ion Torrent server, Torrent Suite v3.6.5.

### Identification of regions of allelic imbalance

We identified positions that are heterozygous in the normal tissue and with a minimum depth of 20 in the normal. Allelic imbalances in tumor samples are observed in B Allele Frequency (BAF) plots as a deviation from 0.5 of SNPs heterozygous in cells with constitutional genotype.

### Detection of copy number variation in cancer-associated genes

nCounter Cancer Copy Number Variation CodeSets were used with 300 ng purified genomic DNA extracted from frozen tissue (extracted as described above). DNA was fragmented via AluI digestion and denatured at 95°C. Fragmented DNA was hybridized with the codeset of 89 genes in the nCounter Cancer CN Assay Kit v2 (Nanostring, Seattle, Washington) for 18 hours at 65°C and processed according to the manufacturer’s instructions. The nCounter Digital Analyzer counted and tabulated the signals of reporter probes and average count numbers of >3 were called and confirmed by immunohistochemistry, FISH (fluorescence *in situ* hybridization) or real-time PCR.

## Additional files

Additional file 1: Table S1. Sequencing coverage across the 54 tissue specimens. Additional file 2: Figure S1. Distribution of variants. Venn diagram of variants called by the three bioinformatics algorithms: GATK-based algorithm (blue), LoFreq (purple) and Mutect (red). Information of the variant-allele frequency (VAF) of the variants called by the various algorithms and the true positive (TP) rate is provided in the Venn diagram. Additional file 3: Table S2. Variant allele frequency and coverage for variants identified by only one algorithm. Additional file 4: Table S3. List of 1,236 distinct variants identified in 18 patients. Additional file 5: Table S4. Recurrent variants and their distribution in the primary tumor and metastasis. Additional file 6: Table S5. Ingenuity Pathway Analysis pathways identified that are unique to shared variants or to common variants. Additional file 7: Figure S2. Aggregate genomic alterations in each of the 18 patients. Top panel: somatic non-synonymous alterations in the primary (blue) and metastasis (red). Middle panel: B-allele frequency plots of heterozygous SNPs in tumor and normal tissue. The y-axis indicates deviation of B-allele frequency from 0.5 towards either 0 or 1; the x-axis indicates chromosomal number. Bottom panel: focal copy number estimates from Nanostring nCounter Cancer Copy Number v2 panel in the primary and metastasis. The y-axis indicates estimated copy number; the x-axis indicates chromosomal number. Additional file 8: Table S6. Description of Targeted NGS panel. List of 799 cancer-associated genes selected for targeted next generation sequencing.

## Abbreviations

CRC: colorectal cancer
GATK: Genome Analyzer Toolkit
mCRC: metastatic colorectal cancer
IPA: Ingenuity Pathway Analysis
NGS: next generation sequencing
PCR: polymerase chain reaction
SNP: single-nucleotide polymorphism
SNV: single nucleotide variant
VAF: variant allele frequency
