# Supplementary material for: High-depth sequencing of over 750 genes supports linear progression of primary tumors and metastases in most patients with liver-limited metastatic colorectal cancer
Source: Genome Biol. 2015 Feb 12;16(1):32. doi: 10.1186/s13059-015-0589-1 (PMC4365969; doi:10.1186/s13059-015-0589-1)
Supplement: Additional file 1: Table S1. — Sequencing coverage across the 54 tissue specimens. [file 13059_2015_589_MOESM1_ESM.pdf]

Supplementary table 1: Sequencing coverage across the 54 Tissue Specimens.

Patient		Median Coverage	Mean Coverage	% Target Bases with > 20x coverage	% Target Bases with > 100x coverage	% Target Bases with > 200x coverage
Pat 1	T	487	545	99.2%	95.9%	87.3%
	Met	456	512	99.1%	94.4%	84.0%
	Normal	303	330	99.0%	91.4%	72.9%
Pat 2	T	508	553	99.1%	95.3%	86.8%
	Met	512	561	98.1%	94.4%	86.7%
	Normal	564	601	99.2%	95.9%	88.6%
Pat 3	T	677	751	99.2%	96.5%	90.5%
	Met	508	555	99.2%	95.4%	86.8%
	Normal	505	547	99.2%	95.2%	86.4%
Pat 4	T	599	649	99.2%	96.0%	89.1%
	Met	502	552	99.1%	94.8%	85.4%
	Normal	539	576	99.0%	95.4%	87.5%
Pat 5	T	289	296	98.1%	89.0%	70.7%
	Met	586	598	98.7%	95.8%	90.0%
	Normal	339	335	98.2%	91.1%	76.8%
Pat 6	T	490	495	98.7%	94.7%	86.6%
	Met	330	351	98.2%	90.9%	75.2%
	Normal	317	316	98.2%	90.1%	73.8%
Pat 7	T	214	222	97.4%	82.6%	54.2%
	Met	389	427	98.4%	92.0%	79.3%
	Normal	332	330	98.1%	90.3%	74.9%
Pat 8	T	413	469	98.2%	91.8%	80.3%
	Met	318	331	97.6%	87.0%	71.1%
	Normal	380	384	97.9%	90.0%	77.0%
Pat 9	T	377	403	98.2%	91.1%	78.4%
	Met	444	460	98.6%	93.6%	83.9%
	Normal	417	423	98.4%	93.1%	82.6%
Pat 10	T	357	379	98.0%	89.6%	75.9%
	Met	454	490	98.4%	93.2%	83.3%
	Normal	420	427	98.5%	93.0%	82.3%
Pat 11	T	285	307	97.5%	86.4%	67.5%
	Met	323	341	97.9%	89.3%	73.5%
	Normal	392	398	98.3%	92.0%	80.1%
Pat 12	T	272	289	97.8%	86.4%	66.3%
	Met	204	213	97.4%	82.0%	51.3%
	Normal	315	317	98.1%	89.2%	73.1%
Pat 13	T	358	385	98.1%	90.8%	76.8%
	Met	118	131	95.0%	59.4%	18.2%
	Normal	357	363	98.3%	91.3%	77.7%
Pat 14	T	386	409	98.3%	92.1%	79.6%
	Met	292	309	98.2%	89.1%	70.4%
	Normal	364	372	98.2%	91.1%	77.6%
Pat 15	T	512	537	98.6%	94.3%	86.1%
	Met	435	456	98.5%	93.2%	82.9%

	Normal	419	426	98.2%	92.4%	81.8%
Pat 16	T	307	315	98.0%	88.8%	71.8%
	Met	376	386	98.3%	91.2%	78.2%
	Normal	306	312	98.1%	89.2%	72.3%
Pat 17	T	339	359	98.1%	90.6%	75.9%
	Met	344	363	98.2%	91.0%	76.7%
	Normal	314	320	98.2%	90.1%	74.0%
Pat 18	T	344	364	98.3%	91.3%	77.1%
	Met	352	379	98.2%	90.5%	76.4%
	Normal	282	289	98.0%	88.3%	69.0%
