# Supplementary figures and images for: High-depth sequencing of over 750 genes supports linear progression of primary tumors and metastases in most patients with liver-limited metastatic colorectal cancer
Source: Genome Biol. 2015 Feb 12;16(1):32. doi: 10.1186/s13059-015-0589-1 (PMC4365969; doi:10.1186/s13059-015-0589-1)

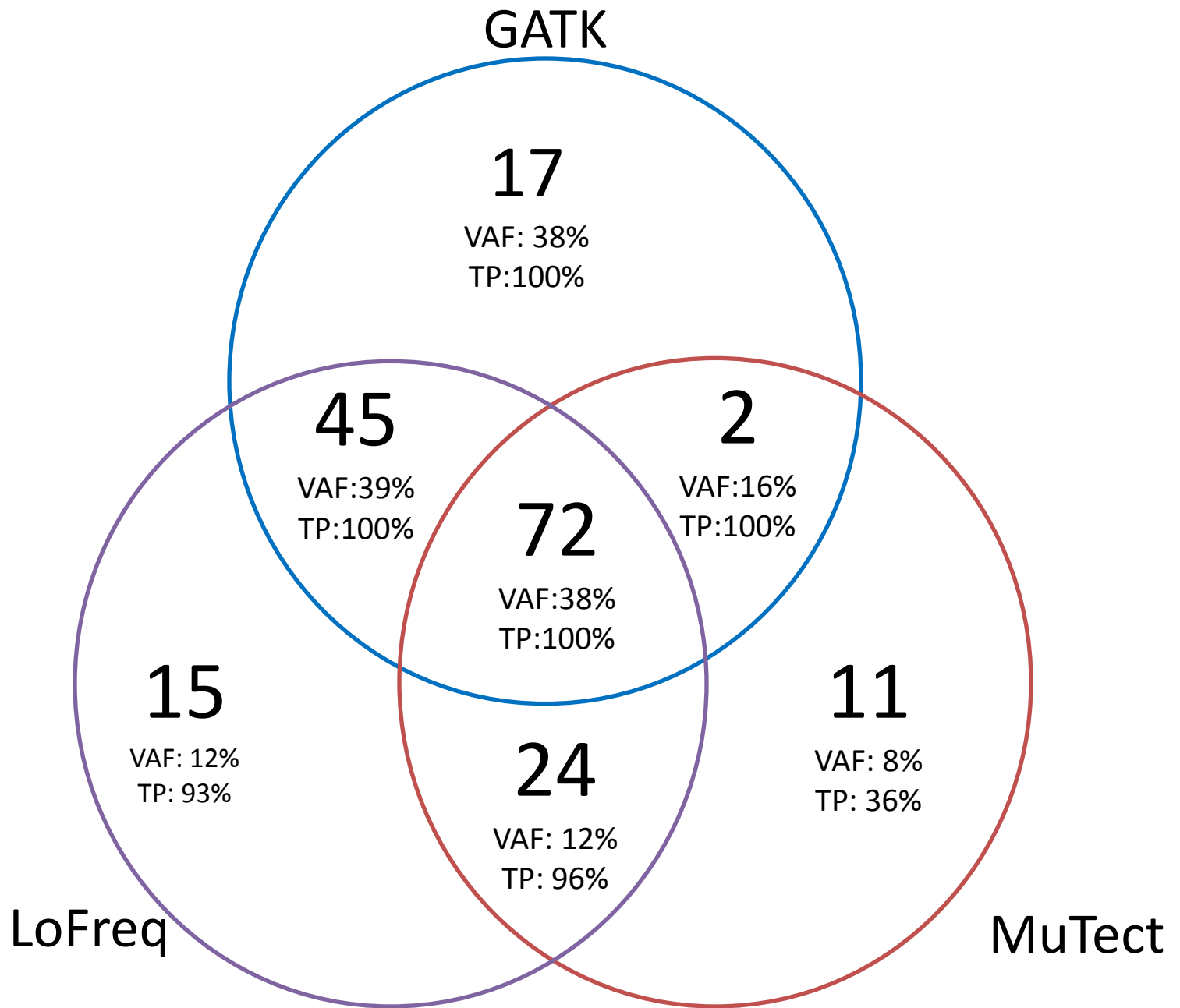

Supplement: Additional file 2: Figure S1. — Distribution of variants. Venn diagram of variants called by the three bioinformatics algorithms: GATK-based algorithm (blue), LoFreq (purple) and Mutect (red). Information of the variant-allele frequency (VAF) of the variants called by the various algorithms and the true positive (TP) rate is provided in the Venn diagram. [file 13059_2015_589_MOESM2_ESM.pdf]
