# Supplementary material for: High-depth sequencing of over 750 genes supports linear progression of primary tumors and metastases in most patients with liver-limited metastatic colorectal cancer
Source: Genome Biol. 2015 Feb 12;16(1):32. doi: 10.1186/s13059-015-0589-1 (PMC4365969; doi:10.1186/s13059-015-0589-1)
Supplement: Additional file 3: Table S2. — Variant allele frequency and coverage for variants identified by only one algorithm. [file 13059_2015_589_MOESM3_ESM.pdf]

Supplementary table 2: Variant allele frequency and coverage for variants identified only by 1 algorithm

Algorithm	Tumor	Normal
GATK	0.330	0.002
LoFreq	0.060	0.004
MuTect	0.040	0.004

Algorithm	Tumor	Normal
GATK	267	257
LoFreq	625	396
MuTect	529	275
