# Supplementary material for: High-depth sequencing of over 750 genes supports linear progression of primary tumors and metastases in most patients with liver-limited metastatic colorectal cancer
Source: Genome Biol. 2015 Feb 12;16(1):32. doi: 10.1186/s13059-015-0589-1 (PMC4365969; doi:10.1186/s13059-015-0589-1)
Supplement: Additional file 4: Table S3. — List of 1,236 distinct variants identified in 18 patients. [file 13059_2015_589_MOESM4_ESM.pdf]

Supplementary Table 3: List of 1236 variants in 18 patients

Number	Patient	Gene	Amino acid Change	Distribution of variant	Allele Frequency in primary	Allele Frequency in metastasis
1	1	EPHA8	R131H	Met Only	0	0.12
2	1	MSH2	L529V	Primary Only	0.04	0
3	1	ERBB4	R927*	Shared	0.23	0.36
4	1	ERBB4	K592T	Primary Only	0.07	0
5	1	KDR	L277I	Primary Only	0.02	0
6	1	APC	E1306*	Shared	0.31	0.39
7	1	APC	S1392*	Shared	0.31	0.37
8	1	GLI3	R248H	Shared	0.33	0.36
9	1	TRRAP	E1384Q	Primary Only	0.03	0
10	1	CDKN2A	P94L	Shared	0.11	0.64
11	1	TP53	H214R	Shared	0.5	0.64
12	1	CDC27	L15F	Primary Only	0.04	0
13	1	CBLC	D46N	Primary Only	0.07	0
14	1	GNAS	5’ splice	Met Only	0	0.23
15	1	MN1	A991T	Shared	0.36	0.53
16	2	KDM5B	Q1378E	Shared	0.23	0.43
17	2	ALK	R1209*	Shared	0.42	0.382463
18	2	ERBB4	R393W	Shared	0.39	0.45
19	2	PIK3CB	R994Q	Shared	0.22	0.371747
20	2	LPP	G147S	Primary Only	0.03	0
21	2	APC	R805*	Shared	0.22	0.45
22	2	APC	G1365*	Shared	0.12	0.122931
23	2	HSP90AB	R405C	Shared	0.040419	0.412963
24	2	RPS6KA2	R718Q	Shared	0.47	0.24
25	2	PTEN	R335*	Shared	0.49	0.78
26	2	KRAS	A146T	Shared	0.45	0.6
27	2	COL1A1	R244H	Shared	0.15	0.43
28	3	TNFRSF1	S41T	Shared	0.28	0.24
29	3	SDCCAG8	T21P	Shared	0.55	0.31
30	3	XIRP2	E208*	Met Only	0	0.19
31	3	APC	Q1406*	Shared	0.28	0.17
32	3	AC00560	R7*	Shared	0.33	0.19
33	3	GLI3	L1469P	Shared	0.62	0.35
34	3	TRRAP	Q2496E	Shared	0.55	0.36
35	3	CSMD3	R1946C	Shared	0.39	0.14
36	3	EP400	P2549L	Met Only	0	0.1
37	3	USP6	R606W	Shared	0.7	0.37
38	3	TP53	5’ splic	Shared	0.67	0.43
39	3	SOX3	R196C	Shared	0.89	0.59
40	4	PIK3CA	K720Q	Primary Only	0.07	0

41	4	FAT4	T1319K	Shared	0.08	0.23
42	4	WHSC1	V1166I	Met Only	0	0.03
43	4	APC	splice d	Shared	0.07	0.25
44	4	CSMD3	S932C	Primary Only	0.05	0
45	4	ATM	R2034*	Shared	0.16	0.54
46	4	LRRK2	K2278N	Shared	0.06	0.22
47	4	BRCA1	R613S	Shared	0.08	0.27
48	4	SOX9	Q439*	Shared	0.06	0.3
49	4	MLLT1	T429S	Shared	0.07	0.44
50	4	MAP2K7	V30L/splice	Shared	0.14	0.47
51	4	PIK3R2	G373R	Shared	0.08	0.24
52	4	GUCY2F	T895N	Primary Only	0.12	0
53	5	PRDM2	S1256F	Primary Only	0.216912	0
54	5	EPHA6	E306K	Primary Only	0.042079	0
55	5	PIK3CA	E542K	Shared	0.331169	0.202514
56	5	EVC2	T621N	Shared	0.315534	0.426606
57	5	CTNND2	A500T	Shared	0.246032	0.285211
58	5	APC	E1309*	Primary Only	0.152334	0
59	5	APC	K1363*	Shared	0.428884	0.650386
60	5	MAD1L1	R570Q	Shared	0.217391	0.347222
61	5	GRM8	E500K	Shared	0.220833	0.163978
62	5	CSMD3	V1453F	Shared	0.319444	0.405983
63	5	DLC1	R387W	Met Only	0	0.195205
64	5	STK33	3'splice	Met Only	0	0.221024
65	5	KRAS	Q61L	Shared	0.379581	0.285714
66	5	PTPN11	A72V	Shared	0.168675	0.236479
67	5	ADAMTSL	Q1458K	Shared	0.346705	0.458658
68	5	TP53	V216M	Shared	0.4791	0.5919
69	6	ACVR2A	T27N	Met Only	0	0.186555
70	6	EPHB1	C196F	Primary Only	0.036496	
71	6	ETV5	L80F	Shared	0.270992	0.233871
72	6	APC	E190*	Shared	0.241993	0.540501
73	6	APC	S1327*	Shared	0.196429	0.350202
74	6	PCDHA13	A592V	Shared	0.218391	0.341317
75	6	BAI3	G594E	Met Only	0	0.162722
76	6	CARD11	5'splice	Shared	0.256757	0.384164
77	6	CSMD3	L461F	Primary Only	0.057018	0
78	6	MELK	V287I	Shared	0.200803	0.189904
79	6	PTCH1	R40L	Met Only	0	0.153846
80	6	GUCY1A2	K128N	Shared	0.240559	0.364341
81	6	DDX10	K615*	Shared	0.059499	0.208743
82	6	NAV3	L33I	Shared	0.190291	0.401709
83	6	GNA11	A304V	Shared	0.240803	0.49537
84	6	ASXL1	E802*	Met Only	0	0.03
85	6	PTPRT	R1395H	Met Only	0	0.072658
86	7	TIE1	R91C	Shared	0.333333	0.25

87	7	REL	K82N	Met Only	0	0.036215
88	7	CXCR7	S335A	Met Only	0	0.178117
89	7	PIK3CA	N345K	Shared	0.559387	0.80597
90	7	FBXW7	R465H	Shared	0.596825	0.813264
91	7	FAT1	C4028F	Met Only	0	0.215328
92	7	APC	S1356*	Shared	0.610345	0.813953
93	7	FNDC1	K133N	Met Only	0	0.035354
94	7	CSMD1	R2943H	Shared	0.425	0.6633
95	7	PRKDC	G1282	Met Only	0	0.162338
96	7	ATM	L2332R	Met Only	0	0.135171
97	7	NAV3	S1534L	Shared	0.322314	0.55137
98	7	CDH11	Y90*	Primary Only	0.303754	0
99	7	TP53	R196*	Shared	0.488281	0.742671
100	7	ERBB2	A763V	Primary Only	0.134146	0
101	7	POLD1	3'splice	Shared	0.312849	0.463768
102	8	GATA1	R216W	Shared	0.9	0.526667
103	8	CBLB	Y363H	Shared	0.59	0.208431
104	8	FAT1	UTR	Shared	0.42	0.42
105	8	APC	S1190X	Shared	0.79	0.27577
106	8	TP53	R213X	Shared	0.83	0.260771
107	8	MAP4K5	E799V	Met Only	0	0.148248
108	9	DCLK1	E410K	Shared	0.30	0.32
109	9	XIRP2	A3019V	Shared	0.41	0.21
110	9	APC	R213X	Shared	0.67	0.29
111	9	BAI3	3’splice	Shared	0.41	0.35
112	9	GPC6	I176T	Met Only	0.00	0.07
113	10	BRDT	N252I	Shared	0.69	0.81
114	10	TP53	F113I	Shared	0.66	0.77
115	10	SMARCA4	P262S	Shared	0.17	0.29
116	10	APC	R302X	Shared	0.63	0.79
117	10	PCDHA13	E101D	Met Only	0.00	0.33
118	10	NSD1	3’splice	Met Only	0.00	0.31
119	10	GLI3	H147N	Shared	0.43	0.32
120	10	KDR	3’splice	Primary Only	0.03	0.00
121	10	MAST4	P26T	Primary Only	0.03	0.00
122	10	CXCR7	W67L	Primary Only	0.03	0.00
123	10	PRCC	V27F	Met Only	0.00	0.04
124	10	ERC1	E356D	Met Only	0.00	0.04
125	10	MAP3K3	V30F	Met Only	0.00	0.04
126	10	TLX1	G124W	Primary Only	0.10	0.00
127	10	NOTCH1	G996C	Primary Only	0.30	0.00
128	11	PRDM16	V629M	Shared	0.52	0.35
129	11	TP53	C242S	Shared	0.76	0.62
130	11	MAFB	S114X	Shared	0.63	0.45
131	11	PREX1	P986H	Met Only	0.00	0.19
132	11	PARK2	5’splice	Shared	0.35	0.23

133	11	AKAP9	Q1112X	Shared	0.30	0.25
134	11	CSMD1	5’splice	Shared	0.71	0.58
135	11	CASK	R537X	Primary Only	0.27	0.00
136	11	ITK	H217Y	Primary Only	0.04	0.00
137	12	MLL	R2627H	Shared	0.32	0.33
138	12	TP53	R196X	Shared	0.56	0.53
139	12	APC	R805X	Shared	0.35	0.35
140	12	GOPC	R183I	Shared	0.31	0.32
141	12	NDRG1	A268T	Shared	0.60	0.56
142	12	GATA1	M234I	Shared	0.19	0.30
143	12	ELF4	T426M	Shared	0.29	0.33
144	12	FAT4	P1527T	Primary Only	0.04	0.00
145	13	ARID2	D1523N	Shared	0.39	0.54
146	13	MAP2K1	K57N	Shared	0.55	0.95
147	13	CTSH	N177Y	Met Only	0.00	0.29
148	13	HLF	R215H	Primary Only	0.18	0.00
149	13	SCN5A	5’splice	Shared	0.33	0.61
150	13	MITF	R341C	Shared	0.41	0.69
151	13	FAT4	T845I	Shared	0.35	0.26
152	13	APC	R805X	Shared	0.39	0.48
153	13	NEK11	I317K	Primary Only	0.14	0.00
154	13	XIRP2	Q2554K	Primary Only	0.05	0.00
155	13	CSMD3	L346P	Primary Only	0.03	0.00
156	13	DCLK1	T425P	Primary Only	0.10	0.00
157	13	ENSG00000223911	Q54K	Primary Only	0.10	0.00
158	13	JAK1	C1131F	Met Only	0.00	0.04
159	13	XIRP2	D2651E	Primary Only	0.05	0.00
160	13	FLI1	N239K	Primary Only	0.03	0.00
161	13	SPEN	Q1595K	Primary Only	0.04	0.00
162	13	NAV3	C8F	Primary Only	0.05	0.00
163	13	PRKDC	Q1466H	Met Only	0.00	0.04
164	14	ARID5B	S451R	Primary Only	0.04	0.00
165	14	JAK1	3’splice	Shared	0.43	0.21
166	14	CENPF	A2371E	Primary Only	0.04	0.00
167	14	NAV2	R298C	Shared	0.40	0.34
168	14	TP53	R337C	Met Only	0.00	0.50
169	14	SMAD4	W101C	Shared	0.57	0.51
170	14	NFE2L2	P393S	Shared	0.38	0.34
171	14	CHD6	A1036E	Primary Only	0.03	0.00
172	14	PIK3CA	R38C	Met Only	0.00	0.33
173	14	CSF1R	G297D	Primary Only	0.09	0.00
174	14	KRAS	G12V	Shared	0.33	0.33
175	14	FAT4	T2427K	Shared	0.04	0.03
176	14	DDX10	A280E	Shared	0.03	0.04
177	14	N4BP2	N659K	Shared	0.03	0.04

178	14	PRDM1	Q94K	Met Only	0.00	0.04
179	14	XIRP2	D2651E	Shared	0.03	0.03
180	14	HEY1	C66F	Primary Only	0.03	0.00
181	14	TET2	Q373K	Shared	0.03	0.04
182	14	SLC9A9	Y106X	Met Only	0.00	0.06
183	14	TOP2B	T336K	Met Only	0.00	0.04
184	14	MAP3K3	V30F	Shared	0.05	0.03
185	14	ATRX	S1895R	Met Only	0.00	0.06
186	14	BRCA2	N433K	Met Only	0.00	0.03
187	15	ARID1A	G1157E	Primary Only	0.03	0.00
188	15	PAX2	S317N	Primary Only	0.05	0.00
189	15	TCF7L2	Y243X	Primary Only	0.30	0.00
190	15	ZBTB16	L119Q	Primary Only	0.13	0.00
191	15	KRAS	G12V	Shared	0.49	0.30
192	15	DCLK1	K57M	Primary Only	0.08	0.00
193	15	LCP1	V162D	Primary Only	0.25	0.00
194	15	FOXG1	R302H	Primary Only	0.06	0.00
195	15	CDH11	T639S	Primary Only	0.08	0.00
196	15	TP53	C141Y	Shared	0.85	0.50
197	15	SMAD4	E538X	Shared	0.82	0.48
198	15	FN1	Y2190X	Primary Only	0.04	0.00
199	15	EPHA3	V743G	Primary Only	0.14	0.00
200	15	FAT4	S670P	Primary Only	0.55	0.00
201	15	PREX2	K905T	Primary Only	0.13	0.00
202	15	HUWE1	S2093R	Primary Only	0.04	0.00
203	15	PTPRT	A731T	Primary Only	0.44	0.00
204	16	PTEN	K80N	Met Only	0.00	0.18
205	16	PTEN	R173H	Met Only	0.00	0.19
206	16	PTEN	E299X	Shared	0.18	0.24
207	16	PTEN	D395Y	Met Only	0.00	0.16
208	16	KRAS	N116H	Shared	0.12	0.16
209	16	KRAS	E98X	Shared	0.10	0.21
210	16	POLE	S1930X	Shared	0.12	0.14
211	16	POLE	M1819T	Shared	0.14	0.22
212	16	POLE	F959L	Primary Only	0.11	0.00
213	16	POLE	R680C	Met Only	0.00	0.25
214	16	POLE	A456P	Shared	0.13	0.20
215	16	PDGFRA	E34K	Shared	0.09	0.15
216	16	PDGFRA	E387K	Shared	0.08	0.19
217	16	PDGFRA	E732X	Shared	0.13	0.21
218	16	PDGFRA	A766T	Shared	0.09	0.17
219	16	KIT	E228K	Met Only	0.00	0.04
220	16	KIT	S709A	Shared	0.06	0.24
221	16	APC	5’splice	Primary Only	0.06	0.00
222	16	APC	S751Y	Met Only	0.00	0.19
223	16	APC	L795I	Primary Only	0.08	0.00

224	16	APC	R1114X	Shared	0.17	0.13
225	16	APC	E1544X	Shared	0.18	0.31
226	16	APC	K2050N	Shared	0.16	0.29
227	16	APC	K2189E	Shared	0.08	0.12
228	16	PRDM16	5'splice	Met Only	0.00	0.22
229	16	PRDM16	R986H	Shared	0.10	0.20
230	16	PRDM16	E1033D	Primary Only	0.10	0.00
231	16	PRDM16	N1078K	Shared	0.09	0.18
232	16	PRDM16	T1090M	Shared	0.09	0.16
233	16	PRDM16	S1265L	Shared	0.09	0.24
234	16	RPL22	K84T	Shared	0.13	0.17
235	16	CAMTA1	D11N	Shared	0.12	0.17
236	16	CAMTA1	E92X	Shared	0.10	0.21
237	16	CAMTA1	E693D	Shared	0.11	0.19
238	16	CAMTA1	R1225C	Met Only	0.00	0.15
239	16	MTOR	R2505X	Shared	0.10	0.18
240	16	MTOR	L67R	Shared	0.10	0.19
241	16	TNFRSF8	R417W	Shared	0.08	0.20
242	16	PRDM2	V618I	Met Only	0.00	0.18
243	16	SPEN	M1I	Shared	0.10	0.15
244	16	SPEN	E1043X	Shared	0.09	0.18
245	16	SPEN	S1857F	Shared	0.09	0.20
246	16	SPEN	K2024N	Met Only	0.00	0.20
247	16	FGR	K464T	Shared	0.10	0.21
248	16	SFPQ	5'splice	Shared	0.11	0.19
249	16	SFPQ	E325X	Shared	0.13	0.19
250	16	CLSPN	R1139Q	Met Only	0.00	0.17
251	16	CLSPN	R1139X	Met Only	0.00	0.15
252	16	CLSPN	K385Q	Shared	0.10	0.18
253	16	CLSPN	E90D	Met Only	0.00	0.16
254	16	THRAP3	R168Q	Shared	0.08	0.19
255	16	THRAP3	R507I	Shared	0.14	0.19
256	16	THRAP3	E695X	Shared	0.11	0.19
257	16	MACF1	P18S	Shared	0.08	0.17
258	16	MACF1	Q2853K	Shared	0.08	0.18
259	16	MACF1	T3161A	Shared	0.07	0.18
260	16	MACF1	S2047F	Shared	0.12	0.22
261	16	MACF1	R4538X	Shared	0.16	0.18
262	16	MACF1	L4643R	Primary Only	0.03	0.00
263	16	MACF1	N5237S	Shared	0.12	0.27
264	16	TIE1	S721I	Primary Only	0.07	0.00
265	16	MPL	A444S	Met Only	0.00	0.33
266	16	STIL	D830N	Met Only	0.00	0.24
267	16	EPS15	N812T	Met Only	0.00	0.19
268	16	EPS15	S470P	Shared	0.08	0.16
269	16	JAK1	N5Y	Shared	0.10	0.17

270	16	IL23R	Q487H	Shared	0.08	0.16
271	16	IL23R	Q555K	Primary Only	0.11	0.00
272	16	BRDT	E247X	Shared	0.10	0.19
273	16	BRDT	K459N	Shared	0.07	0.20
274	16	BRDT	E462X	Shared	0.06	0.16
275	16	BRDT	E874X	Shared	0.12	0.16
276	16	RBM15	D332E	Shared	0.10	0.17
277	16	MAGI3	R1285W	Shared	0.09	0.20
278	16	MAGI3	K1386N	Met Only	0.00	0.19
279	16	TRIM33	K998N	Primary Only	0.03	0.00
280	16	TRIM33	R865X	Shared	0.10	0.20
281	16	TRIM33	R759K	Met Only	0.00	0.20
282	16	FAM46C	E238X	Shared	0.11	0.21
283	16	NOTCH2	R1726C	Shared	0.11	0.22
284	16	NOTCH2	K784N	Met Only	0.00	0.19
285	16	NOTCH2	N425S	Shared	0.08	0.19
286	16	PDE4DIP	E2273X	Met Only	0.00	0.06
287	16	PDE4DIP	R1605H	Shared	0.05	0.07
288	16	PDE4DIP	R901Q	Shared	0.10	0.22
289	16	PDE4DIP	R634Q	Met Only	0.00	0.09
290	16	PDE4DIP	S10N	Met Only	0.00	0.09
291	16	PDE4DIP	R430Q	Shared	0.04	0.09
292	16	PDE4DIP	G239D	Met Only	0.00	0.10
293	16	PDE4DIP	R6H	Shared	0.03	0.05
294	16	MUC1	F274L	Met Only	0.00	0.19
295	16	PRCC	R482H	Shared	0.09	0.22
296	16	NTRK1	F385L	Met Only	0.00	0.17
297	16	FCGR2B	S177L	Met Only	0.00	0.14
298	16	TNN	K382T	Shared	0.09	0.16
299	16	CDC73	R139Q	Shared	0.11	0.16
300	16	CDC73	S345Y	Met Only	0.00	0.19
301	16	KDM5B	R1461C	Shared	0.12	0.16
302	16	MDM4	G216D	Met Only	0.00	0.18
303	16	RPS6KC1	T634N	Shared	0.07	0.16
304	16	RPS6KC1	F1056L	Shared	0.12	0.18
305	16	CENPF	K606N	Shared	0.08	0.18
306	16	CENPF	S876R	Shared	0.13	0.18
307	16	CENPF	A2104T	Met Only	0.00	0.21
308	16	FH	F248C	Shared	0.10	0.15
309	16	SDCCAG8	I6M	Shared	0.10	0.21
310	16	AKT3	K353Q	Primary Only	0.03	0.00
311	16	MLLT10	S406R	Shared	0.12	0.19
312	16	MLLT10	K466N	Shared	0.10	0.16
313	16	MLLT10	V938I	Shared	0.12	0.19
314	16	BMI1	R123I	Primary Only	0.07	0.00
315	16	MYO3A	R991W	Shared	0.08	0.19

316	16	MYO3A	A1314V	Shared	0.11	0.21
317	16	MAP3K8	E43X	Shared	0.10	0.17
318	16	MAP3K8	F143V	Shared	0.09	0.24
319	16	MAP3K8	L148I	Met Only	0.00	0.05
320	16	KIF5B	M740L	Primary Only	0.12	0.00
321	16	RET	K722N	Shared	0.07	0.16
322	16	RET	S774X	Met Only	0.00	0.18
323	16	ERCC6	E272K	Primary Only	0.11	0.00
324	16	NCOA4	S296Y	Met Only	0.00	0.18
325	16	NCOA4	K538N	Shared	0.07	0.19
326	16	SIRT1	D386Y	Met Only	0.00	0.03
327	16	SIRT1	E520X	Primary Only	0.09	0.00
328	16	SIRT1	R636Q	Met Only	0.00	0.19
329	16	TET1	M82I	Shared	0.13	0.15
330	16	TET1	K1712T	Shared	0.11	0.12
331	16	KAT6B	E204D	Met Only	0.00	0.16
332	16	TNKS2	E506K	Shared	0.10	0.20
333	16	TNKS2	D638G	Met Only	0.00	0.20
334	16	SLK	E227X	Met Only	0.00	0.19
335	16	SLK	K275N	Shared	0.10	0.23
336	16	SLK	S378N	Shared	0.10	0.19
337	16	SLK	E934D	Met Only	0.00	0.15
338	16	SLK	R1076H	Shared	0.12	0.20
339	16	ENSG00000227560	K44N	Met Only	0.00	0.21
340	16	PTPRE	R666Q	Met Only	0.00	0.15
341	16	NUP98	R1472X	Shared	0.10	0.17
342	16	NUP98	D1156N	Shared	0.06	0.18
343	16	NUP98	T341A	Shared	0.09	0.21
344	16	STK33	K512N	Shared	0.11	0.20
345	16	WEE1	S212Y	Met Only	0.00	0.18
346	16	WT1	E350D	Shared	0.10	0.17
347	16	PTPRJ	R340Q	Shared	0.09	0.18
348	16	PTPRJ	K1068N	Primary Only	0.12	0.00
349	16	CLP1	A165T	Shared	0.08	0.17
350	16	SDHAF2	L88F	Shared	0.11	0.15
351	16	SDHAF2	Y127C	Shared	0.13	0.18
352	16	MALAT1	I21M	Shared	0.15	0.19
353	16	MALAT1	K64N	Shared	0.09	0.21
354	16	CCND1	E273X	Met Only	0.00	0.33
355	16	NUMA1	D248G	Primary Only	0.04	0.00
356	16	RSF1	R851H	Met Only	0.00	0.05
357	16	RSF1	D533N	Shared	0.11	0.17
358	16	RSF1	K138N	Met Only	0.00	0.16
359	16	GAB2	S381Y	Primary Only	0.10	0.00
360	16	GAB2	F118L	Shared	0.08	0.19

361	16	GUCY1A2	I366L	Shared	0.09	0.19
362	16	ATM	L259I	Shared	0.11	0.17
363	16	ATM	S274Y	Shared	0.15	0.20
364	16	ATM	L948R	Primary Only	0.10	0.00
365	16	ATM	S978Y	Primary Only	0.09	0.00
366	16	ATM	S1357Y	Shared	0.10	0.17
367	16	ATM	L2001V	Met Only	0.00	0.20
368	16	ATM	R2380I	Shared	0.16	0.18
369	16	DDX10	D463Y	Met Only	0.00	0.15
370	16	DDX10	E544K	Shared	0.09	0.17
371	16	DDX10	K665N	Met Only	0.00	0.17
372	16	MLL	E891D	Met Only	0.00	0.18
373	16	MLL	K1598T	Primary Only	0.12	0.00
374	16	MLL	E1900X	Primary Only	0.09	0.00
375	16	MLL	S2969C	Shared	0.10	0.20
376	16	MLL	L3257S	Met Only	0.00	0.20
377	16	MLL	S3786Y	Shared	0.10	0.19
378	16	KDM5A	R1428Q	Shared	0.13	0.16
379	16	KDM5A	A1140T	Shared	0.09	0.16
380	16	KDM5A	E1114D	Primary Only	0.09	0.00
381	16	KDM5A	V610A	Shared	0.10	0.19
382	16	ERC1	N165H	Shared	0.13	0.19
383	16	LRRK2	Q689K	Shared	0.11	0.13
384	16	LRRK2	D861N	Shared	0.14	0.17
385	16	LRRK2	E915X	Shared	0.09	0.22
386	16	LRRK2	F916L	Shared	0.09	0.21
387	16	LRRK2	D1375Y	Shared	0.12	0.16
388	16	LRRK2	A1442T	Shared	0.13	0.15
389	16	LRRK2	E1578X	Shared	0.06	0.17
390	16	LRRK2	I1610M	Shared	0.11	0.12
391	16	LRRK2	E1665X	Shared	0.10	0.16
392	16	LRRK2	E1902X	Shared	0.10	0.15
393	16	ARID2	R294I	Shared	0.11	0.18
394	16	MLL2	S35Y	Met Only	0.00	0.16
395	16	MLL2	E1588K	Primary Only	0.12	0.00
396	16	MLL2	C1501W	Met Only	0.00	0.20
397	16	SMARCD1	R183Q	Shared	0.06	0.17
398	16	TARBP2	S110Y	Met Only	0.00	0.16
399	16	HOXC13	S322L	Met Only	0.00	0.30
400	16	NACA	P858H	Met Only	0.00	0.17
401	16	WIF1	H43R	Met Only	0.00	0.12
402	16	HMGA2	L70I	Primary Only	0.03	0.00
403	16	NAV3	L456I	Shared	0.11	0.19
404	16	NAV3	E469X	Met Only	0.00	0.16
405	16	NAV3	K1095T	Shared	0.11	0.21
406	16	NAV3	S2140P	Primary Only	0.13	0.00

407	16	APAF1	D385Y	Primary Only	0.09	0.00
408	16	SCYL2	N178K	Shared	0.10	0.20
409	16	SCYL2	R505C	Met Only	0.00	0.15
410	16	PTPN11	E123D	Shared	0.14	0.19
411	16	PTPN11	E313D	Shared	0.10	0.19
412	16	PTPN11	E400X	Shared	0.09	0.17
413	16	TBX5	D21Y	Met Only	0.00	0.15
414	16	CIT	E1878X	Shared	0.08	0.18
415	16	CIT	E98K	Shared	0.11	0.18
416	16	FZD10	E348K	Shared	0.11	0.16
417	16	EP400	E1151K	Met Only	0.00	0.20
418	16	EP400	R2293C	Primary Only	0.09	0.00
419	16	ZMYM2	L851I	Shared	0.09	0.21
420	16	ZMYM2	P917S	Met Only	0.00	0.19
421	16	ZMYM2	3’splice	Shared	0.20	0.17
422	16	LATS2	S32Y	Shared	0.17	0.20
423	16	FLT1	L879I	Primary Only	0.09	0.00
424	16	FLT1	F666L	Shared	0.15	0.17
425	16	FLT1	R339Q	Shared	0.09	0.21
426	16	FLT1	D231Y	Shared	0.18	0.16
427	16	BRCA2	I614S	Primary Only	0.07	0.00
428	16	BRCA2	F1449L	Shared	0.18	0.18
429	16	BRCA2	R1704I	Primary Only	0.08	0.00
430	16	BRCA2	S3338X	Shared	0.18	0.16
431	16	DCLK1	G393V	Primary Only	0.08	0.00
432	16	DCLK1	R60C	Shared	0.20	0.19
433	16	FOXO1	K423T	Shared	0.10	0.20
434	16	FOXO1	R316Q	Shared	0.08	0.18
435	16	FOXO1	R214C	Primary Only	0.07	0.00
436	16	LCP1	K592N	Shared	0.10	0.23
437	16	LCP1	S83I	Shared	0.19	0.19
438	16	RB1	S302Y	Shared	0.15	0.16
439	16	RB1	E746X	Primary Only	0.12	0.00
440	16	EDNRB	F28L	Shared	0.15	0.15
441	16	ERCC5	R71C	Shared	0.19	0.18
442	16	ERCC5	S323X	Shared	0.07	0.18
443	16	ERCC5	E975A	Met Only	0.00	0.16
444	16	ERCC5	P1122S	Shared	0.15	0.17
445	16	CCNB1IP1	E108X	Shared	0.06	0.21
446	16	CHD8	F2561L	Met Only	0.00	0.18
447	16	CHD8	S2469Y	Shared	0.09	0.18
448	16	CHD8	R2158C	Shared	0.10	0.16
449	16	CHD8	R773X	Shared	0.10	0.20
450	16	CHD8	F668L	Shared	0.10	0.16
451	16	SALL2	F287L	Shared	0.13	0.27
452	16	SALL2	R3W	Met Only	0.00	0.21

453	16	MAP4K5	S845R	Shared	0.11	0.19
454	16	NIN	L1735I	Shared	0.11	0.21
455	16	NIN	S887Y	Shared	0.08	0.19
456	16	NIN	F242L	Shared	0.14	0.16
457	16	KTN1	N527H	Primary Only	0.09	0.00
458	16	KTN1	E1124X	Shared	0.09	0.18
459	16	ARID4A	L1137R	Shared	0.09	0.19
460	16	ARID4A	E1191X	Shared	0.11	0.22
461	16	HIF1A	L117F	Shared	0.13	0.18
462	16	HIF1A	E465K	Met Only	0.00	0.19
463	16	HIF1A	K556E	Met Only	0.00	0.18
464	16	HIF1A	S621N	Shared	0.10	0.17
465	16	HIF1A	K649X	Shared	0.10	0.21
466	16	GPHN	E102X	Shared	0.11	0.20
467	16	TSHR	T159P	Shared	0.10	0.12
468	16	TRIP11	S1640X	Shared	0.10	0.17
469	16	TRIP11	S1376L	Shared	0.11	0.20
470	16	TRIP11	S932Y	Primary Only	0.10	0.00
471	16	TRIP11	N449S	Shared	0.12	0.20
472	16	TRIP11	L284I	Primary Only	0.09	0.00
473	16	GOLGA5	R653L	Met Only	0.00	0.20
474	16	DICER1	V1919I	Primary Only	0.04	0.00
475	16	DICER1	M1782L	Shared	0.08	0.18
476	16	DICER1	L1178I	Primary Only	0.10	0.00
477	16	DICER1	A54T	Primary Only	0.12	0.00
478	16	TCL1A	5’splice	Shared	0.14	0.12
479	16	EIF2AK4	R465C	Primary Only	0.12	0.00
480	16	EIF2AK4	S959L	Shared	0.12	0.22
481	16	EIF2AK4	R989W	Met Only	0.00	0.18
482	16	EIF2AK4	D1353Y	Met Only	0.00	0.16
483	16	BUB1B	R324C	Primary Only	0.09	0.00
484	16	MAPK6	N456H	Primary Only	0.11	0.00
485	16	MAPK6	R635M	Primary Only	0.08	0.00
486	16	TCF12	I542T	Met Only	0.00	0.19
487	16	MAP2K1	R96K	Shared	0.11	0.19
488	16	MAP2K5	A324V	Shared	0.09	0.20
489	16	PTPN9	R311H	Shared	0.12	0.21
490	16	ADAMTSL 3	R1567C	Met Only	0.00	0.17
491	16	NTRK3	L838I	Shared	0.11	0.15
492	16	NTRK3	E543X	Shared	0.07	0.19
493	16	NTRK3	A96T	Primary Only	0.07	0.00
494	16	BLM	R959X	Shared	0.10	0.20
495	16	FES	3’splice	Shared	0.13	0.14
496	16	FES	R100I	Met Only	0.00	0.18
497	16	FES	S175N	Shared	0.10	0.19

498	16	IGF1R	R504C	Shared	0.08	0.17
499	16	IGF1R	3’splice	Met Only	0.00	0.17
500	16	CREBBP	S2076L	Shared	0.17	0.23
501	16	CREBBP	T1274P	Shared	0.17	0.29
502	16	CREBBP	T1261K	Primary Only	0.09	0.00
503	16	RBFOX1	R6I	Met Only	0.00	0.14
504	16	RBFOX1	E33D	Met Only	0.00	0.13
505	16	RBFOX1	S134Y	Shared	0.18	0.27
506	16	SNX29	D178N	Shared	0.10	0.18
507	16	SNX29	E589K	Shared	0.09	0.17
508	16	SNX29	E688X	Shared	0.08	0.14
509	16	ERCC4	R292Q	Shared	0.14	0.31
510	16	ERCC4	E572X	Primary Only	0.03	0.00
511	16	MYH11	R1710H	Primary Only	0.09	0.00
512	16	MYH11	T1553M	Shared	0.17	0.45
513	16	ENSG00000263335	K41T	Shared	0.15	0.31
514	16	MYH11	R280C	Met Only	0.00	0.17
515	16	SMG1	K2476T	Shared	0.14	0.29
516	16	SMG1	V1521A	Shared	0.12	0.17
517	16	SMG1	A1508T	Met Only	0.00	0.16
518	16	SMG1	K1322N	Met Only	0.00	0.13
519	16	SMG1	E999K	Shared	0.08	0.16
520	16	SMG1	R846I	Met Only	0.00	0.14
521	16	SMG1	E840X	Met Only	0.00	0.16
522	16	SMG1	N712T	Shared	0.07	0.15
523	16	PALB2	L451V	Met Only	0.00	0.19
524	16	PALB2	D208A	Met Only	0.00	0.17
525	16	ERN2	D480Y	Primary Only	0.06	0.00
526	16	MAPK3	R278Q	Shared	0.23	0.24
527	16	CYLD	E9X	Met Only	0.00	0.16
528	16	CYLD	D893N	Met Only	0.00	0.16
529	16	MMP2	F534L	Shared	0.09	0.18
530	16	CDH11	L479F	Shared	0.05	0.16
531	16	CDH11	D428N	Primary Only	0.08	0.00
532	16	CDH11	N395H	Shared	0.09	0.14
533	16	CDH11	R118Q	Shared	0.07	0.16
534	16	CDH11	E3D	Met Only	0.00	0.09
535	16	CBFB	L178V	Shared	0.18	0.32
536	16	CDH1	K314N	Met Only	0.00	0.17
537	16	ADAMTS18	N710S	Shared	0.16	0.36
538	16	ADAMTS18	P66S	Shared	0.07	0.18
539	16	WWOX	D286N	Met Only	0.00	0.19
540	16	PLCG2	R104X	Shared	0.15	0.32
541	16	PLCG2	R956H	Shared	0.15	0.14

542	16	FANCA	K317E	Met Only	0.00	0.19
543	16	FANCA	3’splice	Met Only	0.00	0.15
544	16	FANCA	E169D	Shared	0.16	0.33
545	16	FANCA	G110D	Shared	0.09	0.16
546	16	CRK	F150L	Primary Only	0.07	0.00
547	16	MINK1	R419Q	Met Only	0.00	0.29
548	16	MINK1	D870N	Shared	0.11	0.19
549	16	USP6	R674I	Met Only	0.00	0.19
550	16	USP6	R686K	Shared	0.11	0.18
551	16	USP6	R1082Q	Shared	0.12	0.18
552	16	USP6	S1184R	Met Only	0.00	0.23
553	16	USP6	H1262Y	Met Only	0.00	0.18
554	16	RABEP1	E219X	Met Only	0.00	0.16
555	16	NLRP1	F1323L	Shared	0.11	0.17
556	16	NLRP1	R1312X	Shared	0.11	0.19
557	16	GAS7	E197D	Shared	0.11	0.23
558	16	MAP2K4	N43H	Shared	0.14	0.14
559	16	MAP2K4	R132I	Shared	0.13	0.21
560	16	MAP2K4	R134W	Shared	0.13	0.21
561	16	NCOR1	E727K	Shared	0.09	0.23
562	16	NCOR1	R295I	Met Only	0.00	0.20
563	16	NCOR1	S161L	Met Only	0.00	0.04
564	16	ULK2	F945L	Shared	0.16	0.17
565	16	SPECC1	E157D	Shared	0.09	0.22
566	16	SPECC1	D181Y	Shared	0.11	0.20
567	16	SPECC1	R188Q	Shared	0.10	0.21
568	16	SPECC1	K419N	Shared	0.09	0.20
569	16	MAP2K3	3’splice	Shared	0.05	0.08
570	16	NEK8	E68D	Met Only	0.00	0.18
571	16	TAOK1	D292Y	Shared	0.11	0.18
572	16	TAOK1	R605Q	Shared	0.14	0.18
573	16	NF1	N78H	Primary Only	0.12	0.00
574	16	NF1	K111T	Shared	0.13	0.19
575	16	NF1	E524X	Shared	0.10	0.14
576	16	NF1	A761T	Shared	0.13	0.21
577	16	NF1	R765H	Shared	0.13	0.19
578	16	NF1	E1458X	Shared	0.10	0.18
579	16	NF1	G1502S	Met Only	0.00	0.17
580	16	NF1	E2469X	Shared	0.11	0.20
581	16	NF1	L2639I	Shared	0.14	0.19
582	16	NF1	V2732A	Shared	0.09	0.19
583	16	SUZ12	R288C	Shared	0.10	0.15
584	16	SUZ12	L529I	Shared	0.12	0.27
585	16	TAF15	R187H	Shared	0.10	0.17
586	16	CDK12	L495V	Shared	0.10	0.19
587	16	CDK12	L926I	Shared	0.07	0.20

588	16	CDK12	R1067X	Shared	0.09	0.20
589	16	RARA	S16N	Shared	0.07	0.22
590	16	MAP3K14	L379I	Shared	0.15	0.23
591	16	MAP3K14	R21W	Shared	0.10	0.17
592	16	MAP3K14	R16W	Shared	0.11	0.19
593	16	CDC27	W768R	Shared	0.12	0.18
594	16	CDC27	N571I	Primary Only	0.04	0.00
595	16	CDC27	V560F	Primary Only	0.05	0.00
596	16	CDC27	V201F	Met Only	0.00	0.15
597	16	CDC27	D23Y	Primary Only	0.12	0.00
598	16	COL1A1	S889P	Met Only	0.00	0.19
599	16	MSI2	E72X	Met Only	0.00	0.18
600	16	MSI2	K11N	Shared	0.13	0.20
601	16	RNF43	S745F	Shared	0.10	0.21
602	16	TEX14	Q1369K	Shared	0.12	0.17
603	16	TEX14	I1174T	Shared	0.11	0.18
604	16	TEX14	D998Y	Primary Only	0.10	0.00
605	16	CLTC	F368V	Shared	0.11	0.18
606	16	CLTC	R1453C	Shared	0.12	0.20
607	16	BRIP1	Q820K	Met Only	0.00	0.16
608	16	BRIP1	F771L	Shared	0.09	0.19
609	16	BRIP1	T476P	Shared	0.07	0.18
610	16	BRIP1	E339X	Shared	0.08	0.22
611	16	BRIP1	R160I	Shared	0.10	0.18
612	16	MAP3K3	E3K	Shared	0.09	0.22
613	16	MAP3K3	V30F	Primary Only	0.06	0.00
614	16	ERN1	T55A	Met Only	0.00	0.21
615	16	DDX5	F505C	Shared	0.12	0.20
616	16	DDX5	K56N	Shared	0.09	0.27
617	16	DDX5	E39D	Met Only	0.00	0.25
618	16	PRKCA	5’splice	Met Only	0.00	0.17
619	16	BPTF	S718Y	Primary Only	0.08	0.00
620	16	BPTF	S95L	Shared	0.09	0.19
621	16	MAP2K6	R31Q	Shared	0.10	0.19
622	16	SEPT9	R29I	Met Only	0.00	0.16
623	16	LAMA1	R2084W	Shared	0.07	0.21
624	16	LAMA1	A1944V	Shared	0.15	0.21
625	16	LAMA1	D1473N	Met Only	0.00	0.16
626	16	LAMA1	S1339X	Shared	0.13	0.18
627	16	ROCK1	R1012Q	Shared	0.16	0.17
628	16	ROCK1	R500I	Met Only	0.00	0.18
629	16	ROCK1	E111X	Met Only	0.00	0.21
630	16	ZNF521	H1213N	Met Only	0.00	0.17
631	16	ZNF521	E1073K	Shared	0.07	0.18
632	16	ZNF521	S561Y	Shared	0.06	0.18
633	16	ZNF521	S58L	Shared	0.19	0.17

634	16	SS18	Y270D	Shared	0.10	0.20
635	16	SMAD4	G89X	Primary Only	0.17	0.00
636	16	SMAD4	G352E	Primary Only	0.08	0.00
637	16	MALT1	Q443H	Shared	0.10	0.18
638	16	KDSR	N262H	Primary Only	0.09	0.00
639	16	INSR	E860X	Shared	0.11	0.18
640	16	INSR	T731M	Shared	0.12	0.19
641	16	INSR	K80Q	Primary Only	0.12	0.00
642	16	TYK2	D898Y	Shared	0.10	0.17
643	16	KEAP1	A40T	Shared	0.17	0.20
644	16	DNM2	R657H	Shared	0.09	0.21
645	16	MAST1	R66Q	Met Only	0.00	0.21
646	16	TPM4	K118Q	Shared	0.07	0.21
647	16	TPM4	A155V	Shared	0.15	0.22
648	16	TPM4	E196X	Met Only	0.00	0.18
649	16	JAK3	A853S	Met Only	0.00	0.28
650	16	CCNE1	D261G	Shared	0.15	0.15
651	16	MAP4K1	A755D	Shared	0.12	0.22
652	16	MAP4K1	E391D	Met Only	0.00	0.21
653	16	MAP3K10	R886I	Met Only	0.00	0.17
654	16	AKT2	N352K	Met Only	0.00	0.25
655	16	AXL	K476N	Met Only	0.00	0.15
656	16	ERCC2	K401I	Primary Only	0.12	0.00
657	16	ERCC2	V50A	Shared	0.11	0.22
658	16	PPP2R1A	K33N	Met Only	0.00	0.22
659	16	ZNF331	S340L	Shared	0.10	0.20
660	16	PEG3	N1241D	Met Only	0.00	0.20
661	16	PEG3	E921K	Shared	0.09	0.19
662	16	PEG3	R889C	Primary Only	0.10	0.00
663	16	PEG3	R819C	Shared	0.10	0.17
664	16	ROCK2	R760I	Shared	0.08	0.18
665	16	ROCK2	E726K	Shared	0.23	0.28
666	16	ROCK2	E454K	Shared	0.10	0.13
667	16	ROCK2	D393N	Shared	0.18	0.28
668	16	ROCK2	E366X	Shared	0.18	0.28
669	16	ROCK2	S182R	Shared	0.21	0.34
670	16	MYCN	P57S	Shared	0.20	0.30
671	16	MYCN	L359F	Met Only	0.00	0.16
672	16	NCOA1	R252Q	Shared	0.09	0.16
673	16	DNMT3A	D712Y	Shared	0.18	0.31
674	16	DNMT3A	A644T	Met Only	0.00	0.11
675	16	DNMT3A	D600N	Shared	0.13	0.17
676	16	ALK	R401X	Shared	0.27	0.47
677	16	LTBP1	E807D	Shared	0.17	0.23
678	16	LTBP1	5’splice	Met Only	0.00	0.15
679	16	MAP4K3	L629X	Primary Only	0.09	0.00

680	16	MAP4K3	E61X	Shared	0.09	0.14
681	16	MSH2	I648L	Met Only	0.00	0.14
682	16	REL	G590D	Shared	0.20	0.30
683	16	XPO1	V600I	Met Only	0.00	0.18
684	16	XPO1	R340I	Met Only	0.00	0.19
685	16	ENSG00000231134	S24I	Shared	0.19	0.27
686	16	TCF7L1	S497L	Met Only	0.00	0.15
687	16	AFF3	R1102X	Shared	0.13	0.15
688	16	AFF3	S790Y	Shared	0.13	0.28
689	16	MAP4K4	I1107M	Shared	0.14	0.15
690	16	MERTK	L861V	Primary Only	0.10	0.00
691	16	GLI2	F218L	Primary Only	0.10	0.00
692	16	GLI2	R1548Q	Shared	0.16	0.18
693	16	MAP3K2	R417Q	Shared	0.12	0.15
694	16	MAP3K2	E381X	Shared	0.09	0.13
695	16	MAP3K2	K116N	Shared	0.12	0.18
696	16	MAP3K2	A20V	Shared	0.14	0.29
697	16	BAZ2B	I1888M	Shared	0.09	0.15
698	16	BAZ2B	F1851C	Shared	0.11	0.18
699	16	BAZ2B	L1389I	Shared	0.09	0.17
700	16	XIRP2	F149V	Shared	0.13	0.33
701	16	XIRP2	L198I	Shared	0.19	0.30
702	16	XIRP2	3’splice	Shared	0.09	0.22
703	16	XIRP2	S268Y	Shared	0.07	0.13
704	16	XIRP2	R493I	Primary Only	0.09	0.00
705	16	XIRP2	S861Y	Shared	0.12	0.18
706	16	XIRP2	L995R	Met Only	0.00	0.15
707	16	XIRP2	S1403A	Primary Only	0.10	0.00
708	16	XIRP2	E1599X	Primary Only	0.10	0.00
709	16	XIRP2	I1608T	Shared	0.08	0.16
710	16	XIRP2	L1694I	Shared	0.08	0.14
711	16	XIRP2	P2144L	Shared	0.09	0.15
712	16	XIRP2	S2407Y	Shared	0.10	0.16
713	16	XIRP2	E2557G	Shared	0.18	0.30
714	16	XIRP2	F2947L	Met Only	0.00	0.16
715	16	XIRP2	S2959L	Shared	0.20	0.33
716	16	XIRP2	F3425C	Shared	0.08	0.17
717	16	XIRP2	N741K	Met Only	0.00	0.17
718	16	MYO3B	5’splice	Shared	0.17	0.28
719	16	CHN1	5’splice	Shared	0.17	0.33
720	16	CHN1	N140T	Met Only	0.00	0.16
721	16	PMS1	E45D	Shared	0.08	0.11
722	16	PMS1	N506S	Primary Only	0.10	0.00
723	16	PMS1	K549T	Met Only	0.00	0.16
724	16	MYO1B	R721H	Met Only	0.00	0.18

725	16	MYO1B	T873M	Shared	0.21	0.35
726	16	SF3B1	R957Q	Primary Only	0.07	0.00
727	16	SF3B1	R425Q	Shared	0.09	0.13
728	16	SF3B1	D68N	Shared	0.07	0.13
729	16	CASP8	L176F	Primary Only	0.09	0.00
730	16	CASP8	E441X	Shared	0.22	0.31
731	16	IDH1	F354L	Primary Only	0.07	0.00
732	16	ERBB4	E874X	Met Only	0.00	0.15
733	16	ERBB4	R26K	Primary Only	0.09	0.00
734	16	ERBB4	Q151H	Primary Only	0.09	0.00
735	16	FN1	5’splice	Met Only	0.00	0.17
736	16	FN1	R784Q	Shared	0.09	0.13
737	16	FN1	K648N	Primary Only	0.11	0.00
738	16	STK36	K214N	Primary Only	0.08	0.00
739	16	EPHA4	A912D	Met Only	0.00	0.17
740	16	EPHA4	V173A	Met Only	0.00	0.11
741	16	EPHA4	E42X	Met Only	0.00	0.15
742	16	PAX3	D28N	Shared	0.09	0.15
743	16	TGM3	E693K	Primary Only	0.06	0.00
744	16	PTPRA	R345I	Shared	0.20	0.30
745	16	MYLK2	E236D	Met Only	0.00	0.19
746	16	ASXL1	E1228X	Shared	0.18	0.30
747	16	TOP1	K451T	Primary Only	0.09	0.00
748	16	TOP1	E641D	Shared	0.17	0.31
749	16	CHD6	S879Y	Shared	0.08	0.15
750	16	CHD6	S428Y	Met Only	0.00	0.15
751	16	PTPRT	K1428T	Met Only	0.00	0.33
752	16	PTPRT	Q900H	Primary Only	0.08	0.00
753	16	PTPRT	R835C	Shared	0.26	0.35
754	16	PTPRT	S824P	Shared	0.14	0.29
755	16	PTPRT	D597N	Met Only	0.00	0.16
756	16	SDC4	E102K	Shared	0.11	0.17
757	16	PREX1	E888K	Met Only	0.00	0.11
758	16	PREX1	D614N	Shared	0.08	0.15
759	16	PREX1	E104K	Shared	0.09	0.14
760	16	ZNF217	E665D	Shared	0.08	0.15
761	16	ZNF217	D528N	Shared	0.08	0.18
762	16	AURKA	E6X	Primary Only	0.08	0.00
763	16	GNAS	A56T	Shared	0.17	0.33
764	16	GNAS	R217C	Met Only	0.00	0.15
765	16	GNAS	E651X	Met Only	0.00	0.15
766	16	GNAS	E717K	Met Only	0.00	0.13
767	16	TIAM1	F1511L	Met Only	0.00	0.21
768	16	TIAM1	K1140N	Met Only	0.00	0.18
769	16	TIAM1	R871X	Shared	0.09	0.18
770	16	TIAM1	R620H	Met Only	0.00	0.19

771	16	TIAM1	D84Y	Met Only	0.00	0.33
772	16	TIAM1	R23H	Shared	0.14	0.22
773	16	TMPRSS2	N230T	Shared	0.07	0.19
774	16	CLTCL1	S1544L	Shared	0.09	0.14
775	16	CLTCL1	F1410L	Shared	0.09	0.22
776	16	CLTCL1	A378V	Shared	0.13	0.19
777	16	CLTCL1	M295I	Shared	0.06	0.19
778	16	BCR	K739R	Met Only	0.00	0.17
779	16	SMARCB1	R377C	Shared	0.09	0.16
780	16	MN1	A1102T	Shared	0.14	0.26
781	16	CHEK2	E364X	Met Only	0.00	0.18
782	16	ZNRF3	Q464H	Met Only	0.00	0.19
783	16	MYH9	R1933Q	Shared	0.15	0.22
784	16	MYH9	R1839Q	Shared	0.11	0.19
785	16	MYH9	E1820D	Shared	0.14	0.23
786	16	MYH9	E1337D	Shared	0.13	0.21
787	16	MKL1	E783K	Shared	0.09	0.16
788	16	MKL1	R14I	Primary Only	0.08	0.00
789	16	EP300	R86Q	Shared	0.10	0.18
790	16	XRCC6	E22D	Shared	0.13	0.17
791	16	SRGAP3	D400N	Met Only	0.00	0.16
792	16	FANCD2	3’splice	Shared	0.12	0.16
793	16	FANCD2	K913N	Met Only	0.00	0.14
794	16	FANCD2	H1348N	Primary Only	0.04	0.00
795	16	RAF1	R292Q	Shared	0.11	0.15
796	16	XPC	E701X	Shared	0.12	0.20
797	16	TOP2B	D1170Y	Shared	0.15	0.21
798	16	TOP2B	E1156D	Met Only	0.00	0.13
799	16	NEK10	N86H	Primary Only	0.10	0.00
800	16	MLH1	K254N	Met Only	0.00	0.19
801	16	MLH1	L397M	Shared	0.10	0.19
802	16	SCN5A	K1504N	Shared	0.08	0.18
803	16	SNRK	D99Y	Primary Only	0.09	0.00
804	16	SETD2	E2477X	Shared	0.10	0.18
805	16	SETD2	R2077Q	Met Only	0.00	0.20
806	16	SETD2	R1598Q	Shared	0.10	0.19
807	16	SETD2	K1386N	Met Only	0.00	0.22
808	16	SETD2	E1234K	Shared	0.11	0.17
809	16	SETD2	E670X	Primary Only	0.09	0.00
810	16	SETD2	E639K	Shared	0.10	0.15
811	16	SETD2	R529I	Shared	0.09	0.15
812	16	SETD2	E517X	Shared	0.07	0.17
813	16	SMARCC1	R912X	Shared	0.09	0.16
814	16	SMARCC1	R297C	Shared	0.11	0.16
815	16	DNAH1	R3265H	Met Only	0.00	0.18
816	16	STAB1	K1474N	Shared	0.12	0.16

817	16	STAB1	V2476A	Met Only	0.00	0.24
818	16	STAB1	R2502X	Shared	0.12	0.18
819	16	STAB1	D2552N	Shared	0.10	0.20
820	16	PBRM1	F1487C	Shared	0.12	0.21
821	16	PBRM1	R876C	Shared	0.09	0.22
822	16	PBRM1	G318D	Met Only	0.00	0.18
823	16	PBRM1	E291X	Met Only	0.00	0.23
824	16	WNT5A	R260C	Shared	0.08	0.18
825	16	WNT5A	K121T	Primary Only	0.11	0.00
826	16	MITF	K22T	Shared	0.09	0.16
827	16	MITF	K28N	Shared	0.08	0.17
828	16	MITF	E367X	Shared	0.09	0.16
829	16	FOXP1	S627Y	Met Only	0.00	0.18
830	16	FOXP1	F561S	Met Only	0.00	0.18
831	16	FOXP1	F530S	Shared	0.10	0.18
832	16	ROBO2	L929I	Shared	0.10	0.21
833	16	EPHA3	R274Q	Shared	0.14	0.20
834	16	EPHA3	R330I	Met Only	0.00	0.22
835	16	EPHA6	A51V	Primary Only	0.11	0.00
836	16	EPHA6	V54M	Shared	0.11	0.24
837	16	EPHA6	F82L	Shared	0.07	0.19
838	16	EPHA6	K235T	Shared	0.12	0.19
839	16	CBLB	R468Q	Shared	0.11	0.20
840	16	CBLB	R172H	Met Only	0.00	0.17
841	16	GSK3B	E53X	Met Only	0.00	0.20
842	16	RPN1	S222N	Met Only	0.00	0.20
843	16	PIK3R4	R1040Q	Shared	0.07	0.18
844	16	PIK3R4	V566A	Primary Only	0.11	0.00
845	16	PIK3R4	V156A	Primary Only	0.08	0.00
846	16	NEK11	K162N	Met Only	0.00	0.14
847	16	NEK11	S597N	Shared	0.10	0.17
848	16	EPHB1	E605K	Shared	0.12	0.18
849	16	PIK3CB	5’splice	Shared	0.14	0.17
850	16	PIK3CB	V353A	Shared	0.05	0.18
851	16	ATR	K1313T	Shared	0.09	0.18
852	16	ATR	R1201H	Shared	0.09	0.16
853	16	ATR	5’splice	Shared	0.13	0.12
854	16	WWTR1	A389V	Shared	0.06	0.14
855	16	WWTR1	L189I	Met Only	0.00	0.17
856	16	GMPS	R322I	Shared	0.12	0.20
857	16	GMPS	R677Q	Shared	0.15	0.19
858	16	MLF1	E4X	Met Only	0.00	0.19
859	16	MLF1	5’splice	Shared	0.07	0.19
860	16	MLF1	E261X	Shared	0.07	0.15
861	16	MECOM	S1051Y	Met Only	0.00	0.18
862	16	MECOM	E1015D	Shared	0.12	0.15

863	16	MECOM	R846C	Shared	0.10	0.18
864	16	MECOM	S237X	Met Only	0.00	0.14
865	16	MECOM	K13N	Met Only	0.00	0.29
866	16	PIK3CA	R19I	Shared	0.11	0.19
867	16	MAP3K13	D31Y	Met Only	0.00	0.19
868	16	BCL6	E605K	Shared	0.08	0.23
869	16	SENP5	G45E	Shared	0.10	0.17
870	16	SENP5	R242W	Met Only	0.00	0.23
871	16	SENP5	T651A	Met Only	0.00	0.18
872	16	GAK	P631S	Shared	0.09	0.18
873	16	GAK	F192S	Shared	0.13	0.15
874	16	WHSC1	R602Q	Met Only	0.00	0.18
875	16	EVC2	E926K	Shared	0.10	0.15
876	16	EVC2	L437I	Shared	0.10	0.19
877	16	EVC2	E384D	Met Only	0.00	0.16
878	16	N4BP2	D127Y	Met Only	0.00	0.17
879	16	N4BP2	L286V	Met Only	0.00	0.23
880	16	N4BP2	L646V	Met Only	0.00	0.19
881	16	N4BP2	H969N	Shared	0.19	0.40
882	16	N4BP2	S1038Y	Shared	0.09	0.19
883	16	N4BP2	P1534T	Shared	0.08	0.19
884	16	FIP1L1	D84Y	Shared	0.08	0.18
885	16	KDR	V1318M	Shared	0.08	0.20
886	16	KDR	R932I	Primary Only	0.13	0.00
887	16	KDR	R819Q	Shared	0.08	0.18
888	16	KDR	N495T	Shared	0.10	0.21
889	16	KDR	Q37H	Shared	0.13	0.20
890	16	EPHA5	R919X	Shared	0.09	0.19
891	16	MAPK10	K404N	Shared	0.10	0.22
892	16	AFF1	E1051D	Shared	0.13	0.17
893	16	BMPR1B	E348X	Met Only	0.00	0.19
894	16	BMPR1B	R419I	Shared	0.08	0.15
895	16	RAP1GDS1	L5F	Shared	0.14	0.19
896	16	TET2	K58R	Met Only	0.00	0.18
897	16	TET2	K110N	Primary Only	0.09	0.00
898	16	TET2	N202S	Shared	0.10	0.19
899	16	FAT4	E120K	Shared	0.13	0.17
900	16	FAT4	E1154X	Met Only	0.00	0.17
901	16	FAT4	D1205Y	Primary Only	0.08	0.00
902	16	FAT4	E1751G	Met Only	0.00	0.17
903	16	FAT4	S2341F	Shared	0.10	0.15
904	16	FAT4	I2806M	Met Only	0.00	0.18
905	16	FAT4	D2813N	Shared	0.11	0.18
906	16	FAT4	D3012Y	Met Only	0.00	0.18
907	16	FAT4	V3373I	Shared	0.11	0.15
908	16	FAT4	G4550V	Shared	0.12	0.19

909	16	FAT4	S4709F	Shared	0.08	0.19
910	16	FBXW7	K652T	Shared	0.11	0.19
911	16	FBXW7	I435V	Met Only	0.00	0.21
912	16	FBXW7	E255X	Shared	0.12	0.16
913	16	FAT1	G4222E	Met Only	0.00	0.21
914	16	FAT1	D3222G	Met Only	0.00	0.21
915	16	FAT1	R2597X	Shared	0.08	0.18
916	16	FAT1	D2309N	Met Only	0.00	0.03
917	16	CTNND2	A750V	Shared	0.14	0.31
918	16	TRIO	Q576H	Primary Only	0.06	0.00
919	16	TRIO	K667N	Shared	0.18	0.33
920	16	TRIO	R924H	Met Only	0.00	0.12
921	16	TRIO	F1070V	Shared	0.17	0.34
922	16	TRIO	S1249Y	Primary Only	0.08	0.00
923	16	TRIO	F2807V	Met Only	0.00	0.20
924	16	TRIO	A2492S	Shared	0.20	0.28
925	16	IL7R	A58V	Shared	0.19	0.31
926	16	IL7R	E90X	Shared	0.14	0.27
927	16	IL7R	E90G	Met Only	0.00	0.14
928	16	IL7R	R291I	Shared	0.09	0.18
929	16	IL7R	E334K	Shared	0.09	0.14
930	16	LIFR	E687X	Met Only	0.00	0.18
931	16	LIFR	R510C	Shared	0.14	0.32
932	16	LIFR	S250Y	Shared	0.10	0.13
933	16	LIFR	N143T	Shared	0.16	0.30
934	16	IL6ST	E886X	Met Only	0.00	0.17
935	16	MAP3K1	F884L	Primary Only	0.10	0.00
936	16	MAP3K1	R1482X	Primary Only	0.09	0.00
937	16	MIER3	D299Y	Shared	0.08	0.19
938	16	MIER3		Shared	0.16	0.33
939	16	PLK2	F363L	Shared	0.11	0.31
940	16	MAST4	5’splice	Shared	0.17	0.34
941	16	MAST4	5’splice	Shared	0.10	0.15
942	16	MAST4	E1209D	Shared	0.20	0.30
943	16	MAST4	T1235P	Primary Only	0.10	0.00
944	16	PIK3R1	R348X	Shared	0.09	0.16
945	16	PIK3R1	N606H	Shared	0.11	0.14
946	16	PIK3R1	L647R	Shared	0.20	0.32
947	16	MSH3	D143N	Shared	0.12	0.12
948	16	MSH3	D320N	Shared	0.09	0.13
949	16	MSH3	R454X	Shared	0.11	0.14
950	16	MSH3	I590M	Met Only	0.00	0.17
951	16	MSH3	R734X	Shared	0.17	0.30
952	16	MSH3	S1134Y	Met Only	0.00	0.15
953	16	ACSL6	E256K	Shared	0.08	0.17
954	16	RAD50	K256Q	Met Only	0.00	0.16

955	16	RAD50	E257X	Shared	0.12	0.12
956	16	RAD50	E1084X	Met Only	0.00	0.17
957	16	RAD50	I1299S	Primary Only	0.04	0.00
958	16	BRD8	S877Y	Met Only	0.00	0.22
959	16	BRD8	F811L	Primary Only	0.03	0.00
960	16	CTNNA1	A227G	Met Only	0.00	0.14
961	16	CTNNA1	R360I	Primary Only	0.08	0.00
962	16	PCDHA13	R646H	Shared	0.16	0.14
963	16	PCDHA13	A731T	Shared	0.16	0.35
964	16	ARHGAP26	E137D	Met Only	0.00	0.17
965	16	ARHGAP26	E157X	Shared	0.18	0.32
966	16	ARHGAP26	K566Q	Shared	0.16	0.30
967	16	TCERG1	5’splice	Shared	0.07	0.18
968	16	TCERG1	S810N	Shared	0.09	0.16
969	16	TCERG1	S143Y	Primary Only	0.03	0.00
970	16	CSF1R	K706N	Shared	0.15	0.17
971	16	CSF1R	A14S	Shared	0.23	0.26
972	16	EBF1	D406N	Met Only	0.00	0.21
973	16	EBF1	F287L	Shared	0.09	0.17
974	16	NPM1	G224E	Shared	0.16	0.28
975	16	NSD1	S828Y	Met Only	0.00	0.18
976	16	MAPK9	S388Y	Shared	0.08	0.13
977	16	MAPK9	E273X	Met Only	0.00	0.14
978	16	FLT4	T195M	Shared	0.09	0.17
979	16	DEK	F117L	Met Only	0.00	0.17
980	16	TRIM27	E303D	Met Only	0.00	0.17
981	16	DDR1	E316K	Shared	0.21	0.23
982	16	RGL2	D206A	Shared	0.12	0.15
983	16	DAXX	K662N	Shared	0.09	0.19
984	16	DAXX	E602D	Shared	0.08	0.23
985	16	DAXX	R111K	Shared	0.13	0.19
986	16	DAXX	E482K	Shared	0.16	0.18
987	16	DAXX	E374X	Shared	0.10	0.18
988	16	SRSF3	E162X	Met Only	0.00	0.23
989	16	HSP90AB1	3’splice	Shared	0.12	0.15
990	16	HSP90AB1	K72T	Met Only	0.00	0.23
991	16	HSP90AB1	I580L	Shared	0.10	0.16
992	16	BAI3	T58M	Shared	0.10	0.21
993	16	BAI3	E146K	Primary Only	0.03	0.00
994	16	BAI3	E476X	Shared	0.10	0.16
995	16	BAI3	S563R	Shared	0.12	0.20
996	16	BAI3	D737Y	Shared	0.10	0.13
997	16	BAI3	S1458I	Primary Only	0.10	0.00
998	16	TTK	R414Q	Shared	0.14	0.24
999	16	TTK	S677R	Shared	0.09	0.20
1000	16	EPHA7	G699W	Shared	0.11	0.20

1001	16	EPHA7	5’splice	Shared	0.06	0.21
1002	16	EPHA7	D37N	Shared	0.10	0.20
1003	16	PRDM1	E96X	Shared	0.09	0.22
1004	16	PRDM1	I759N	Shared	0.12	0.21
1005	16	ROS1	T2266M	Shared	0.14	0.18
1006	16	ROS1	E2027K	Shared	0.07	0.18
1007	16	ROS1	F1082V	Primary Only	0.08	0.00
1008	16	ROS1	R245I	Shared	0.09	0.15
1009	16	RSPO3	R32I	Met Only	0.00	0.17
1010	16	LAMA2	E1303D	Shared	0.07	0.20
1011	16	LAMA2	3’splice	Shared	0.09	0.17
1012	16	LAMA2	S2373L	Shared	0.09	0.22
1013	16	LAMA2	E2796K	Shared	0.06	0.23
1014	16	MAP7	T364A	Primary Only	0.09	0.00
1015	16	MAP7	E107X	Met Only	0.00	0.21
1016	16	TNFAIP3	H39N	Shared	0.09	0.16
1017	16	ECT2L	M680I	Met Only	0.00	0.18
1018	16	ECT2L	S801C	Shared	0.11	0.19
1019	16	ARID1B	S920Y	Primary Only	0.04	0.00
1020	16	FNDC1	D100N	Shared	0.13	0.22
1021	16	FNDC1	K474E	Shared	0.08	0.19
1022	16	FNDC1	T1443N	Shared	0.14	0.20
1023	16	FNDC1	5’splice	Shared	0.09	0.18
1024	16	MAP3K4	L841F	Shared	0.12	0.18
1025	16	MAP3K4	R1404W	Shared	0.09	0.20
1026	16	MAP3K4	G1434E	Primary Only	0.04	0.00
1027	16	PARK2	R365X	Shared	0.11	0.21
1028	16	PARK2	R156I	Shared	0.09	0.20
1029	16	CARD11	L1132F	Shared	0.07	0.17
1030	16	CARD11	S480L	Primary Only	0.09	0.00
1031	16	SDK1	K182N	Shared	0.09	0.15
1032	16	SDK1	R1147X	Met Only	0.00	0.16
1033	16	SDK1	V1228F	Shared	0.06	0.15
1034	16	SDK1	R1517X	Shared	0.20	0.35
1035	16	PMS2	R427C	Shared	0.22	0.30
1036	16	PMS2	I194L	Met Only	0.00	0.17
1037	16	HOXA13	E336X	Shared	0.11	0.18
1038	16	GLI3	D301E	Met Only	0.00	0.13
1039	16	GLI3	R290Q	Shared	0.17	0.29
1040	16	IKZF1	S114L	Shared	0.17	0.31
1041	16	IKZF1	L241I	Met Only	0.00	0.14
1042	16	EGFR	V148M	Shared	0.10	0.18
1043	16	EGFR	R252H	Primary Only	0.09	0.00
1044	16	EGFR	E967K	Met Only	0.00	0.17
1045	16	HIP1	L196I	Shared	0.15	0.31
1046	16	PTPN12	K141N	Shared	0.19	0.29

1047	16	AKAP9	R21X	Shared	0.14	0.17
1048	16	AKAP9	E80D	Primary Only	0.10	0.00
1049	16	AKAP9	R407I	Met Only	0.00	0.17
1050	16	AKAP9	E753X	Shared	0.11	0.16
1051	16	AKAP9	L1270P	Primary Only	0.10	0.00
1052	16	AKAP9	E1332X	Shared	0.22	0.31
1053	16	AKAP9	E2630X	Shared	0.08	0.14
1054	16	AKAP9	L2798I	Primary Only	0.10	0.00
1055	16	AKAP9	F197L	Primary Only	0.09	0.00
1056	16	AKAP9	R3435X	Shared	0.18	0.29
1057	16	AKAP9	R3712X	Shared	0.20	0.16
1058	16	TRRAP	E649X	Met Only	0.00	0.17
1059	16	TRRAP	S3020R	Met Only	0.00	0.15
1060	16	TRRAP	S3439Y	Shared	0.18	0.33
1061	16	MET	V322A	Shared	0.19	0.30
1062	16	MET	V427A	Shared	0.09	0.14
1063	16	MET	L1213I	Met Only	0.00	0.05
1064	16	WNT2	K133T	Shared	0.19	0.32
1065	16	GRM8	F589L	Met Only	0.00	0.15
1066	16	GRM8	D127Y	Shared	0.20	0.33
1067	16	SMO	R763Q	Met Only	0.00	0.22
1068	16	CREB3L2	A276T	Met Only	0.00	0.18
1069	16	TRIM24	V156I	Met Only	0.00	0.16
1070	16	TRIM24	S422Y	Shared	0.25	0.34
1071	16	KIAA1549	F471L	Primary Only	0.07	0.00
1072	16	EZH2	E747X	Shared	0.18	0.32
1073	16	EZH2	E745K	Shared	0.19	0.31
1074	16	EZH2	K400E	Shared	0.09	0.19
1075	16	EZH2	E169D	Shared	0.11	0.19
1076	16	EZH2	E59X	Met Only	0.00	0.15
1077	16	MLL3	L4658X	Primary Only	0.07	0.00
1078	16	MLL3	5’splice	Shared	0.07	0.16
1079	16	MLL3	R3995X	Shared	0.16	0.30
1080	16	MLL3	F3689L	Met Only	0.00	0.15
1081	16	MLL3	Q3040H	Primary Only	0.09	0.00
1082	16	MLL3	L2918F	Met Only	0.00	0.18
1083	16	MLL3	S1824X	Met Only	0.00	0.12
1084	16	MLL3	R1092Q	Met Only	0.00	0.15
1085	16	MLL3	S881C	Met Only	0.00	0.05
1086	16	MLL3	K822N	Met Only	0.00	0.05
1087	16	MLL3	P743L	Met Only	0.00	0.05
1088	16	MLL3	F357L	Met Only	0.00	0.03
1089	16	MLL3	3’splice	Primary Only	0.08	0.00
1090	16	CSMD1	A3309P	Primary Only	0.09	0.00
1091	16	CSMD1	G3145X	Shared	0.13	0.16
1092	16	CSMD1	G1378C	Met Only	0.00	0.20

1093	16	CSMD1	S720Y	Primary Only	0.10	0.00
1094	16	CSMD1	R621Q	Met Only	0.00	0.15
1095	16	CSMD1	D401Y	Met Only	0.00	0.18
1096	16	DLC1	N1050S	Shared	0.10	0.19
1097	16	DLC1	F257L	Shared	0.13	0.22
1098	16	DLC1	L98I	Shared	0.06	0.16
1099	16	PCM1	E1950D	Shared	0.13	0.15
1100	16	PCM1	E2015X	Shared	0.10	0.17
1101	16	FZD3	R401X	Shared	0.14	0.18
1102	16	WRN	R389I	Shared	0.08	0.19
1103	16	WRN	D456Y	Shared	0.06	0.17
1104	16	WRN	G797D	Shared	0.11	0.20
1105	16	WRN	K1419N	Shared	0.11	0.20
1106	16	GPR124	A526T	Shared	0.21	0.29
1107	16	WHSC1L1	S910L	Met Only	0.00	0.18
1108	16	WHSC1L1	F3L	Shared	0.10	0.21
1109	16	ADAM9	N742H	Met Only	0.00	0.06
1110	16	KAT6A	E1408K	Met Only	0.00	0.16
1111	16	KAT6A	D1075Y	Met Only	0.00	0.17
1112	16	IKBKB	E192X	Met Only	0.00	0.21
1113	16	HOOK3	E401X	Shared	0.13	0.20
1114	16	HOOK3	K503N	Primary Only	0.08	0.00
1115	16	PRKDC	R2310I	Primary Only	0.09	0.00
1116	16	PRKDC	N1173T	Shared	0.10	0.15
1117	16	LYN	R198Q	Shared	0.10	0.17
1118	16	PLAG1	S370X	Primary Only	0.09	0.00
1119	16	CHCHD7	F28C	Shared	0.06	0.20
1120	16	SGK3	R76I	Shared	0.10	0.22
1121	16	PREX2	S59X	Primary Only	0.07	0.00
1122	16	PREX2	E86X	Shared	0.12	0.19
1123	16	PREX2	R463C	Shared	0.10	0.18
1124	16	PREX2	R484I	Met Only	0.00	0.17
1125	16	PREX2	I953L	Met Only	0.00	0.17
1126	16	NCOA2	N931H	Shared	0.06	0.34
1127	16	HEY1	E304D	Primary Only	0.08	0.00
1128	16	NBN	K715N	Shared	0.11	0.20
1129	16	NBN	E636X	Shared	0.12	0.17
1130	16	NBN	E62X	Shared	0.13	0.18
1131	16	RUNX1T1	L11F	Shared	0.09	0.21
1132	16	RUNX1T1	A410T	Shared	0.14	0.16
1133	16	RUNX1T1	5’splice	Shared	0.12	0.20
1134	16	RAD54B	K613Q	Shared	0.10	0.22
1135	16	RAD54B	E533X	Met Only	0.00	0.21
1136	16	STK3	D281N	Shared	0.12	0.12
1137	16	STK3	Q104L	Met Only	0.00	0.16
1138	16	STK3	E98X	Primary Only	0.11	0.00

1139	16	UBR5	E2228X	Shared	0.07	0.20
1140	16	UBR5	E807D	Shared	0.15	0.16
1141	16	RSPO2	S45F	Shared	0.10	0.19
1142	16	CSMD3	S3470Y	Shared	0.09	0.17
1143	16	CSMD3	R3329I	Shared	0.09	0.20
1144	16	CSMD3	E3303X	Shared	0.15	0.20
1145	16	CSMD3	W2863R	Met Only	0.00	0.19
1146	16	CSMD3	E2405D	Met Only	0.00	0.18
1147	16	CSMD3	G2061E	Primary Only	0.12	0.00
1148	16	CSMD3	5’splice	Primary Only	0.04	0.00
1149	16	CSMD3	5’splice	Shared	0.11	0.17
1150	16	CSMD3	T147A	Shared	0.09	0.22
1151	16	CSMD3	E115X	Shared	0.08	0.17
1152	16	MYC	F115L	Met Only	0.00	0.20
1153	16	NDRG1	D218Y	Shared	0.09	0.21
1154	16	RECQL4	V990A	Shared	0.10	0.11
1155	16	RECQL4	L191X	Shared	0.09	0.18
1156	16	SMARCA2	R384Q	Met Only	0.00	0.18
1157	16	SMARCA2	A1064V	Met Only	0.00	0.18
1158	16	JAK2	N981H	Shared	0.13	0.17
1159	16	JAK2	Q1112X	Primary Only	0.09	0.00
1160	16	CD274	5’splice	Shared	0.10	0.16
1161	16	PDCD1LG2	Q13P	Shared	0.10	0.18
1162	16	PTPRD	S431L	Primary Only	0.10	0.00
1163	16	PTPRD	D281Y	Shared	0.09	0.15
1164	16	NFIB	A73T	Shared	0.09	0.16
1165	16	PSIP1	R405X	Shared	0.12	0.22
1166	16	PSIP1	K73T	Met Only	0.00	0.13
1167	16	MLLT3	R441Q	Shared	0.10	0.19
1168	16	MLLT3	E75X	Met Only	0.00	0.18
1169	16	TEK	K365T	Shared	0.09	0.20
1170	16	TEK	S726Y	Shared	0.11	0.18
1171	16	MELK	E136G	Shared	0.10	0.20
1172	16	GNAQ	L271M	Met Only	0.00	0.18
1173	16	GNAQ	R149X	Shared	0.11	0.15
1174	16	OMD	D254N	Shared	0.10	0.19
1175	16	WNK2	K2135N	Met Only	0.00	0.13
1176	16	FANCC	A448V	Shared	0.11	0.19
1177	16	PTCH1	E1273K	Met Only	0.00	0.12
1178	16	PTCH1	L818V	Primary Only	0.09	0.00
1179	16	PTCH1	R607K	Shared	0.07	0.17
1180	16	PTCH1	R530I	Shared	0.10	0.21
1181	16	NR4A3	A436T	Met Only	0.00	0.18
1182	16	ABCA1	D357Y	Shared	0.07	0.19
1183	16	CNTRL	S222Y	Primary Only	0.09	0.00
1184	16	CNTRL	F365C	Shared	0.11	0.18

1185	16	CNTRL	R452Q	Shared	0.09	0.19
1186	16	CNTRL	E461X	Shared	0.11	0.20
1187	16	CNTRL	R711W	Shared	0.06	0.19
1188	16	CNTRL	R1058X	Met Only	0.00	0.16
1189	16	CNTRL	L1519V	Shared	0.12	0.19
1190	16	FNBP1	V418A	Shared	0.11	0.11
1191	16	ABL1	R153C	Shared	0.14	0.21
1192	16	TSC1	R1097C	Shared	0.10	0.21
1193	16	TSC1	D693Y	Met Only	0.00	0.20
1194	16	RPS6KA3	5’splice	Met Only	0.00	0.39
1195	16	BCOR	E1199X	Shared	0.22	0.34
1196	16	CASK	E92K	Shared	0.17	0.38
1197	16	KDM6A	S1192Y	Shared	0.21	0.40
1198	16	WAS	R86H	Shared	0.20	0.41
1199	16	GATA1	R271I	Shared	0.27	0.37
1200	16	KDM5C	E39D	Primary Only	0.20	0.00
1201	16	HUWE1	N2511T	Met Only	0.00	0.38
1202	16	ERCC6L	K742N	Primary Only	0.04	0.00
1203	16	ATRX	V2021I	Shared	0.31	0.32
1204	16	ATRX	E285D	Shared	0.19	0.48
1205	16	TBX22	P332H	Shared	0.15	0.30
1206	16	ELF4	L215I	Met Only	0.00	0.38
1207	16	GPC3	S220Y	Shared	0.16	0.38
1208	16	GPC3	N124K	Met Only	0.00	0.32
1209	17	NRAS	Q61K	Shared	0.29	0.37
1210	17	LHFP	G26E	Shared	0.16	0.13
1211	17	HIF1A	R54X	Shared	0.34	0.22
1212	17	GAS7	R244K	Shared	0.43	0.45
1213	17	ROCK2	K659T	Shared	0.31	0.36
1214	17	APC	Q1378X	Shared	0.64	0.51
1215	17	FLT4	G1296S	Shared	0.25	0.23
1216	17	CSMD1	K2420R	Shared	0.47	0.36
1217	17	CSMD1	V421I	Shared	0.41	0.33
1218	17	NFIB	F35L	Primary Only	0.05	0.00
1219	17	EP400	L1719F	Met Only	0.00	0.05
1220	17	TET2	Q373K	Met Only	0.00	0.03
1221	17	ARID5B	S451R	Met Only	0.00	0.04
1222	17	TP53	G245S	Shared	0.49	0.45
1223	17	ATRX	S1895R	Met Only	0.00	0.03
1224	18	HNF1A	A160T	Shared	0.34	0.40
1225	18	TP53	C135F	Shared	0.51	0.80
1226	18	PREX1	G1558E	Shared	0.10	0.11
1227	18	APC	R499X	Shared	0.48	0.72
1228	18	NSD1	Q925X	Shared	0.37	0.42
1229	18	CSMD1	K2937R	Shared	0.09	0.47
1230	18	SGK3	D55H	Shared	0.03	0.19

1231	18	NR4A3	R352Q	Shared	0.11	0.49
1232	18	ABCA1	T1083A	Shared	0.20	0.49
1233	18	AMER1	Q1042H	Primary Only	0.32	0.00
1234	18	CCNE1	A353T	Primary Only	0.09	0.00
1235	18	TP53	P278L	Primary Only	0.14	0.00
1236	18	ATRX	S2220X	Met Only	0.00	0.03
