# Supplementary material for: High-depth sequencing of over 750 genes supports linear progression of primary tumors and metastases in most patients with liver-limited metastatic colorectal cancer
Source: Genome Biol. 2015 Feb 12;16(1):32. doi: 10.1186/s13059-015-0589-1 (PMC4365969; doi:10.1186/s13059-015-0589-1)
Supplement: Additional file 5: Table S4. — Recurrent variants and their distribution in the primary tumor and metastasis. [file 13059_2015_589_MOESM5_ESM.pdf]

Supplementary Table 4: Recurrent Variants and their distribution in the primary tumor and metastasis.

Gene	variants found in only in the primary tumor	variants found in only in the metastasis	variants found in both primary and matched metastatic tissue	Total Number of variants in that gene
APC	3	1	21	25
XIRP2	5	4	14	23
FAT4	3	4	9	16
CSMD3	5	2	8	15
TP53	1	1	11	13
MLL3	3	8	2	13
AKAP9	4	1	7	12
CSMD1	2	3	6	11
LRRK2	0	0	11	11
NF1	1	1	8	10
ATM	2	2	5	9
SETD2	1	2	6	9
BAI3	2	1	5	8
PDE4DIP	0	4	4	8
SMG1	0	4	4	8
NAV3	2	1	4	7
CDH11	3	1	3	7
CNTRL	1	1	5	7
KDR	3	0	4	7
MACF1	1	0	6	7
MLL	2	2	3	7
N4BP2	0	3	4	7
PRDM16	1	1	5	7
PTPRT	2	3	2	7
ROCK2	0	0	7	7
TRIO	2	2	3	7
ERBB4	3	1	2	6
KRAS	0	0	6	6
FAT1	0	4	2	6
CDC27	4	1	1	6
HIF1A	0	2	4	6
MSH3	0	2	4	6
PREX2	2	2	2	6
TIAM1	0	4	2	6
USP6	0	3	3	6
PIK3CA	1	1	3	5
GLI3	0	1	4	5

TRRAP	1	2	2	5
ATRX	0	3	2	5
BRCA2	2	1	2	5
BRDT	0	0	5	5
BRIP1	0	1	4	5
CHD8	0	1	4	5
DAXX	0	0	5	5
DCLK1	3	0	2	5
DDX10	0	2	3	5
EPHA6	2	0	3	5
EZH2	0	1	4	5
FNDC1	0	1	4	5
GNAS	0	4	1	5
IL7R	0	1	4	5
MAST4	2	0	3	5
MECOM	0	3	2	5
POLE	1	1	3	5
PREX1	0	2	3	5
PTCH1	1	2	2	5
PTEN	0	3	2	5
SLK	0	2	3	5
SPEN	1	1	3	5
TET2	1	2	2	5
TRIP11	2	0	3	5
CAMTA1	0	1	3	4
CENPF	1	1	2	4
CLSPN	0	3	1	4
CLTCL1	0	0	4	4
DICER1	3	0	1	4
DLC1	0	1	3	4
EIF2AK4	1	2	1	4
EP400	1	3	0	4
ERCC5	0	1	3	4
EVC2	0	1	3	4
FANCA	0	2	2	4
FBXW7	0	1	3	4
FLT1	1	0	3	4
FN1	2	1	1	4
KDM5A	1	0	3	4
LAMA1	0	1	3	4
LAMA2	0	0	4	4
LIFR	0	1	3	4
MAP3K2	0	0	4	4
MAP3K3	1	1	2	4
MITF	0	0	4	4
MYH9	0	0	4	4

PBRM1	0	2	2	4
PCDHA13	0	1	3	4
PDGFRA	0	0	4	4
PEG3	1	1	2	4
PRKDC	1	2	1	4
PTPN11	0	0	4	4
RAD50	1	2	1	4
ROS1	1	0	3	4
SDK1	0	1	3	4
SMAD4	2	0	2	4
SPECC1	0	0	4	4
STAB1	0	1	3	4
WRN	0	0	4	4
ZNF521	0	1	3	4
ARHGAP26	0	1	2	3
ATR	0	0	3	3
BAZ2B	0	0	3	3
CARD11	1	0	2	3
CBLB	0	1	2	3
CDK12	0	0	3	3
CHD6	1	1	1	3
CREBBP	1	0	2	3
CSF1R	1	0	2	3
DDX5	0	1	2	3
DNMT3A	0	1	2	3
EGFR	1	1	1	3
EPHA3	1	1	1	3
EPHA4	0	3	0	3
EPHA7	0	0	3	3
FANCD2	1	1	1	3
FES	0	1	2	3
FOXO1	1	0	2	3
FOXP1	0	2	1	3
GATA1	0	0	3	3
GRM8	0	1	2	3
HSP90AB1	0	1	2	3
INSR	1	0	2	3
JAK1	0	1	2	3
LCP1	1	0	2	3
MAP2K4	0	0	3	3
MAP3K14	0	0	3	3
MAP3K4	1	0	2	3
MAP3K8	0	1	2	3
MET	0	1	2	3

MLF1	0	1	2	3
MLL2	1	2	0	3
MLLT10	0	0	3	3
MYH11	1	1	1	3
NBN	0	0	3	3
NCOR1	0	2	1	3
NEK11	1	1	1	3
NIN	0	0	3	3
NOTCH2	0	1	2	3
NSD1	0	2	1	3
NTRK3	1	0	2	3
NUP98	0	0	3	3
PARK2	0	0	3	3
PIK3CB	0	0	3	3
PIK3R1	0	0	3	3
PIK3R4	2	0	1	3
PMS1	1	1	1	3
PRDM1	0	1	2	3
RBFOX1	0	2	1	3
ROCK1	0	2	1	3
RSF1	0	2	1	3
RUNX1T1	0	0	3	3
SENP5	0	2	1	3
SF3B1	1	0	2	3
SIRT1	1	2	0	3
SNX29	0	0	3	3
STK3	1	1	1	3
TCERG1	1	0	2	3
TEX14	1	0	2	3
THRAP3	0	0	3	3
TOP2B	0	2	1	3
TPM4	0	1	2	3
TRIM33	1	1	1	3
ZMYM2	0	1	2	3
ABCA1	0	0	2	2
ADAMTS18	0	0	2	2
AFF3	0	0	2	2
ALK	0	0	2	2
ARID2	0	0	2	2
ARID4A	0	0	2	2
ARID5B	1	1	0	2
ASXL1	0	1	1	2
BMPR1B	0	1	1	2
BPTF	1	0	1	2
BRD8	1	1	0	2

CASK	1	0	1	2
CASP8	1	0	1	2
CCNE1	1	0	1	2
CDC73	0	1	1	2
CHN1	0	1	1	2
CIT	0	0	2	2
CLTC	0	0	2	2
COL1A1	0	1	1	2
CTNNA1	1	1	0	2
CTNND2	0	0	2	2
CXCR7	1	1	0	2
CYLD	0	2	0	2
EBF1	0	1	1	2
ECT2L	0	1	1	2
ELF4	0	1	1	2
EPHB1	1	0	1	2
EPS15	0	1	1	2
ERC1	0	1	1	2
ERCC2	1	0	1	2
ERCC4	1	0	1	2
FLT4	0	0	2	2
GAB2	1	0	1	2
GAK	0	0	2	2
GAS7	0	0	2	2
GLI2	1	0	1	2
GMPS	0	0	2	2
GNAQ	0	1	1	2
GPC3	0	1	1	2
GUCY1A2	0	0	2	2
HEY1	2	0	0	2
HOOK3	1	0	1	2
HUWE1	1	1	0	2
IGF1R	0	1	1	2
IKZF1	0	1	1	2
IL23R	1	0	1	2
JAK2	1	0	1	2
KAT6A	0	2	0	2
KDM5B	0	0	2	2
KIT	0	1	1	2
KTN1	1	0	1	2
LTBP1	0	1	1	2
MAGI3	0	1	1	2
MALAT1	0	0	2	2
MAP2K1	0	0	2	2
MAP3K1	2	0	0	2
MAP4K1	0	1	1	2

MAP4K3	1	0	1	2
MAP4K5	0	1	1	2
MAP7	1	1	0	2
MAPK6	2	0	0	2
MAPK9	0	1	1	2
MELK	0	0	2	2
MIER3	0	0	2	2
MINK1	0	1	1	2
MKL1	1	0	1	2
MLH1	0	1	1	2
MLLT3	0	1	1	2
MN1	0	0	2	2
MSH2	1	1	0	2
MSI2	0	1	1	2
MTOR	0	0	2	2
MYCN	0	1	1	2
MYO1B	0	1	1	2
MYO3A	0	0	2	2
NCOA4	0	1	1	2
NDRG1	0	0	2	2
NFIB	1	0	1	2
NLRP1	0	0	2	2
NR4A3	0	1	1	2
PALB2	0	2	0	2
PCM1	0	0	2	2
PLCG2	0	0	2	2
PMS2	0	1	1	2
PRCC	0	1	1	2
PRDM2	1	1	0	2
PSIP1	0	1	1	2
PTPRD	1	0	1	2
PTPRJ	1	0	1	2
RAD54B	0	1	1	2
RB1	1	0	1	2
RECQL4	0	0	2	2
REL	0	1	1	2
RET	0	1	1	2
RPS6KC1	0	0	2	2
SALL2	0	1	1	2
SCN5A	0	0	2	2
SCYL2	0	1	1	2
SDCCAG8	0	0	2	2
SDHAF2	0	0	2	2
SFPQ	0	0	2	2
SGK3	0	0	2	2
SMARCA2	0	2	0	2

SMARCC1	0	0	2	2
STK33	0	1	1	2
SUZ12	0	0	2	2
TAOK1	0	0	2	2
TEK	0	0	2	2
TET1	0	0	2	2
TIE1	1	0	1	2
TNKS2	0	1	1	2
TOP1	1	0	1	2
TRIM24	0	1	1	2
TSC1	0	1	1	2
TTK	0	0	2	2
UBR5	0	0	2	2
WHSC1	0	2	0	2
WHSC1L1	0	1	1	2
WNT5A	1	0	1	2
WWTR1	0	1	1	2
XPO1	0	2	0	2
ZNF217	0	0	2	2
