# Supplementary material for: High-depth sequencing of over 750 genes supports linear progression of primary tumors and metastases in most patients with liver-limited metastatic colorectal cancer
Source: Genome Biol. 2015 Feb 12;16(1):32. doi: 10.1186/s13059-015-0589-1 (PMC4365969; doi:10.1186/s13059-015-0589-1)
Supplement: Additional file 6: Table S5. — Ingenuity Pathway Analysis pathways identified that are unique to shared variants or to common variants. [file 13059_2015_589_MOESM6_ESM.pdf]

Supplementary Table 5. Ingenuity Pathway Analysis: pathways identified that are unique to shared variants or to common variants

Unique to shared	Unique to private
Glioma Invasiveness Signaling	Cardiomyocyte Differentiation via BMP Receptors
Virus Entry via Endocytic Pathways	Estrogen-mediated S-phase Entry
Melatonin Signaling	PXR/RXR Activation
Synaptic Long Term Potentiation	TWEAK Signaling
Role of Pattern Recognition Receptors in Recognition of Bacteria and Viruses	Remodeling of Epithelial Adherens Junctions
4-1BB Signaling in T Lymphocytes	Cell Cycle Regulation by BTG Family Proteins
Aldosterone Signaling in Epithelial Cells	TREM1 Signaling
Chemokine Signaling	Tight Junction Signaling
Clathrin-mediated Endocytosis Signaling	Notch Signaling
IL-17A Signaling in Gastric Cells	Transcriptional Regulatory Network in Embryonic Stem Cells
Role of IL-17F in Allergic Inflammatory Airway Diseases	
Î±-Adrenergic Signaling	
IL-10 Signaling	
Semaphorin Signaling in Neurons	
3-phosphoinositide Biosynthesis	
Actin Nucleation by ARP-WASP Complex	
Systemic Lupus Erythematosus Signaling	
Superpathway of Inositol Phosphate Compounds	
Role of JAK1, JAK2 and TYK2 in Interferon Signaling	
MIF Regulation of Innate Immunity	
Phospholipase C Signaling	

Toll-like Receptor Signaling	
Role of JAK2 in Hormone-like Cytokine Signaling	
VDR/RXR Activation	
Hepatic Cholestasis	
CCR5 Signaling in Macrophages	
Antioxidant Action of Vitamin C	
FXR/RXR Activation	
Caveolar-mediated Endocytosis Signaling	
TNFR2 Signaling	
Nur77 Signaling in T Lymphocytes	
Activation of IRF by Cytosolic Pattern Recognition Receptors	
GÎ±i Signaling	
Interferon Signaling	
Cdc42 Signaling	
Granzyme A Signaling	
