# Supplementary material for: High-depth sequencing of over 750 genes supports linear progression of primary tumors and metastases in most patients with liver-limited metastatic colorectal cancer
Source: Genome Biol. 2015 Feb 12;16(1):32. doi: 10.1186/s13059-015-0589-1 (PMC4365969; doi:10.1186/s13059-015-0589-1)
Supplement: Additional file 7: Figure S2. — Aggregate genomic alterations in each of the 18 patients. Top panel: somatic non-synonymous alterations in the primary (blue) and metastasis (red). Middle panel: B-allele frequency plots of heterozygous SNPs in tumor and normal tissue. The y-axis indicates deviation of B-allele frequency from 0.5 towards either 0 or 1; the x-axis indicates chromosomal number. Bottom panel: focal copy number estimates from Nanostring nCounter Cancer Copy Number v2 panel in the primary and metastasis. The y-axis indicates estimated copy number; the x-axis indicates chromosomal number. [file 13059_2015_589_MOESM7_ESM.pdf]

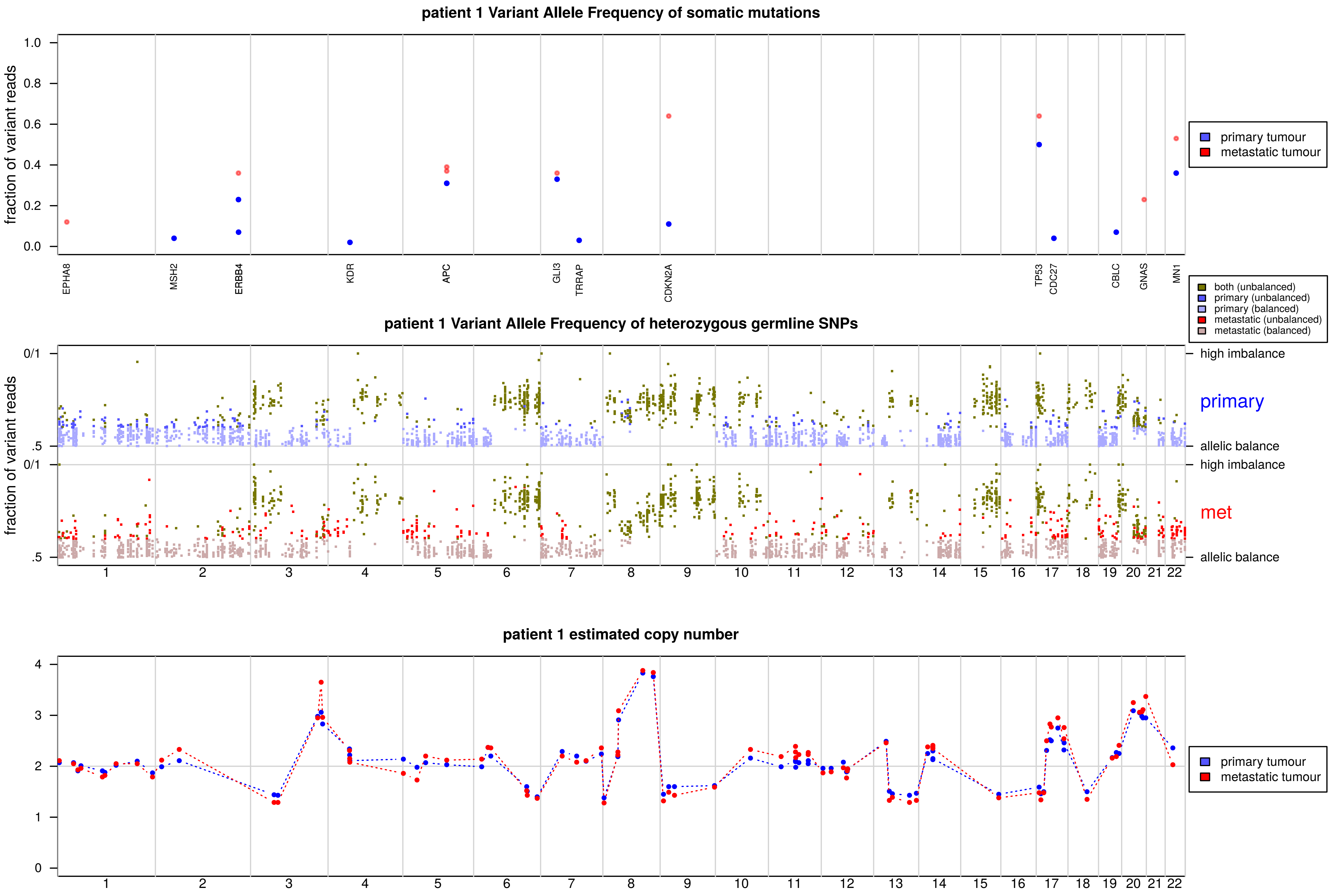
patient 1 Variant Allele Frequency of somatic mutations
fraction of variant reads
1.0
0.8
0.6
0.4
0.2
0.0
EPHA8
MSH2
ERBB4
KDR
APC
GLI3
TRRAP
CDKN2A
TP53
CDC27
CBLC
GNAS
MN1
primary tumour
metastatic tumour
patient 1 Variant Allele Frequency of heterozygous germline SNPs
both (unbalanced)
primary (unbalanced)
primary (balanced)
metastatic (unbalanced)
metastatic (balanced)
0/1
.5
high imbalance
primary
allelic balance
met
1 2 3 4 5 6 7 8 9 10 11 12 13 14 15 16 17 18 19 20 21 22
patient 1 estimated copy number
4
3
2
1
0
primary tumour
metastatic tumour

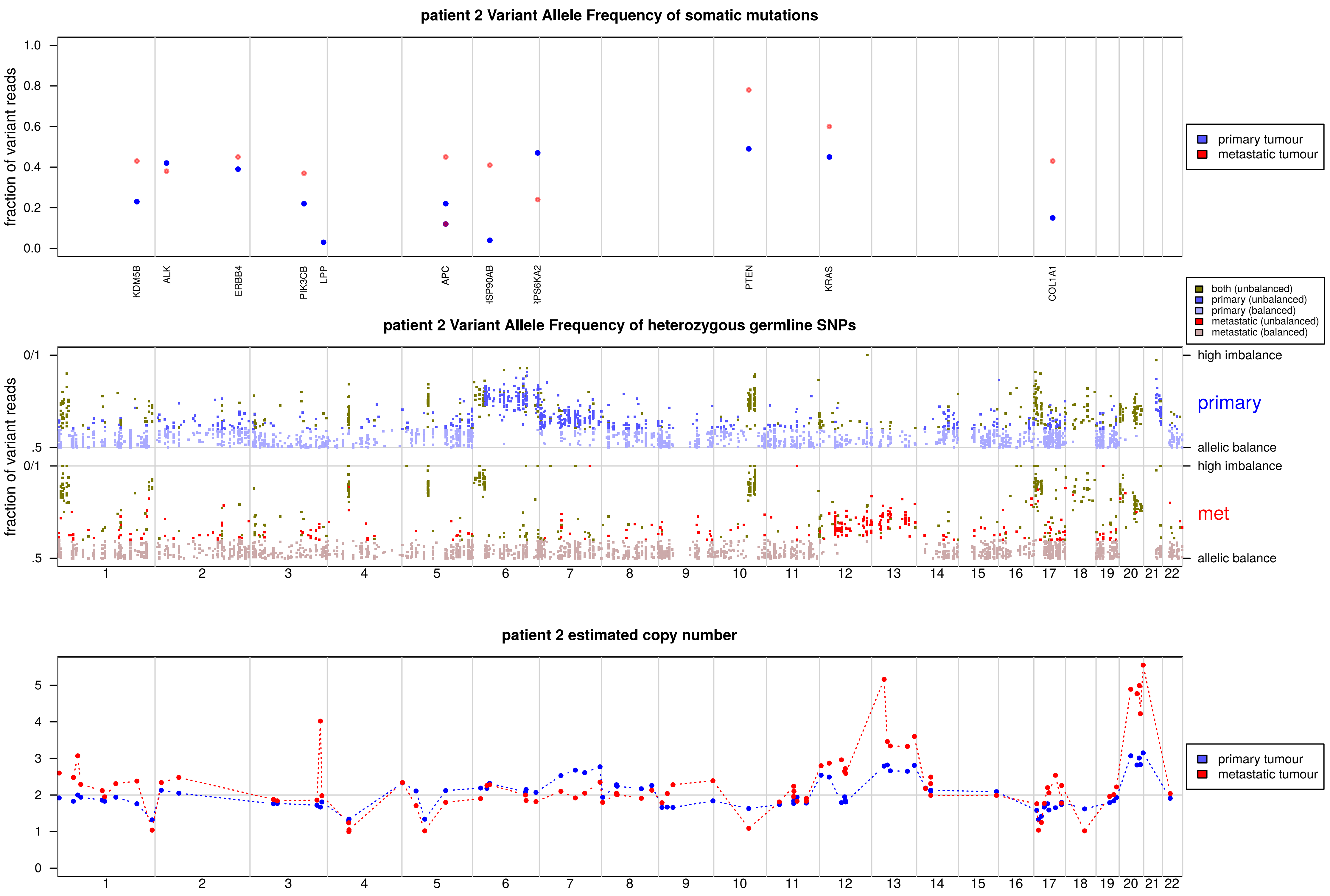

patient 2 Variant Allele Frequency of somatic mutations
fraction of variant reads
1.0
0.8
0.6
0.4
0.2
0.0
KDM5B
ALK
ERBB4
PIK3CB
LPP
APC
SP90AB
PS6KA2
PTEN
KRAS
COL1A1
primary tumour
metastatic tumour
patient 2 Variant Allele Frequency of heterozygous germline SNPs
both (unbalanced)
primary (unbalanced)
primary (balanced)
metastatic (unbalanced)
metastatic (balanced)
fraction of variant reads
0/1
.5
0/1
.5
high imbalance
primary
allelic balance
high imbalance
met
allelic balance
1 2 3 4 5 6 7 8 9 10 11 12 13 14 15 16 17 18 19 20 21 22
patient 2 estimated copy number
5
4
3
2
1
0
primary tumour
metastatic tumour
1 2 3 4 5 6 7 8 9 10 11 12 13 14 15 16 17 18 19 20 21 22

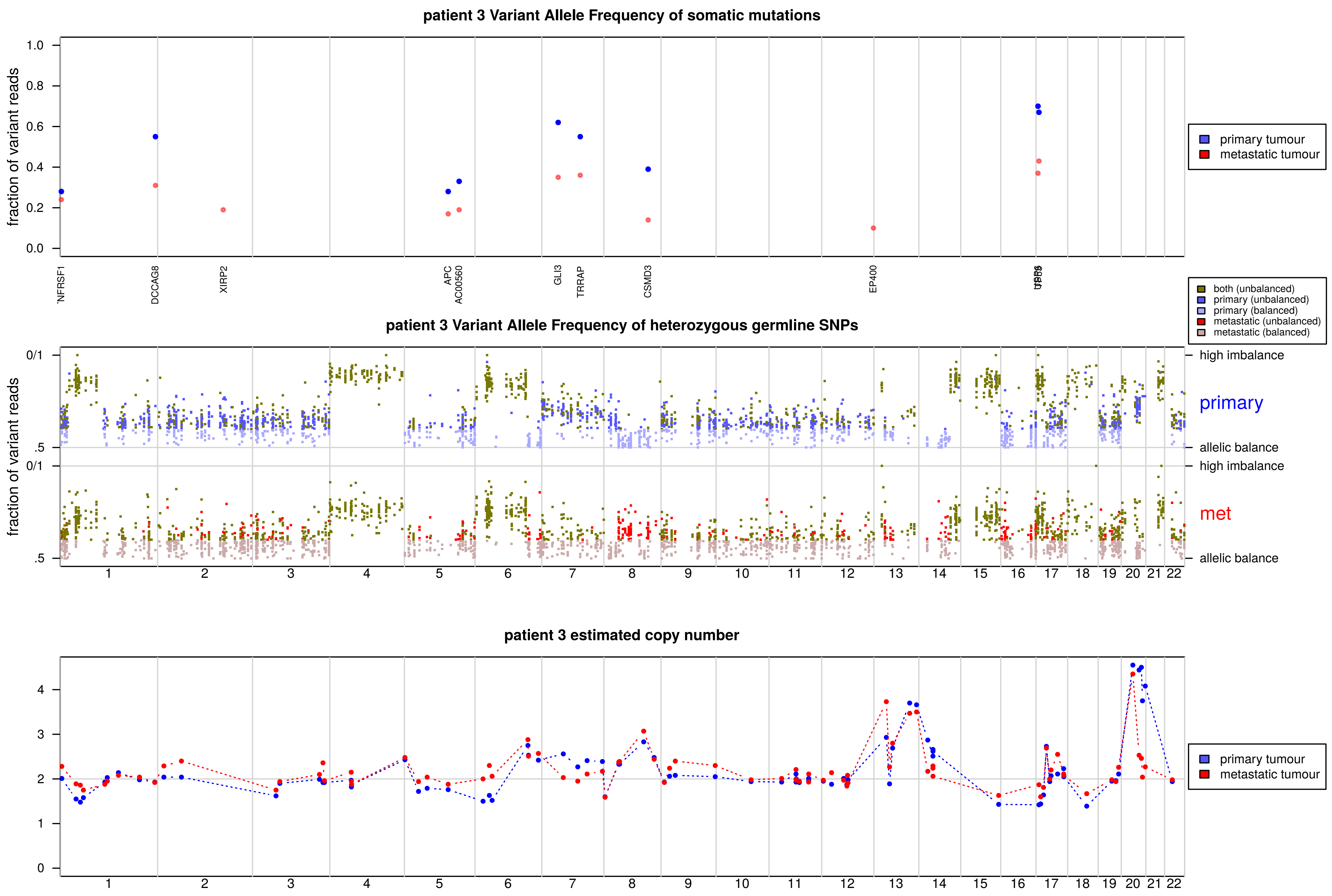

patient 3 Variant Allele Frequency of somatic mutations
fraction of variant reads
1.0
0.8
0.6
0.4
0.2
0.0
primary tumour
metastatic tumour
NFRSF1
DCCAG8
XIRP2
APC
AC00560
GLI3
TRRAP
CSMD3
EP400
both (unbalanced)
primary (unbalanced)
primary (balanced)
metastatic (unbalanced)
metastatic (balanced)
patient 3 Variant Allele Frequency of heterozygous germline SNPs
fraction of variant reads
0/1
.5
high imbalance
primary
allelic balance
met
1 2 3 4 5 6 7 8 9 10 11 12 13 14 15 16 17 18 19 20 21 22
patient 3 estimated copy number
0
1
2
3
4
primary tumour
metastatic tumour

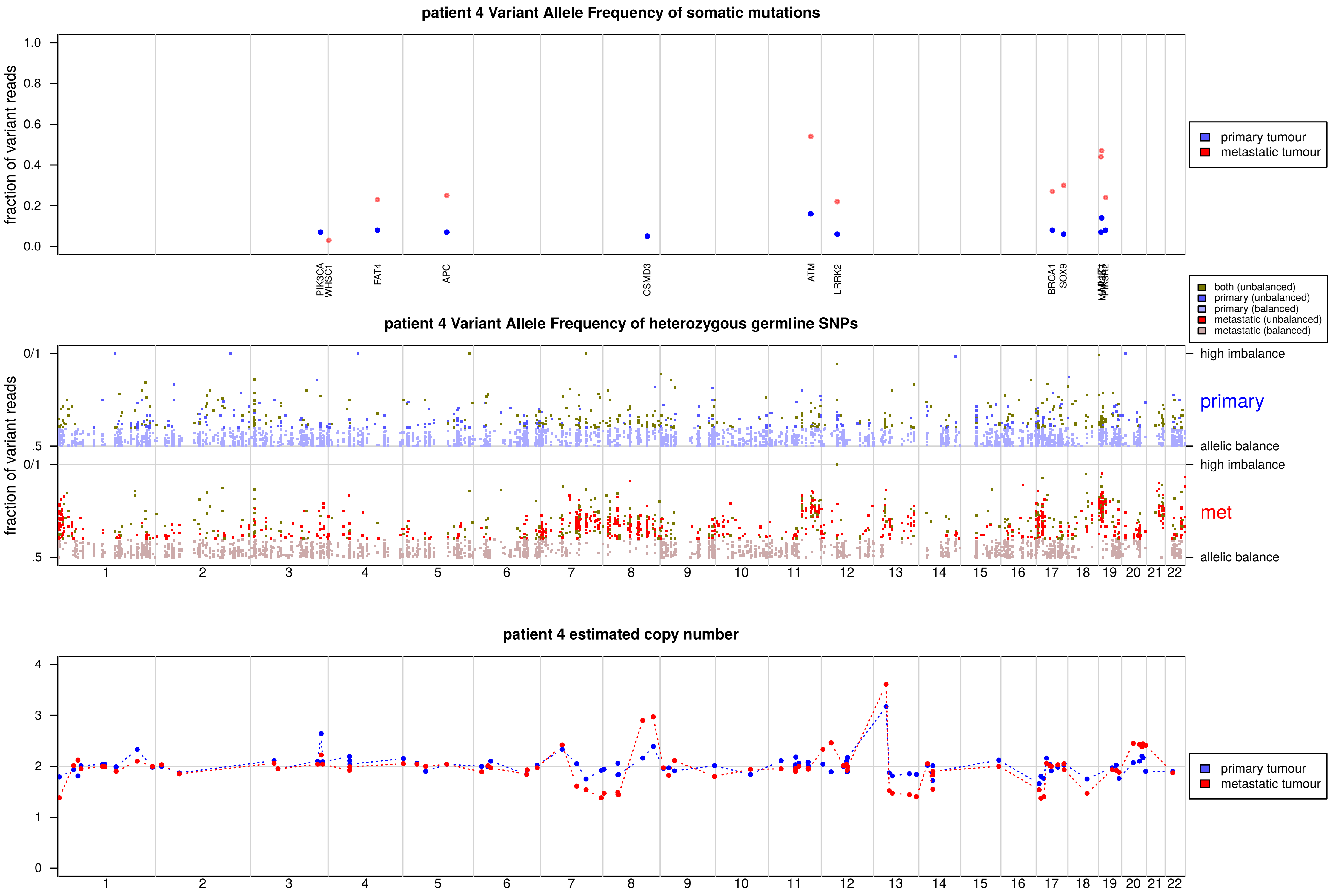
patient 4 Variant Allele Frequency of somatic mutations
fraction of variant reads
1.0
0.8
0.6
0.4
0.2
0.0
PIK3CA
WHSC1
FAT4
APC
CSMD3
ATM
LRRK2
BRCA1
SOX9
primary tumour
metastatic tumour
patient 4 Variant Allele Frequency of heterozygous germline SNPs
both (unbalanced)
primary (unbalanced)
primary (balanced)
metastatic (unbalanced)
metastatic (balanced)
0/1
.5
high imbalance
primary
allelic balance
met
1 2 3 4 5 6 7 8 9 10 11 12 13 14 15 16 17 18 19 20 21 22
patient 4 estimated copy number
4
3
2
1
0
primary tumour
metastatic tumour

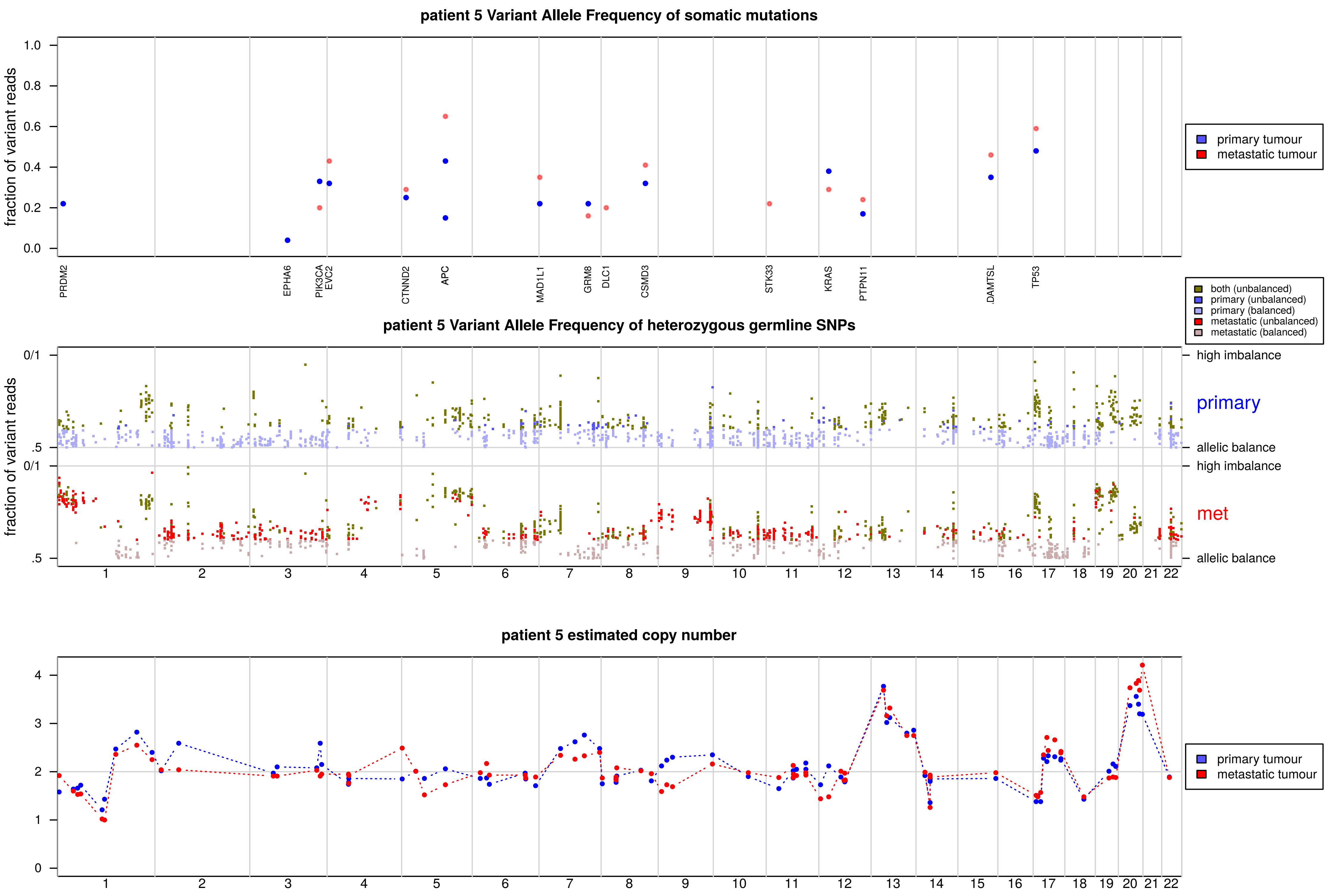
patient 5 Variant Allele Frequency of somatic mutations
fraction of variant reads
primary tumour
metastatic tumour
PRDM2
EPHA6
PIK3CA
EVC2
CTNND2
APC
MAD1L1
GRM8
DLC1
CSMD3
STK33
KRAS
PTPN11
TP53
both (unbalanced)
primary (unbalanced)
primary (balanced)
metastatic (unbalanced)
metastatic (balanced)
patient 5 Variant Allele Frequency of heterozygous germline SNPs
high imbalance
primary
allelic balance
met
patient 5 estimated copy number

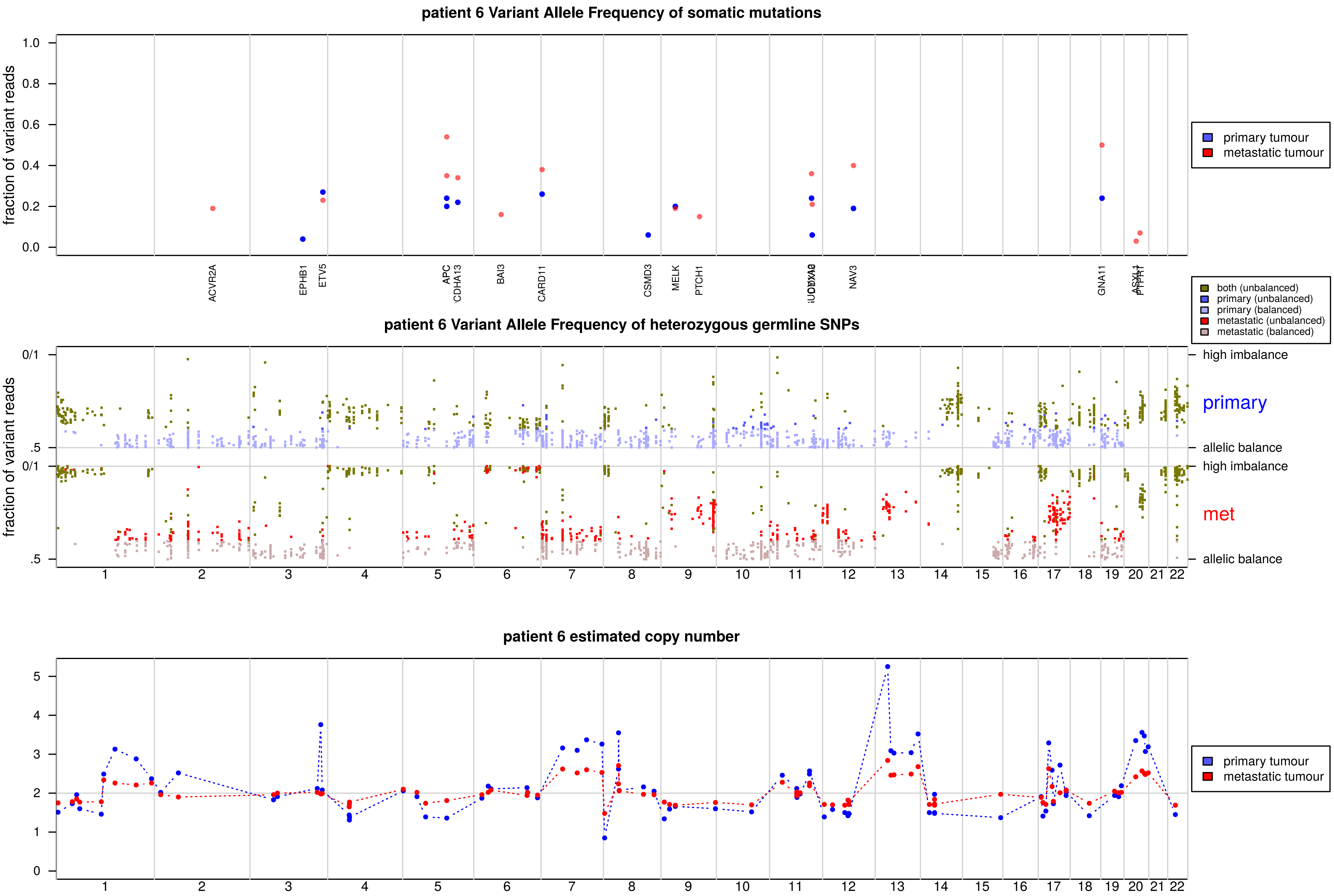

patient 6 Variant Allele Frequency of somatic mutations
fraction of variant reads
1.0
0.8
0.6
0.4
0.2
0.0
primary tumour
metastatic tumour
ACVR2A
EPHB1
ETV5
APC
CDHA13
BAI3
CARD11
CSMD3
MELK
PTCH1
NAV3
GNA11
patient 6 Variant Allele Frequency of heterozygous germline SNPs
both (unbalanced)
primary (unbalanced)
primary (balanced)
metastatic (unbalanced)
metastatic (balanced)
fraction of variant reads
0/1
.5
0/1
.5
high imbalance
primary
allelic balance
high imbalance
met
allelic balance
1 2 3 4 5 6 7 8 9 10 11 12 13 14 15 16 17 18 19 20 21 22
patient 6 estimated copy number
5
4
3
2
1
0
primary tumour
metastatic tumour
1 2 3 4 5 6 7 8 9 10 11 12 13 14 15 16 17 18 19 20 21 22

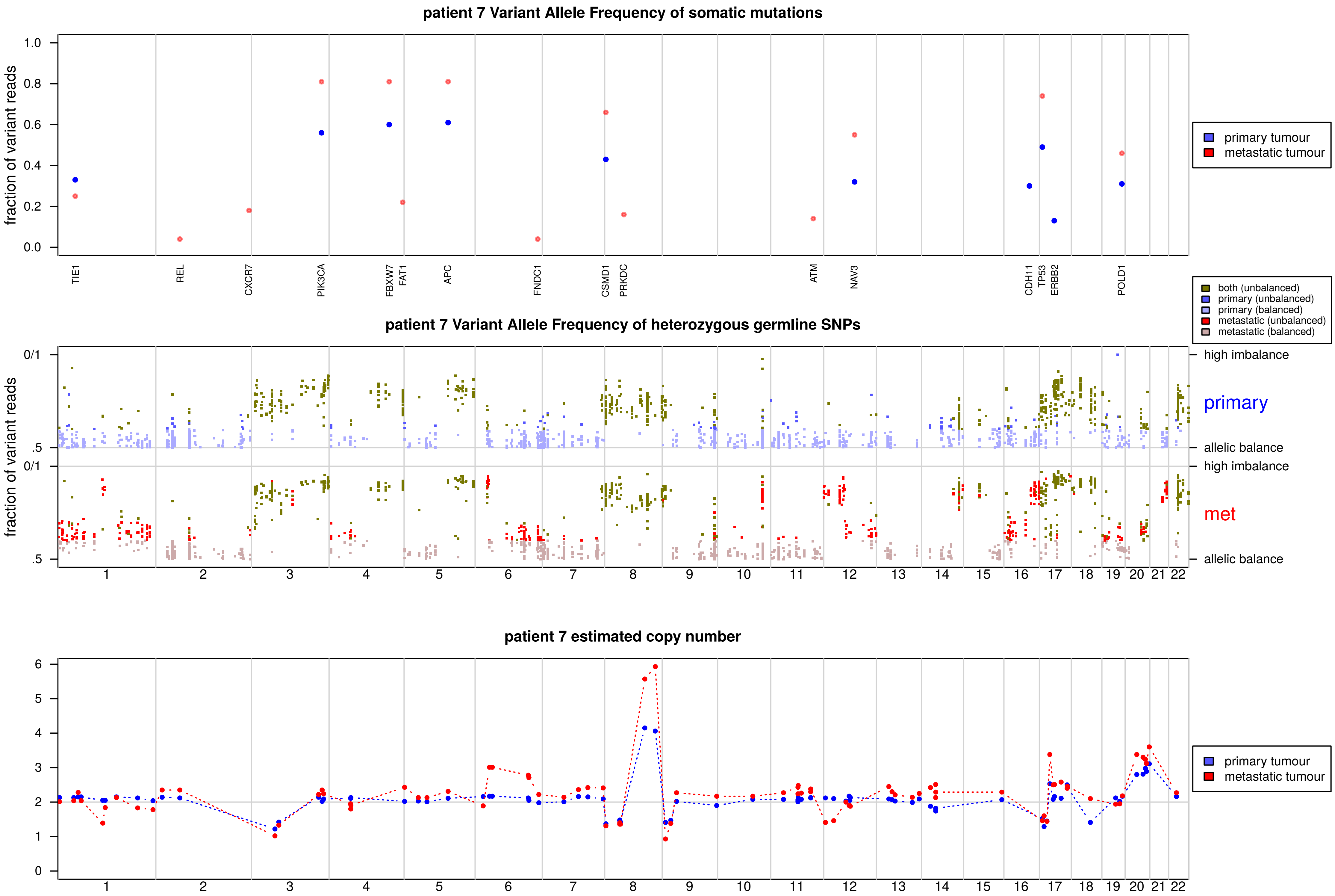
patient 7 Variant Allele Frequency of somatic mutations
fraction of variant reads
1.0
0.8
0.6
0.4
0.2
0.0
TIE1
REL
CXCR7
PIK3CA
FBXW7
FAT1
APC
FNDC1
CSMD1
PRKDC
ATM
NAV3
CDH11
TP53
ERBB2
POLD1
primary tumour
metastatic tumour
patient 7 Variant Allele Frequency of heterozygous germline SNPs
both (unbalanced)
primary (unbalanced)
primary (balanced)
metastatic (unbalanced)
metastatic (balanced)
fraction of variant reads
0/1
.5
0/1
.5
high imbalance
primary
allelic balance
high imbalance
met
allelic balance
1 2 3 4 5 6 7 8 9 10 11 12 13 14 15 16 17 18 19 20 21 22
patient 7 estimated copy number
6
5
4
3
2
1
0
primary tumour
metastatic tumour
1 2 3 4 5 6 7 8 9 10 11 12 13 14 15 16 17 18 19 20 21 22

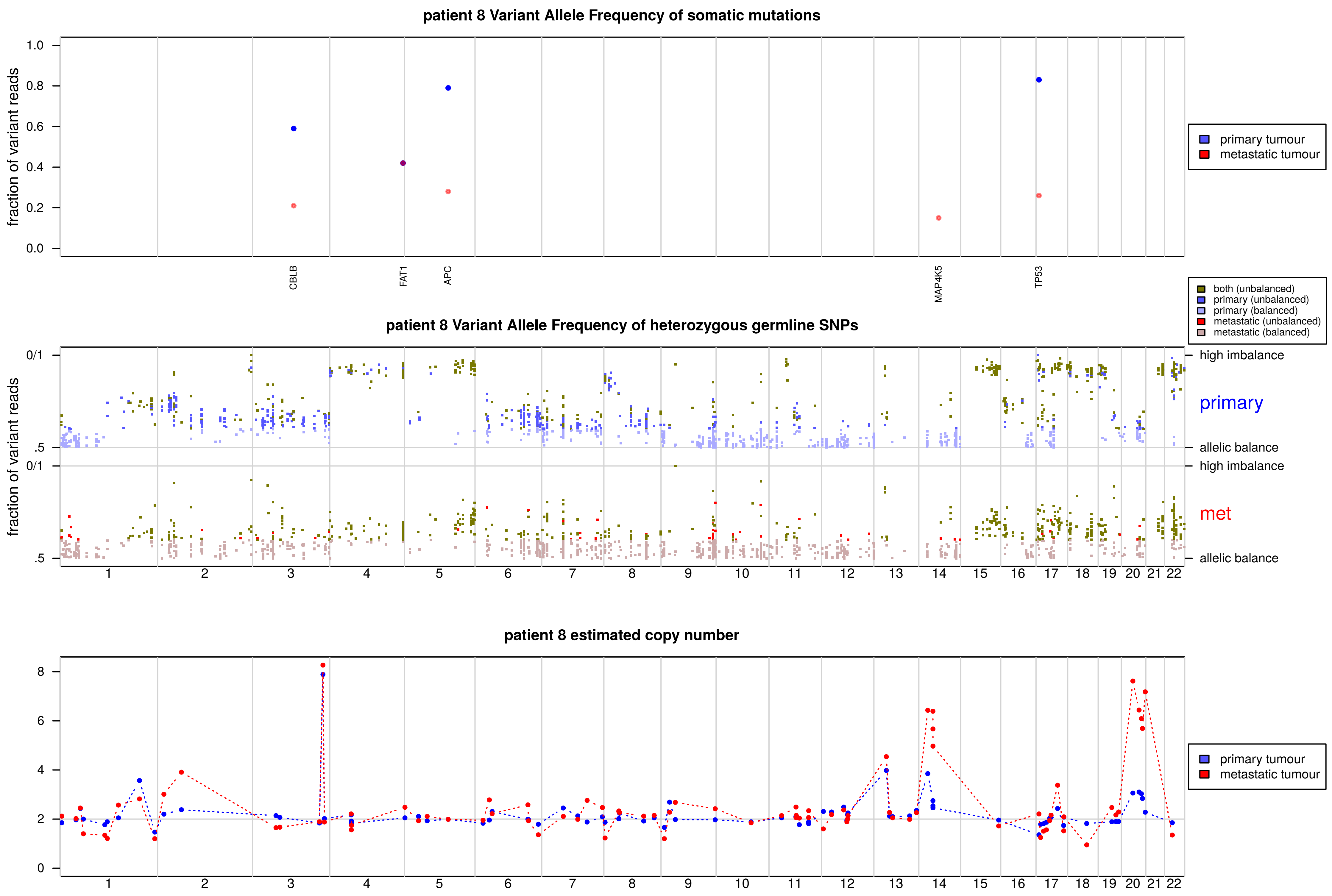
patient 8 Variant Allele Frequency of somatic mutations
fraction of variant reads
1.0
0.8
0.6
0.4
0.2
0.0
CBLB
FAT1
APC
MAP4K5
TP53
primary tumour
metastatic tumour
both (unbalanced)
primary (unbalanced)
primary (balanced)
metastatic (unbalanced)
metastatic (balanced)
patient 8 Variant Allele Frequency of heterozygous germline SNPs
fraction of variant reads
0/1
.5
0/1
.5
high imbalance
primary
allelic balance
high imbalance
met
allelic balance
1 2 3 4 5 6 7 8 9 10 11 12 13 14 15 16 17 18 19 20 21 22
patient 8 estimated copy number
8
6
4
2
0
primary tumour
metastatic tumour
1 2 3 4 5 6 7 8 9 10 11 12 13 14 15 16 17 18 19 20 21 22

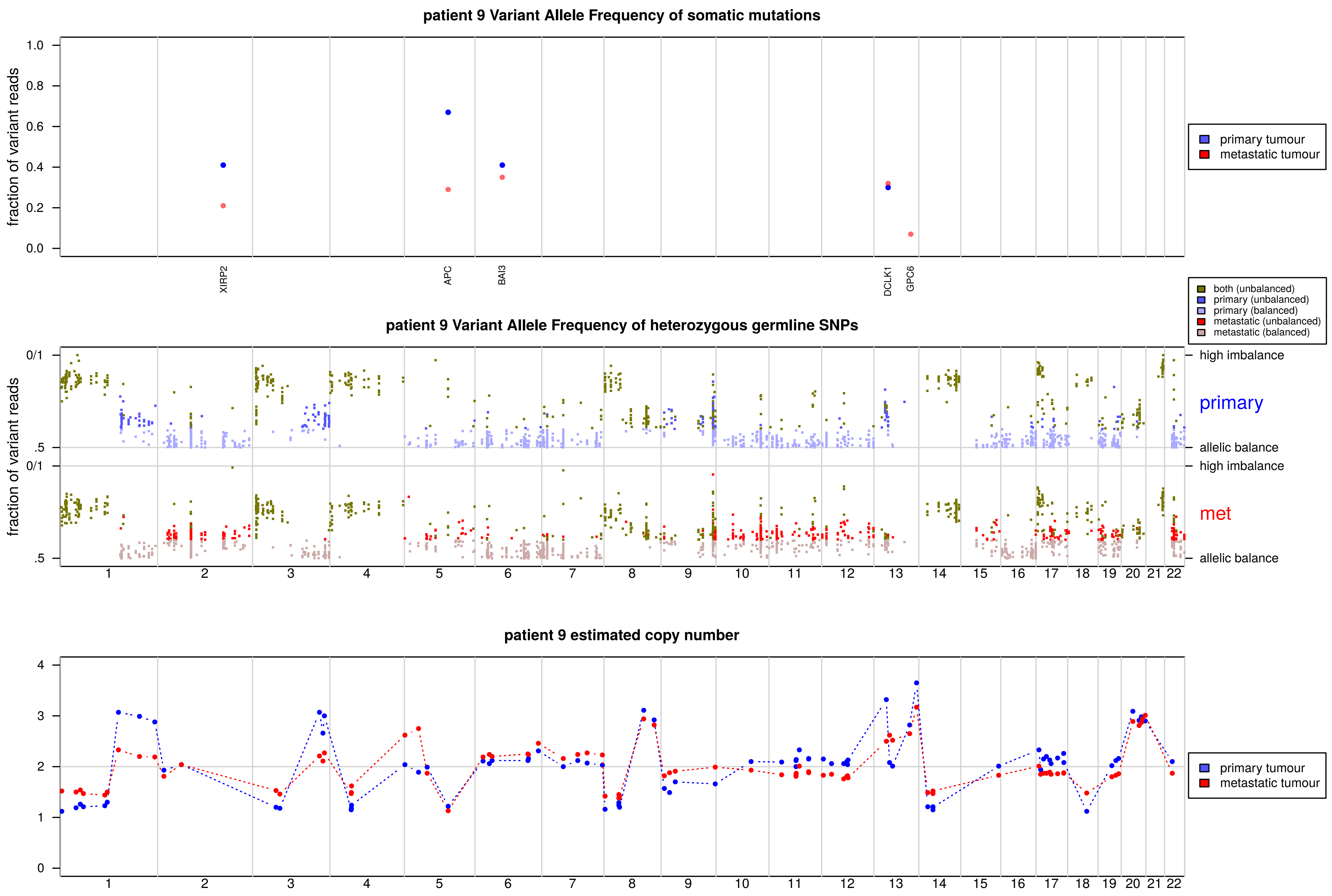
patient 9 Variant Allele Frequency of somatic mutations
fraction of variant reads
1.0
0.8
0.6
0.4
0.2
0.0
XIRP2
APC
BAI3
DCLK1
GPC6
primary tumour
metastatic tumour
both (unbalanced)
primary (unbalanced)
primary (balanced)
metastatic (unbalanced)
metastatic (balanced)
patient 9 Variant Allele Frequency of heterozygous germline SNPs
fraction of variant reads
0/1
.5
0/1
.5
high imbalance
primary
allelic balance
high imbalance
met
allelic balance
1 2 3 4 5 6 7 8 9 10 11 12 13 14 15 16 17 18 19 20 21 22
patient 9 estimated copy number
4
3
2
1
0
1 2 3 4 5 6 7 8 9 10 11 12 13 14 15 16 17 18 19 20 21 22
primary tumour
metastatic tumour

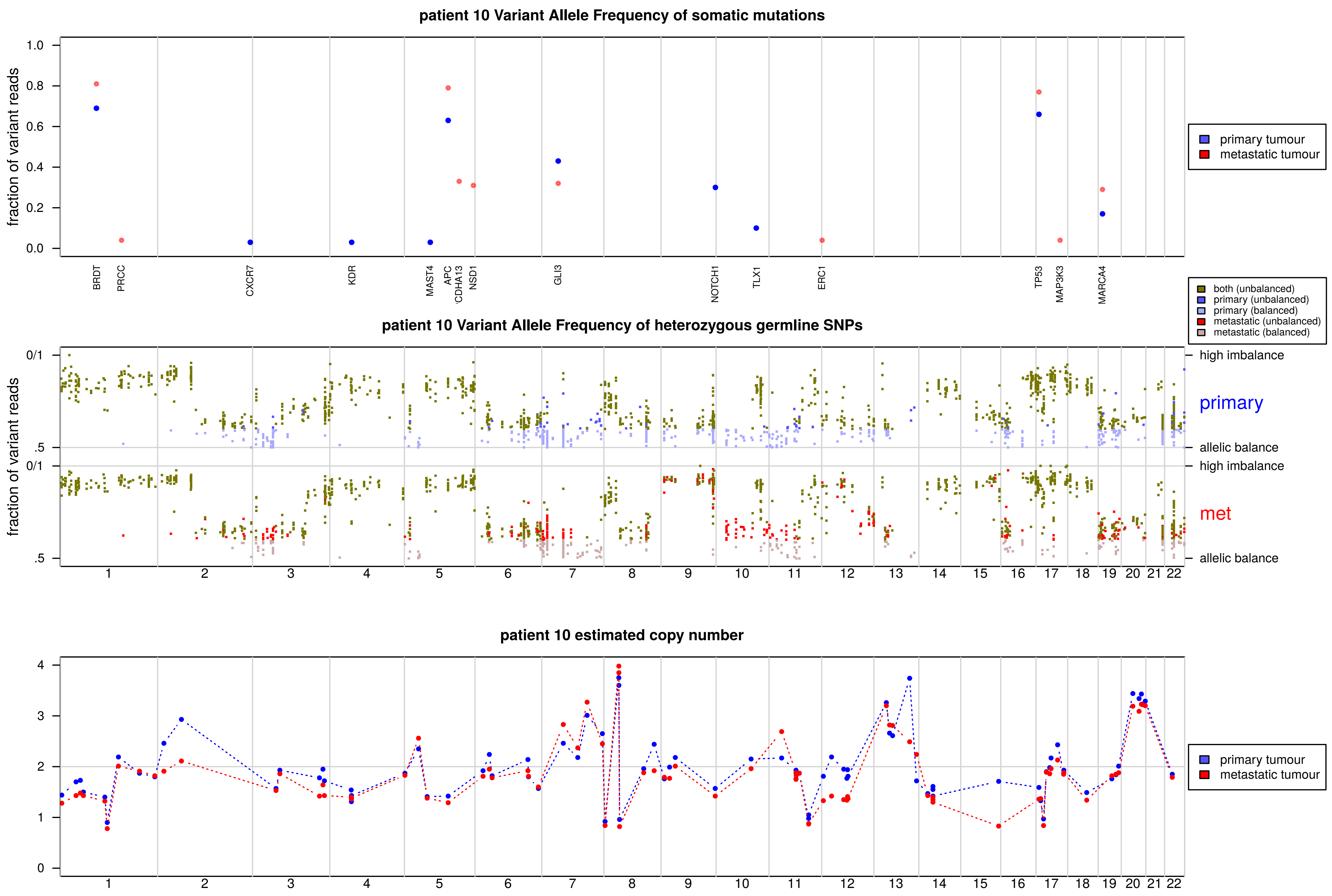
patient 10 Variant Allele Frequency of somatic mutations
fraction of variant reads
1.0
0.8
0.6
0.4
0.2
0.0
primary tumour
metastatic tumour
BRDT
PRCC
CXCR7
KDR
MAST4
APC
CDHA13
NSD1
GLI3
NOTCH1
TLX1
ERC1
TP53
MAP3K3
MARCA4
both (unbalanced)
primary (unbalanced)
primary (balanced)
metastatic (unbalanced)
metastatic (balanced)
patient 10 Variant Allele Frequency of heterozygous germline SNPs
fraction of variant reads
0/1
.5
high imbalance
primary
allelic balance
met
1 2 3 4 5 6 7 8 9 10 11 12 13 14 15 16 17 18 19 20 21 22
patient 10 estimated copy number
4
3
2
1
0
primary tumour
metastatic tumour
1 2 3 4 5 6 7 8 9 10 11 12 13 14 15 16 17 18 19 20 21 22

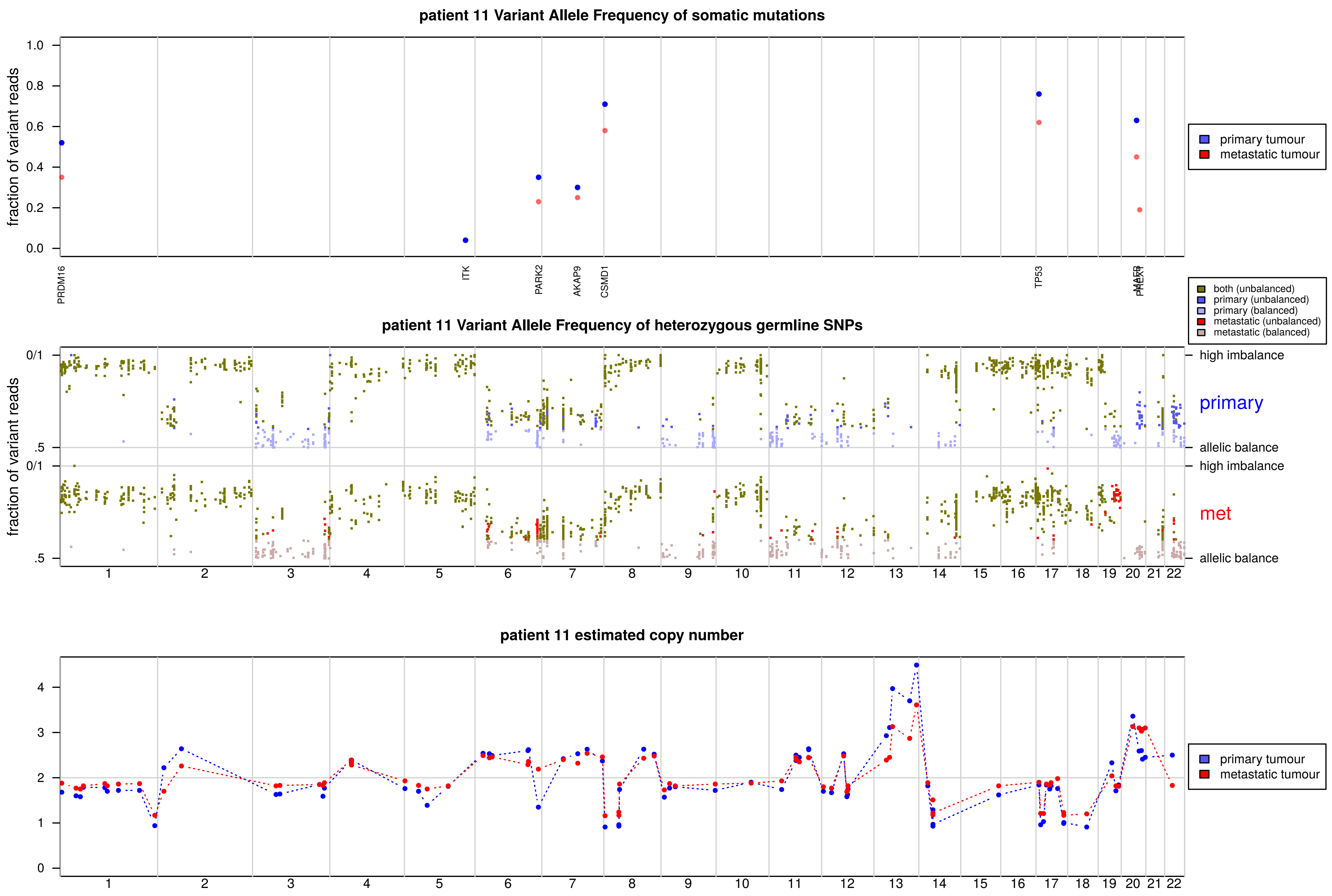

patient 11 Variant Allele Frequency of somatic mutations
fraction of variant reads
1.0
0.8
0.6
0.4
0.2
0.0
primary tumour
metastatic tumour
PRDM16
ITK
PARK2
AKAP9
CSMD1
TP53
PREX1
patient 11 Variant Allele Frequency of heterozygous germline SNPs
both (unbalanced)
primary (unbalanced)
primary (balanced)
metastatic (unbalanced)
metastatic (balanced)
0/1
.5
high imbalance
primary
allelic balance
met
1 2 3 4 5 6 7 8 9 10 11 12 13 14 15 16 17 18 19 20 21 22
patient 11 estimated copy number
4
3
2
1
0
primary tumour
metastatic tumour
1 2 3 4 5 6 7 8 9 10 11 12 13 14 15 16 17 18 19 20 21 22

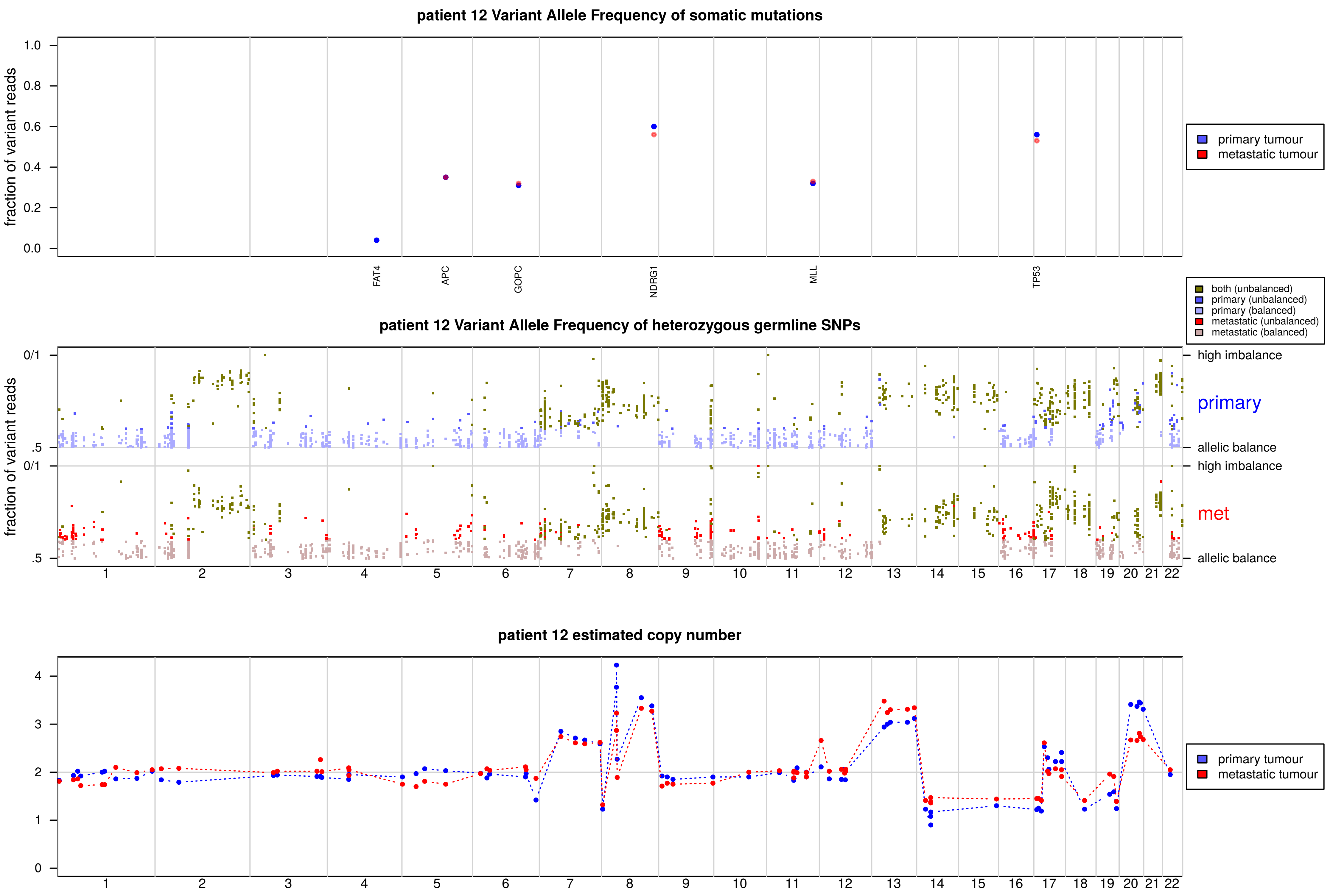

patient 12 Variant Allele Frequency of somatic mutations
fraction of variant reads
FAT4
APC
GOPC
NDRG1
MLL
TP53
primary tumour
metastatic tumour
patient 12 Variant Allele Frequency of heterozygous germline SNPs
both (unbalanced)
primary (unbalanced)
primary (balanced)
metastatic (unbalanced)
metastatic (balanced)
high imbalance
primary
allelic balance
high imbalance
met
allelic balance
patient 12 estimated copy number
primary tumour
metastatic tumour

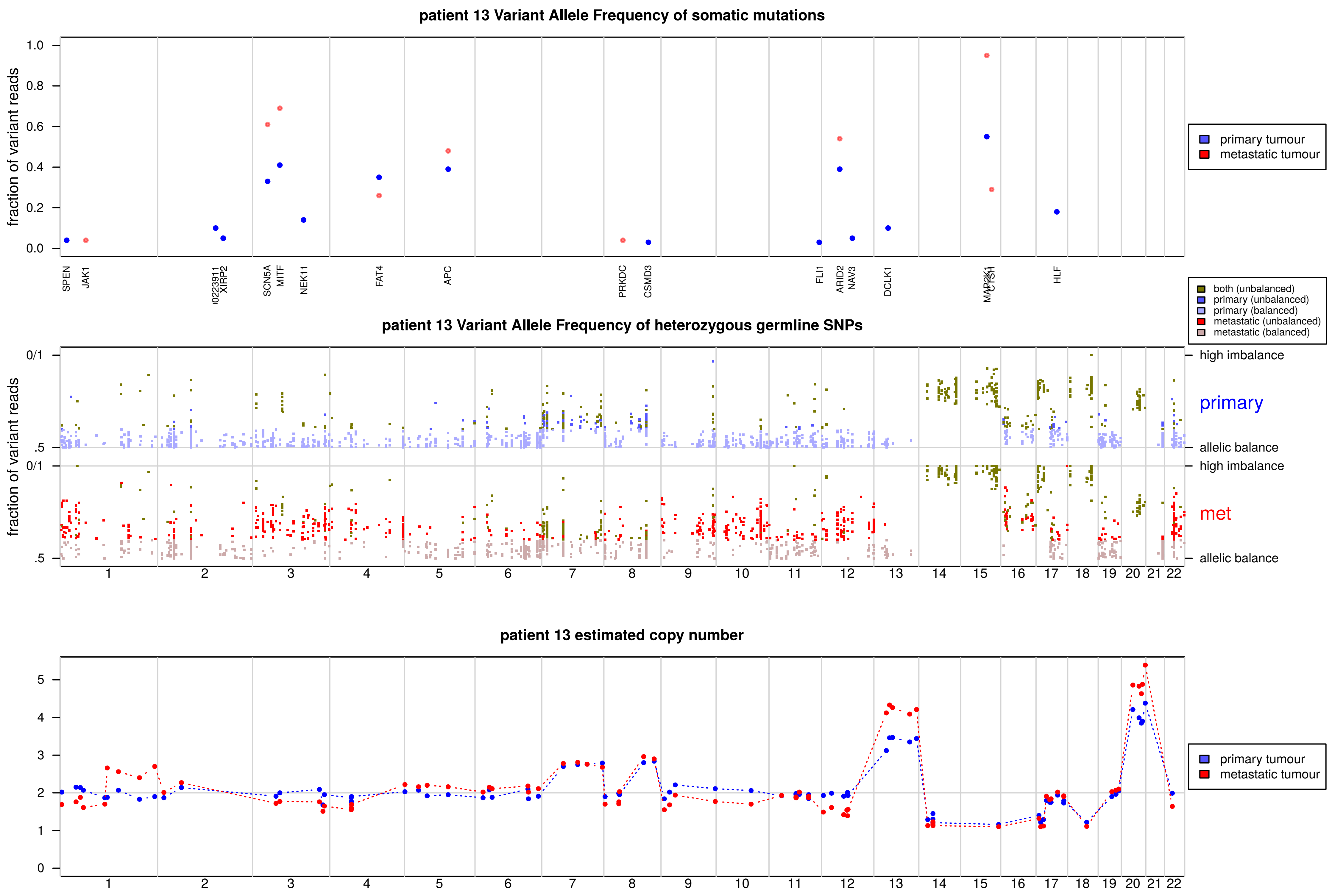

patient 13 Variant Allele Frequency of somatic mutations
fraction of variant reads
1.0
0.8
0.6
0.4
0.2
0.0
primary tumour
metastatic tumour
SPEN
JAK1
XIRP2
SCN5A
MITF
NEK11
FAT4
APC
PRKDC
CSMD3
FLI1
ARID2
NAV3
DCLK1
HLF
patient 13 Variant Allele Frequency of heterozygous germline SNPs
both (unbalanced)
primary (unbalanced)
primary (balanced)
metastatic (unbalanced)
metastatic (balanced)
0/1
.5
high imbalance
primary
allelic balance
met
1 2 3 4 5 6 7 8 9 10 11 12 13 14 15 16 17 18 19 20 21 22
patient 13 estimated copy number
5
4
3
2
1
0
primary tumour
metastatic tumour
1 2 3 4 5 6 7 8 9 10 11 12 13 14 15 16 17 18 19 20 21 22

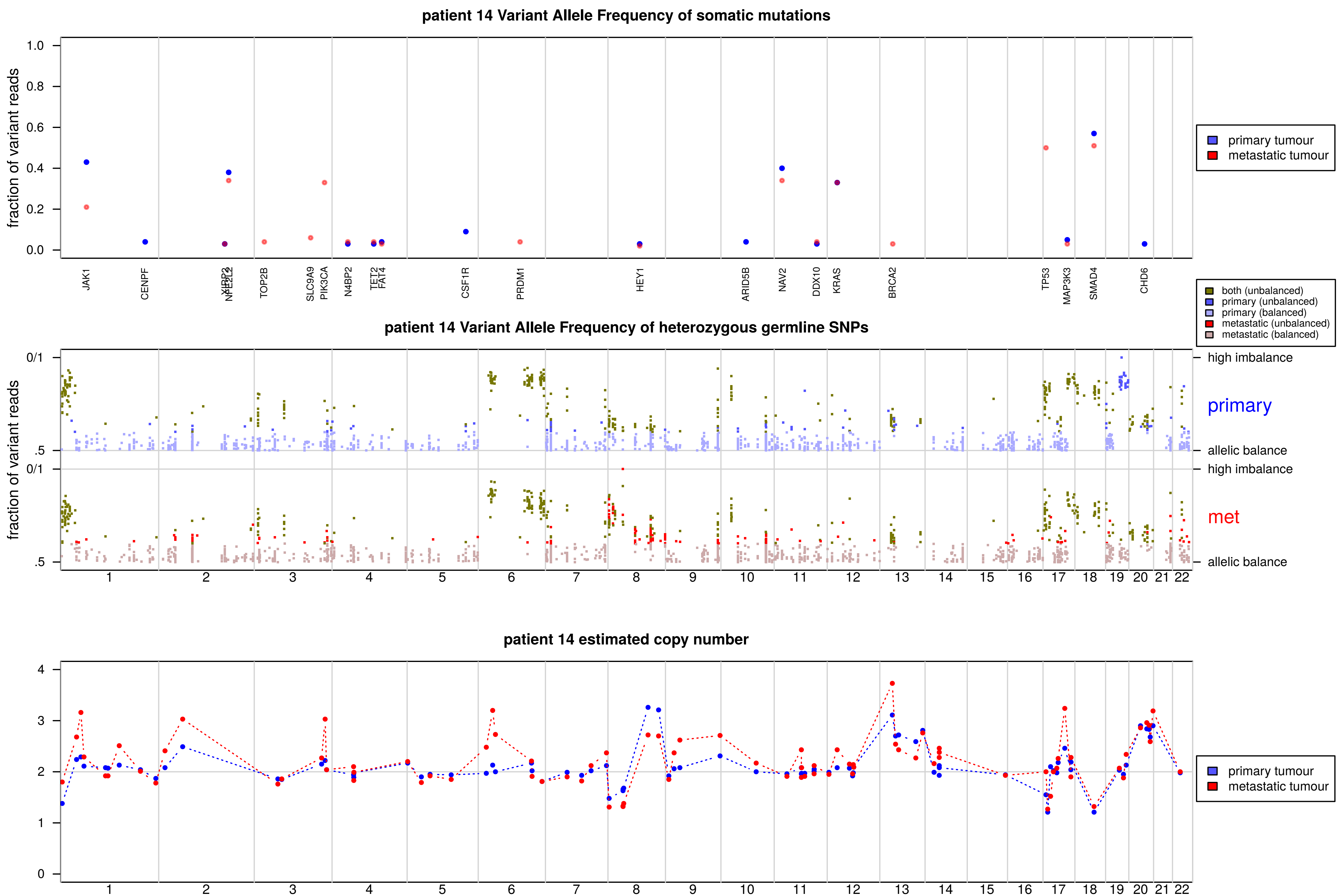

patient 14 Variant Allele Frequency of somatic mutations
fraction of variant reads
0.0
0.2
0.4
0.6
0.8
1.0
primary tumour
metastatic tumour
JAK1
CENPF
NFE2L2
TOP2B
SLC9A9
PIK3CA
N4BP2
TET2
FAT4
CSF1R
PRDM1
HEY1
ARID5B
NAV2
DDX10
KRAS
BRCA2
TP53
MAP3K3
SMAD4
CHD6
both (unbalanced)
primary (unbalanced)
primary (balanced)
metastatic (unbalanced)
metastatic (balanced)
patient 14 Variant Allele Frequency of heterozygous germline SNPs
fraction of variant reads
0/1
.5
high imbalance
primary
allelic balance
met
1 2 3 4 5 6 7 8 9 10 11 12 13 14 15 16 17 18 19 20 21 22
patient 14 estimated copy number
0
1
2
3
4
primary tumour
metastatic tumour
1 2 3 4 5 6 7 8 9 10 11 12 13 14 15 16 17 18 19 20 21 22

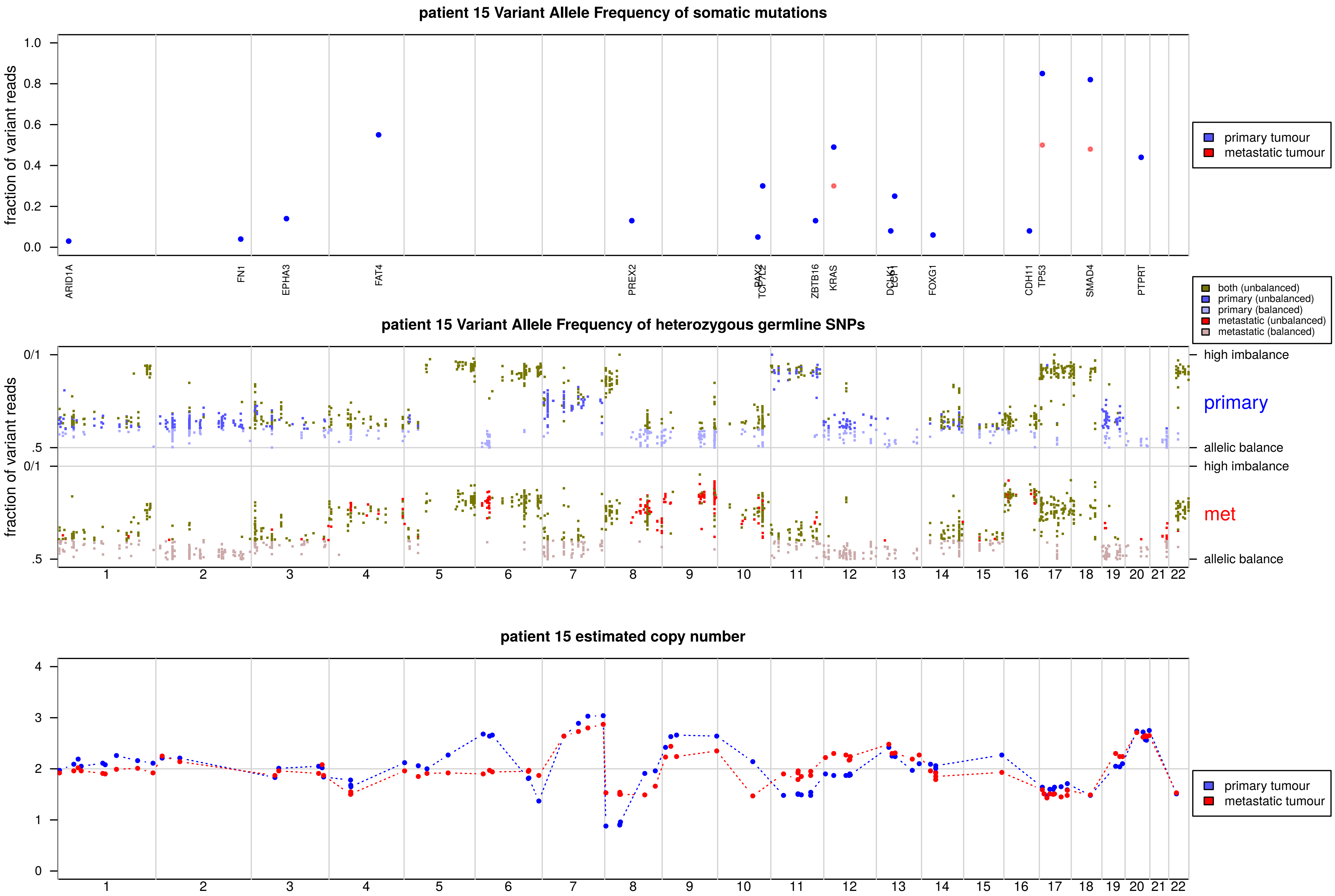
patient 15 Variant Allele Frequency of somatic mutations
fraction of variant reads
0.0
0.2
0.4
0.6
0.8
1.0
ARID1A
FN1
EPHA3
FAT4
PREX2
PAX3
TCF7L2
ZBTB16
KRAS
DCLK1
LCP1
FOXG1
CDH11
TP53
SMAD4
PTPRT
primary tumour
metastatic tumour
patient 15 Variant Allele Frequency of heterozygous germline SNPs
both (unbalanced)
primary (unbalanced)
primary (balanced)
metastatic (unbalanced)
metastatic (balanced)
0/1
.5
high imbalance
primary
allelic balance
met
1 2 3 4 5 6 7 8 9 10 11 12 13 14 15 16 17 18 19 20 21 22
patient 15 estimated copy number
0
1
2
3
4
primary tumour
metastatic tumour

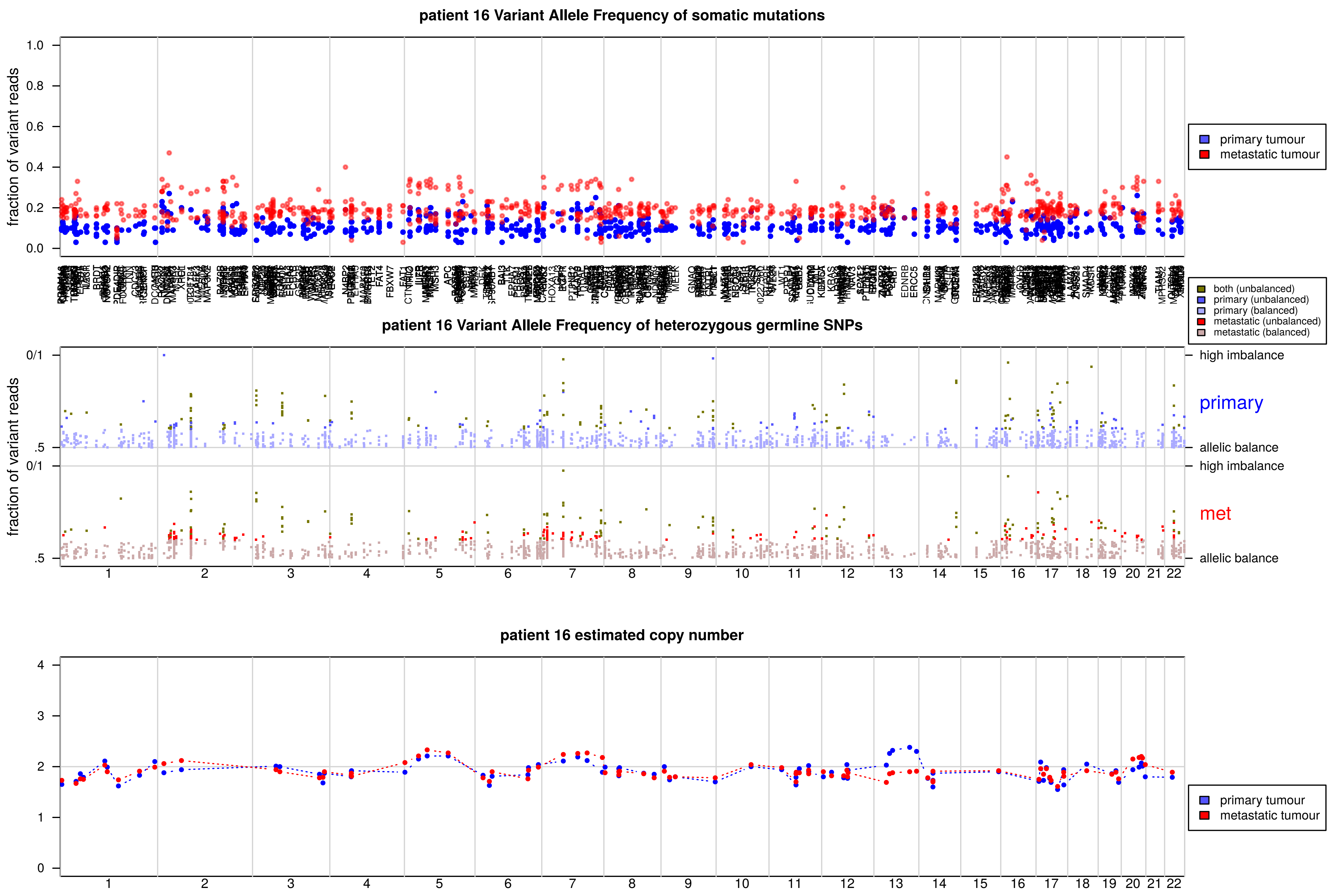

patient 16 Variant Allele Frequency of somatic mutations
fraction of variant reads
primary tumour
metastatic tumour
patient 16 Variant Allele Frequency of heterozygous germline SNPs
both (unbalanced)
primary (unbalanced)
primary (balanced)
metastatic (unbalanced)
metastatic (balanced)
high imbalance
primary
allelic balance
met
patient 16 estimated copy number

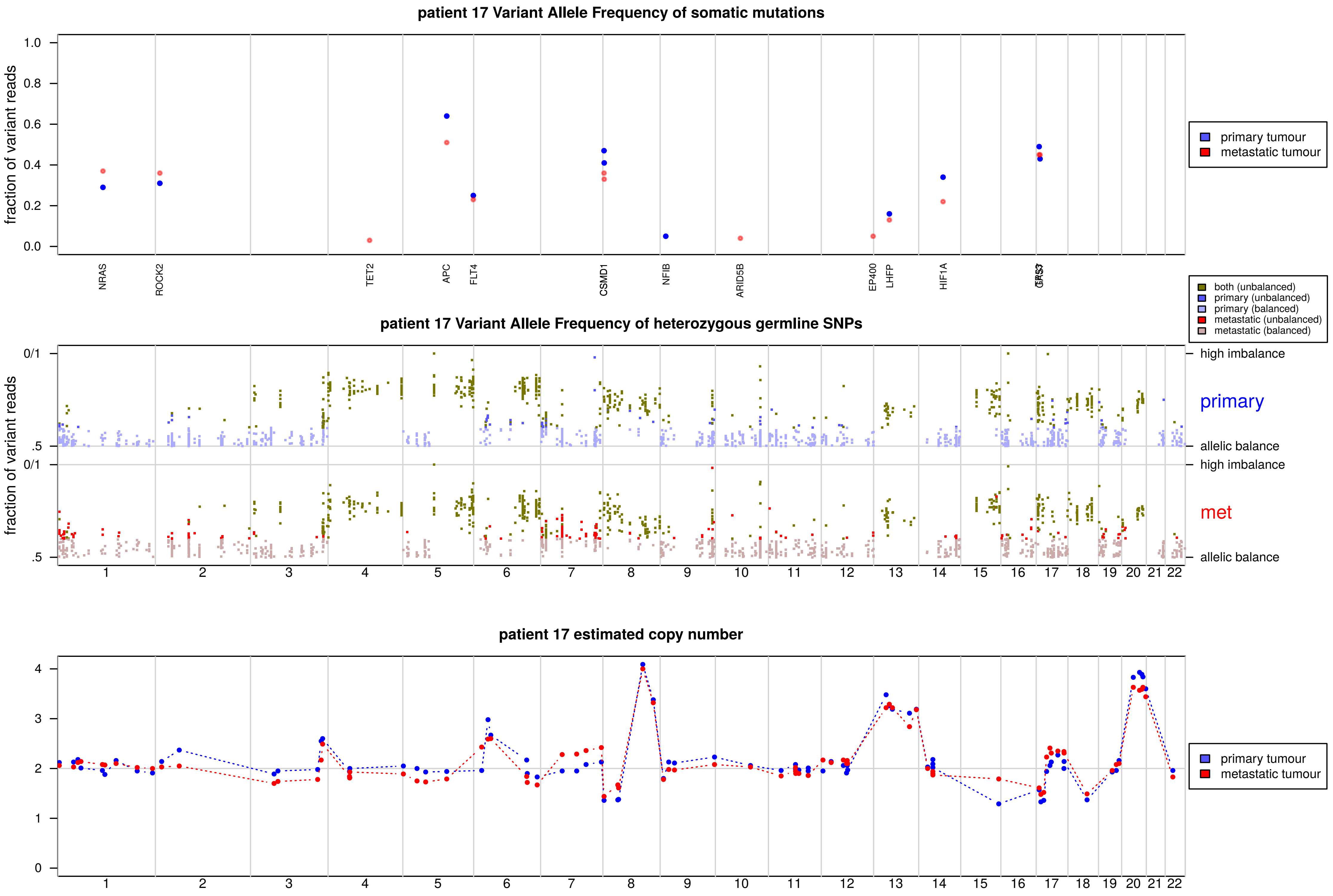
patient 17 Variant Allele Frequency of somatic mutations
fraction of variant reads
1.0
0.8
0.6
0.4
0.2
0.0
NRAS
ROCK2
TET2
APC
FLT4
CSMD1
NFIB
ARID5B
EP400
LHFP
HIF1A
primary tumour
metastatic tumour
both (unbalanced)
primary (unbalanced)
primary (balanced)
metastatic (unbalanced)
metastatic (balanced)
patient 17 Variant Allele Frequency of heterozygous germline SNPs
fraction of variant reads
0/1
.5
high imbalance
primary
allelic balance
met
1 2 3 4 5 6 7 8 9 10 11 12 13 14 15 16 17 18 19 20 21 22
patient 17 estimated copy number
4
3
2
1
0
primary tumour
metastatic tumour

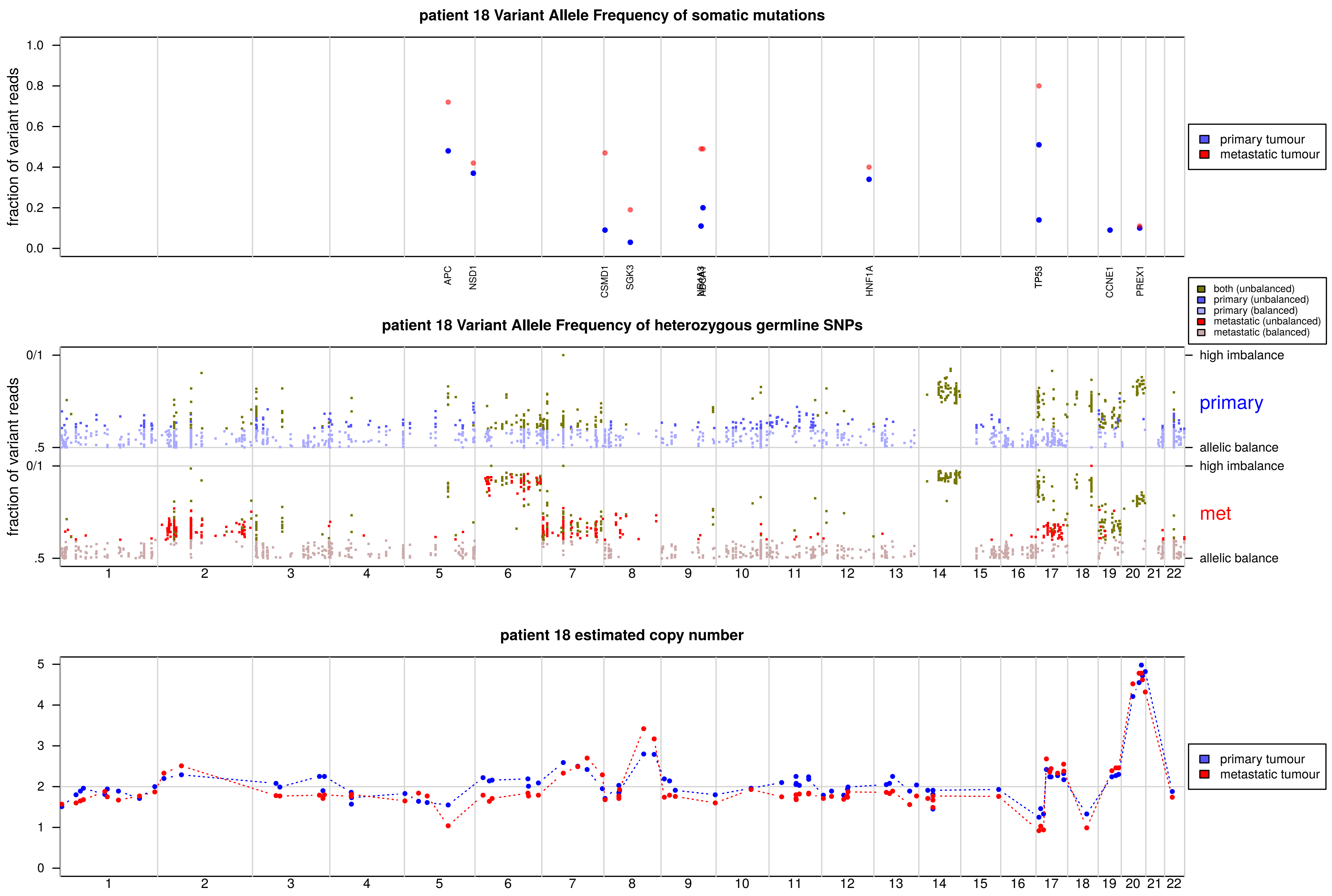
patient 18 Variant Allele Frequency of somatic mutations
fraction of variant reads
1.0
0.8
0.6
0.4
0.2
0.0
primary tumour
metastatic tumour
APC
NSD1
CSMD1
SGK3
HNF1A
TP53
CCNE1
PREX1
both (unbalanced)
primary (unbalanced)
primary (balanced)
metastatic (unbalanced)
metastatic (balanced)
patient 18 Variant Allele Frequency of heterozygous germline SNPs
fraction of variant reads
0/1
.5
0/1
.5
high imbalance
primary
allelic balance
high imbalance
met
allelic balance
1 2 3 4 5 6 7 8 9 10 11 12 13 14 15 16 17 18 19 20 21 22
patient 18 estimated copy number
5
4
3
2
1
0
primary tumour
metastatic tumour
1 2 3 4 5 6 7 8 9 10 11 12 13 14 15 16 17 18 19 20 21 22
