# Supplementary material for: High-depth sequencing of over 750 genes supports linear progression of primary tumors and metastases in most patients with liver-limited metastatic colorectal cancer
Source: Genome Biol. 2015 Feb 12;16(1):32. doi: 10.1186/s13059-015-0589-1 (PMC4365969; doi:10.1186/s13059-015-0589-1)
Supplement: Additional file 8: Table S6. — Description of Targeted NGS panel. List of 799 cancer-associated genes selected for targeted next generation sequencing. [file 13059_2015_589_MOESM8_ESM.pdf]

Supplementary Table 6. List of 799 cancer-associated genes selected for targeted next generation sequencing

Number	Gene symbol	Gene name
1	ABL1	c-abl oncogene 1, non-receptor tyrosine kinase
2	AKAP9	A kinase (PRKA) anchor protein (yotiao) 9
3	AKT1	v-akt murine thymoma viral oncogene homolog 1
4	AKT2	v-akt murine thymoma viral oncogene homolog 2
5	ALK	anaplastic lymphoma receptor tyrosine kinase
6	APC	adenomatous polyposis coli
7	ARID1A	AT rich interactive domain 1A (SWI-like)
8	ARID2	AT rich interactive domain 2 (ARID, RFX-like)
9	ARNT	aryl hydrocarbon receptor nuclear translocator
10	ASPSCR1	alveolar soft part sarcoma chromosome region, candidate 1
11	ASXL1	additional sex combs like 1 (Drosophila)
12	ATF1	activating transcription factor 1
13	ATM	ataxia telangiectasia mutated
14	ATRX	alpha thalassemia/mental retardation syndrome X-linked
15	BAP1	BRCA1 associated protein-1 (ubiquitin carboxy-terminal hydrolase)
16	BCL2	B-cell CLL/lymphoma 2
17	BCL6	B-cell CLL/lymphoma 6
18	BCOR	BCL6 corepressor
19	BCR	breakpoint cluster region
20	FLCN	folliculin
21	BLM	Bloom syndrome, RecQ helicase-like
22	BRAF	v-raf murine sarcoma viral oncogene homolog B1

23	BRCA1	breast cancer 1, early onset
24	BRCA2	breast cancer 2, early onset
25	BRD3	bromodomain containing 3
26	BRD4	bromodomain containing 4
27	BRIP1	BRCA1 interacting protein C-terminal helicase 1
28	RMI2	chromosome 16 open reading frame 75
29	CAMTA1	calmodulin binding transcription activator 1
30	CANT1	calcium activated nucleotidase 1
31	CARD11	caspase recruitment domain family, member 11
32	CARS	cysteinyl-tRNA synthetase
33	RUNX1T1	runt-related transcription factor 1; translocated to, 1 (cyclin D-related)
34	CBFA2T3	core-binding factor, runt domain, alpha subunit 2; translocated to, 3
35	CBFB	core-binding factor, beta subunit
36	CBL	Cas-Br-M (murine) ecotropic retroviral transforming sequence
37	CBLB	Cas-Br-M (murine) ecotropic retroviral transforming sequence b
38	CBLC	Cas-Br-M (murine) ecotropic retroviral transforming sequence c
39	CCDC6	coiled-coil domain containing 6
40	CCNB1IP1	cyclin B1 interacting protein 1
41	CCND1	cyclin D1
42	CCND2	cyclin D2
43	CCND3	cyclin D3
44	CCNE1	cyclin E1
45	PDCD1LG2	programmed cell death 1 ligand 2
46	CD274	CD274 molecule
47	CD74	CD74 molecule, major histocompatibility complex, class II invariant chain
48	CD79A	CD79a molecule, immunoglobulin-associated alpha
49	CD79B	CD79b molecule, immunoglobulin-associated beta

50	CDH1	cadherin 1, type 1, E-cadherin (epithelial)
51	CDH11	cadherin 11, type 2, OB-cadherin (osteoblast)
52	CDK12	cyclin-dependent kinase 12
53	CDK4	cyclin-dependent kinase 4
54	CDK6	cyclin-dependent kinase 6
55	CDKN2A	cyclin-dependent kinase inhibitor 2A (melanoma, p16, inhibits CDK4)
56	CDKN2C	cyclin-dependent kinase inhibitor 2C (p18, inhibits CDK4)
57	CDX2	caudal type homeobox 2
58	CEBPA	CCAAT/enhancer binding protein (C/EBP), alpha
59	CEP110	centrosomal protein 110kDa
60	CHCHD7	coiled-coil-helix-coiled-coil-helix domain containing 7
61	CHEK2	CHK2 checkpoint homolog (S. pombe)
62	CHIC2	cysteine-rich hydrophobic domain 2
63	CHN1	chimerin (chimaerin) 1
64	CIC	capicua homolog (Drosophila)
65	CIITA	class II, major histocompatibility complex, transactivator
66	CLTC	clathrin, heavy chain (Hc)
67	CLTCL1	clathrin, heavy chain-like 1
68	CXCR7	chemokine (C-X-C motif) receptor 7
69	COL1A1	collagen, type I, alpha 1
70	KLF6	Kruppel-like factor 6
71	COX6C	cytochrome c oxidase subunit VIc
72	CREB1	cAMP responsive element binding protein 1
73	CREB3L2	cAMP responsive element binding protein 3-like 2
74	CREBBP	CREB binding protein
75	CRLF2	cytokine receptor-like factor 2
76	CRTC3	CREB regulated transcription coactivator 3

77	CTNNB1	catenin (cadherin-associated protein), beta 1, 88kDa
78	CYLD	cylindromatosis (turban tumor syndrome)
79	DAXX	death-domain associated protein
80	DDB2	damage-specific DNA binding protein 2, 48kDa
81	DDIT3	DNA-damage-inducible transcript 3
82	DDX10	DEAD (Asp-Glu-Ala-Asp) box polypeptide 10
83	DDX5	DEAD (Asp-Glu-Ala-Asp) box polypeptide 5
84	DDX6	DEAD (Asp-Glu-Ala-Asp) box polypeptide 6
85	DEK	DEK oncogene
86	DICER1	dicer 1, ribonuclease type III
87	DNM2	dynamin 2
88	DNMT3A	DNA (cytosine-5-)-methyltransferase 3 alpha
89	EBF1	early B-cell factor 1
90	ECT2L	epithelial cell transforming sequence 2 oncogene-like
91	EGFR	epidermal growth factor receptor
92	EIF4A2	eukaryotic translation initiation factor 4A2
93	ELF4	E74-like factor 4 (ets domain transcription factor)
94	ELK4	ELK4, ETS-domain protein (SRF accessory protein 1)
95	ERC1	ELKS/RAB6-interacting/CAST family member 1
96	ELL	elongation factor RNA polymerase II
97	EML4	echinoderm microtubule associated protein like 4
98	EP300	E1A binding protein p300
99	EPS15	epidermal growth factor receptor pathway substrate 15
100	ERBB2	v-erb-b2 erythroblastic leukemia viral oncogene homolog 2, neuro/glioblastoma derived oncogene homolog (avian)
101	ERCC1	excision repair cross-complementing rodent repair deficiency, complementation group 1 (includes overlapping antisense sequence)
102	ERCC2	excision repair cross-complementing rodent repair deficiency, complementation group 2

103	ERCC3	excision repair cross-complementing rodent repair deficiency, complementation group 3 (xeroderma pigmentosum group B complementing)
104	ERCC4	excision repair cross-complementing rodent repair deficiency, complementation group 4
105	ERCC5	excision repair cross-complementing rodent repair deficiency, complementation group 5
106	ERG	v-ets erythroblastosis virus E26 oncogene homolog (avian)
107	ETV1	ets variant 1
108	ETV4	ets variant 4
109	ETV5	ets variant 5
110	ETV6	ets variant 6
111	MECOM	MDS1 and EVI1 complex locus
112	EWSR1	Ewing sarcoma breakpoint region 1
113	EZH2	enhancer of zeste homolog 2 (Drosophila)
114	EZR	ezrin
115	ACSL6	acyl-CoA synthetase long-chain family member 6
116	FAM46C	family with sequence similarity 46, member C
117	FANCA	Fanconi anemia, complementation group A
118	FANCC	Fanconi anemia, complementation group C
119	FANCD2	Fanconi anemia, complementation group D2
120	FANCE	Fanconi anemia, complementation group E
121	FANCF	Fanconi anemia, complementation group F
122	FANCG	Fanconi anemia, complementation group G
123	FBXO11	F-box protein 11
124	FBXW7	F-box and WD repeat domain containing 7
125	FCGR2B	Fc fragment of IgG, low affinity IIb, receptor (CD32)
126	FEV	FEV (ETS oncogene family)
127	FGFR1	fibroblast growth factor receptor 1
128	FGFR1OP	FGFR1 oncogene partner

129	FGFR2	fibroblast growth factor receptor 2
130	FGFR3	fibroblast growth factor receptor 3
131	FH	fumarate hydratase
132	FHIT	fragile histidine triad gene
133	FIP1L1	FIP1 like 1 (S. cerevisiae)
134	FLI1	Friend leukemia virus integration 1
135	FLT3	fms-related tyrosine kinase 3
136	FNBP1	formin binding protein 1
137	FOXL2	forkhead box L2
138	FOXO1	forkhead box O1
139	FOXO3	forkhead box O3
140	FOXP1	forkhead box P1
141	FSTL3	follistatin-like 3 (secreted glycoprotein)
142	FUBP1	far upstream element (FUSE) binding protein 1
143	FUS	fused in sarcoma
144	KDSR	3-ketodihydrosphingosine reductase
145	GAS7	growth arrest-specific 7
146	GATA1	GATA binding protein 1 (globin transcription factor 1)
147	GATA2	GATA binding protein 2
148	GATA3	GATA binding protein 3
149	GMPS	guanine monphosphate synthetase
150	GNA11	guanine nucleotide binding protein (G protein), alpha 11 (Gq class)
151	GNAQ	guanine nucleotide binding protein (G protein), q polypeptide
152	GNAS	GNAS complex locus
153	GOLGA5	golgin A5
154	GOPC	golgi-associated PDZ and coiled-coil motif containing
155	GPC3	glypican 3

156	GPHN	gephyrin
157	ARHGAP26	Rho GTPase activating protein 26
158	H3F3A	H3 histone, family 3A
159	SPECC1	sperm antigen with calponin homology and coiled-coil domains 1
160	CLP1	CLP1, cleavage and polyadenylation factor I subunit, homolog (S. cerevisiae)
161	HERPUD1	homocysteine-inducible, endoplasmic reticulum stress-inducible, ubiquitin-like domain member 1
162	HEY1	hairy/enhancer-of-split related with YRPW motif 1
163	HIP1	huntingtin interacting protein 1
164	HIST1H4I	histone cluster 1, H4i
165	HLF	hepatic leukemia factor
166	MNX1	motor neuron and pancreas homeobox 1
167	HMGA1	high mobility group AT-hook 1
168	HMGA2	high mobility group AT-hook 2
169	HNRNPA2B1	heterogeneous nuclear ribonucleoprotein A2/B1
170	HOOK3	hook homolog 3 (Drosophila)
171	HOXA11	homeobox A11
172	HOXA13	homeobox A13
173	HOXA9	homeobox A9
174	HOXC11	homeobox C11
175	HOXC13	homeobox C13
176	HOXD11	homeobox D11
177	HOXD13	homeobox D13
178	HRAS	v-Ha-ras Harvey rat sarcoma viral oncogene homolog
179	CDC73	cell division cycle 73, Paf1/RNA polymerase II complex component, homolog (S. cerevisiae)
180	HSP90AA1	heat shock protein 90kDa alpha (cytosolic), class A member 1
181	HSP90AB1	heat shock protein 90kDa alpha (cytosolic), class B member 1
182	IDH1	isocitrate dehydrogenase 1 (NADP+), soluble

183	IDH2	isocitrate dehydrogenase 2 (NADP+), mitochondrial
184	IKZF1	IKAROS family zinc finger 1 (Ikaros)
185	IL2	interleukin 2
186	IL21R	interleukin 21 receptor
187	IL6ST	interleukin 6 signal transducer (gp130, oncostatin M receptor)
188	IL7R	interleukin 7 receptor
189	IRF4	interferon regulatory factor 4
190	FCRL4	Fc receptor-like 4
191	ITK	IL2-inducible T-cell kinase
192	JAK1	Janus kinase 1
193	JAK2	Janus kinase 2
194	JAK3	Janus kinase 3
195	JAZF1	JAZF zinc finger 1
196	JUN	jun proto-oncogene
197	KDM5A	lysine (K)-specific demethylase 5A
198	KDM5C	lysine (K)-specific demethylase 5C
199	KDM6A	lysine (K)-specific demethylase 6A
200	KDR	kinase insert domain receptor (a type III receptor tyrosine kinase)
201	KIAA1549	KIAA1549
202	KIF5B	kinesin family member 5B
203	KIT	v-kit Hardy-Zuckerman 4 feline sarcoma viral oncogene homolog
204	KLK2	kallikrein-related peptidase 2
205	KRAS	v-Ki-ras2 Kirsten rat sarcoma viral oncogene homolog
206	KTN1	kinectin 1 (kinesin receptor)
207	AFF3	AF4/FMR2 family, member 3
208	LASP1	LIM and SH3 protein 1
209	LCK	lymphocyte-specific protein tyrosine kinase

210	LCP1	lymphocyte cytosolic protein 1 (L-plastin)
211	TET1	tet oncogene 1
212	LHFP	lipoma HMGIC fusion partner
213	LIFR	leukemia inhibitory factor receptor alpha
214	LMO1	LIM domain only 1 (rhombotin 1)
215	LMO2	LIM domain only 2 (rhombotin-like 1)
216	LPP	LIM domain containing preferred translocation partner in lipoma
217	LRIG3	leucine-rich repeats and immunoglobulin-like domains 3
218	LYL1	lymphoblastic leukemia derived sequence 1
219	MAF	v-maf musculoaponeurotic fibrosarcoma oncogene homolog (avian)
220	MAFB	v-maf musculoaponeurotic fibrosarcoma oncogene homolog B (avian)
221	MALT1	mucosa associated lymphoid tissue lymphoma translocation gene 1
222	MAML2	mastermind-like 2 (Drosophila)
223	MAP2K4	mitogen-activated protein kinase kinase 4
224	MDM2	Mdm2 p53 binding protein homolog (mouse)
225	MDM4	Mdm4 p53 binding protein homolog (mouse)
226	CRTC1	CREB regulated transcription coactivator 1
227	MED12	mediator complex subunit 12
228	MEN1	multiple endocrine neoplasia I
229	MET	met proto-oncogene (hepatocyte growth factor receptor)
230	MITF	microphthalmia-associated transcription factor
231	MKL1	megakaryoblastic leukemia (translocation) 1
232	MLF1	myeloid leukemia factor 1
233	MLH1	mutL homolog 1, colon cancer, nonpolyposis type 2 (E. coli)
234	MLL	myeloid/lymphoid or mixed-lineage leukemia (trithorax homolog, Drosophila)
235	MLL2	myeloid/lymphoid or mixed-lineage leukemia 2
236	MLL3	myeloid/lymphoid or mixed-lineage leukemia 3

237	MLLT1	myeloid/lymphoid or mixed-lineage leukemia (trithorax homolog, Drosophila); translocated to, 1
238	MLLT10	myeloid/lymphoid or mixed-lineage leukemia (trithorax homolog, Drosophila); translocated to, 10
239	AFF1	AF4/FMR2 family, member 1
240	MLLT3	myeloid/lymphoid or mixed-lineage leukemia (trithorax homolog, Drosophila); translocated to, 3
241	MLLT4	myeloid/lymphoid or mixed-lineage leukemia (trithorax homolog, Drosophila); translocated to, 4
242	MLLT6	myeloid/lymphoid or mixed-lineage leukemia (trithorax homolog, Drosophila); translocated to, 6
243	FOXO4	forkhead box O4
244	MN1	meningioma (disrupted in balanced translocation) 1
245	MPL	myeloproliferative leukemia virus oncogene
246	SEPT9	septin 9
247	MSH2	mutS homolog 2, colon cancer, nonpolyposis type 1 (E. coli)
248	MSH6	mutS homolog 6 (E. coli)
249	MSI2	musashi homolog 2 (Drosophila)
250	MSN	moesin
251	MTCP1	mature T-cell proliferation 1
252	MUC1	mucin 1, cell surface associated
253	MUTYH	mutY homolog (E. coli)
254	MYB	v-myb myeloblastosis viral oncogene homolog (avian)
255	MYC	v-myc myelocytomatosis viral oncogene homolog (avian)
256	MYCL1	v-myc myelocytomatosis viral oncogene homolog 1, lung carcinoma derived (avian)
257	MYCN	v-myc myelocytomatosis viral related oncogene, neuroblastoma derived (avian)
258	MYH11	myosin, heavy chain 11, smooth muscle
259	MYH9	myosin, heavy chain 9, non-muscle
260	KAT6B	MYST histone acetyltransferase (monocytic leukemia) 4
261	NACA	nascent polypeptide-associated complex alpha subunit
262	NBN	nibrin
263	NCOA1	nuclear receptor coactivator 1

264	NCOA2	nuclear receptor coactivator 2
265	NCOA4	nuclear receptor coactivator 4
266	NDRG1	N-myc downstream regulated 1
267	NF1	neurofibromin 1
268	NF2	neurofibromin 2 (merlin)
269	NFE2L2	nuclear factor (erythroid-derived 2)-like 2
270	NFIB	nuclear factor I/B
271	NFKB2	nuclear factor of kappa light polypeptide gene enhancer in B-cells 2 (p49/p100)
272	NIN	ninein (GSK3B interacting protein)
273	NKX2-1	NK2 homeobox 1
274	NONO	non-POU domain containing, octamer-binding
275	NOTCH1	notch 1
276	NOTCH2	notch 2
277	NPM1	nucleophosmin (nucleolar phosphoprotein B23, numatrin)
278	NR4A3	nuclear receptor subfamily 4, group A, member 3
279	NRAS	neuroblastoma RAS viral (v-ras) oncogene homolog
280	NSD1	nuclear receptor binding SET domain protein 1
281	NTRK1	neurotrophic tyrosine kinase, receptor, type 1
282	NTRK3	neurotrophic tyrosine kinase, receptor, type 3
283	NUMA1	nuclear mitotic apparatus protein 1
284	NUP214	nucleoporin 214kDa
285	NUP98	nucleoporin 98kDa
286	OLIG2	oligodendrocyte lineage transcription factor 2
287	OMD	osteomodulin
288	P2RY8	purinergic receptor P2Y, G-protein coupled, 8
289	PAFAH1B2	platelet-activating factor acetylhydrolase 1b, catalytic subunit 2 (30kDa)
290	PALB2	partner and localizer of BRCA2

291	PAX3	paired box 3
292	PAX5	paired box 5
293	PAX7	paired box 7
294	PAX8	paired box 8
295	PBRM1	polybromo 1
296	PBX1	pre-B-cell leukemia homeobox 1
297	PCM1	pericentriolar material 1
298	PCSK7	proprotein convertase subtilisin/kexin type 7
299	PDE4DIP	phosphodiesterase 4D interacting protein
300	PDGFB	platelet-derived growth factor beta polypeptide (simian sarcoma viral (v-sis) oncogene homolog)
301	PDGFRA	platelet-derived growth factor receptor, alpha polypeptide
302	PDGFRB	platelet-derived growth factor receptor, beta polypeptide
303	PER1	period homolog 1 (Drosophila)
304	PHF6	PHD finger protein 6
305	PHOX2B	paired-like homeobox 2b
306	PICALM	phosphatidylinositol binding clathrin assembly protein
307	PIK3CA	phosphoinositide-3-kinase, catalytic, alpha polypeptide
308	PIK3R1	phosphoinositide-3-kinase, regulatory subunit 1 (alpha)
309	PIM1	pim-1 oncogene
310	PLAG1	pleiomorphic adenoma gene 1
311	PML	promyelocytic leukemia
312	PMS1	PMS1 postmeiotic segregation increased 1 (S. cerevisiae)
313	PMS2	PMS2 postmeiotic segregation increased 2 (S. cerevisiae)
314	PRRX1	paired related homeobox 1
315	SEPT5	septin 5
316	POU2AF1	POU class 2 associating factor 1
317	POU5F1	POU class 5 homeobox 1

318	PPARG	peroxisome proliferator-activated receptor gamma
319	PPP2R1A	protein phosphatase 2, regulatory subunit A, alpha
320	PRCC	papillary renal cell carcinoma (translocation-associated)
321	PRDM1	PR domain containing 1, with ZNF domain
322	PRDM16	PR domain containing 16
323	PRF1	perforin 1 (pore forming protein)
324	PRKAR1A	protein kinase, cAMP-dependent, regulatory, type I, alpha (tissue specific extinguisher 1)
325	MALAT1	metastasis associated lung adenocarcinoma transcript 1 (non-protein coding)
326	PSIP1	PC4 and SFRS1 interacting protein 1
327	PTCH1	patched 1
328	PTEN	phosphatase and tensin homolog
329	PTPN11	protein tyrosine phosphatase, non-receptor type 11
330	RABEP1	rabaptin, RAB GTPase binding effector protein 1
331	RAD51B	RAD51-like 1 (S. cerevisiae)
332	RAF1	v-raf-1 murine leukemia viral oncogene homolog 1
333	RALGDS	ral guanine nucleotide dissociation stimulator
334	RANBP17	RAN binding protein 17
335	RAP1GDS1	RAP1, GTP-GDP dissociation stimulator 1
336	RARA	retinoic acid receptor, alpha
337	RB1	retinoblastoma 1
338	RBM15	RNA binding motif protein 15
339	RECQL4	RecQ protein-like 4
340	REL	v-rel reticuloendotheliosis viral oncogene homolog (avian)
341	RET	ret proto-oncogene
342	ROS1	c-ros oncogene 1 , receptor tyrosine kinase
343	RPL22	ribosomal protein L22
344	RPN1	ribophorin I

345	SNX29	RUN domain containing 2A
346	RUNX1	runt-related transcription factor 1
347	KAT6A	K(lysine) acetyltransferase 6A
348	SBDS	Shwachman-Bodian-Diamond syndrome
349	SDC4	syndecan 4
350	SDHAF2	succinate dehydrogenase complex assembly factor 2
351	SDHB	succinate dehydrogenase complex, subunit B, iron sulfur (Ip)
352	SDHC	succinate dehydrogenase complex, subunit C, integral membrane protein, 15kDa
353	SDHD	succinate dehydrogenase complex, subunit D, integral membrane protein
354	SEPT6	septin 6
355	SET	SET nuclear oncogene
356	SETD2	SET domain containing 2
357	SF3B1	splicing factor 3b, subunit 1, 155kDa
358	SFPQ	splicing factor proline/glutamine-rich
359	SRSF3	serine/arginine-rich splicing factor 3
360	SH3GL1	SH3-domain GRB2-like 1
361	STIL	SCL/TAL1 interrupting locus
362	SLC34A2	solute carrier family 34 (sodium phosphate), member 2
363	SLC45A3	solute carrier family 45, member 3
364	SMARCA4	SWI/SNF related, matrix associated, actin dependent regulator of chromatin, subfamily a, member 4
365	SMARCB1	SWI/SNF related, matrix associated, actin dependent regulator of chromatin, subfamily b, member 1
366	SMO	smoothened homolog (Drosophila)
367	SOCS1	suppressor of cytokine signaling 1
368	SOX2	SRY (sex determining region Y)-box 2
369	SRGAP3	SLIT-ROBO Rho GTPase activating protein 3
370	SRSF2	serine/arginine-rich splicing factor 2
371	SS18	synovial sarcoma translocation, chromosome 18

372	SS18L1	synovial sarcoma translocation gene on chromosome 18-like 1
373	ABI1	abl-interactor 1
374	SSX1	synovial sarcoma, X breakpoint 1
375	SSX2	synovial sarcoma, X breakpoint 2
376	SSX4	synovial sarcoma, X breakpoint 4
377	STK11	serine/threonine kinase 11
378	STL	six-twelve leukemia
379	SUFU	suppressor of fused homolog (Drosophila)
380	SUZ12	suppressor of zeste 12 homolog (Drosophila)
381	SYK	spleen tyrosine kinase
382	TAF15	TAF15 RNA polymerase II, TATA box binding protein (TBP)-associated factor, 68kDa
383	TAL1	T-cell acute lymphocytic leukemia 1
384	TAL2	T-cell acute lymphocytic leukemia 2
385	TCEA1	transcription elongation factor A (SII), 1
386	HNF1A	HNF1 homeobox A
387	TCF12	transcription factor 12
388	TCF3	transcription factor 3 (E2A immunoglobulin enhancer binding factors E12/E47)
389	TCF7L2	transcription factor 7-like 2 (T-cell specific, HMG-box)
390	TCL1A	T-cell leukemia/lymphoma 1A
391	TCL6	T-cell leukemia/lymphoma 6 (non-protein coding)
392	TET2	tet oncogene family member 2
393	TFE3	transcription factor binding to IGHM enhancer 3
394	TFEB	transcription factor EB
395	TFG	TRK-fused gene
396	TFPT	TCF3 (E2A) fusion partner (in childhood Leukemia)
397	TFRC	transferrin receptor (p90, CD71)
398	THRAP3	thyroid hormone receptor associated protein 3

399	TRIM24	tripartite motif-containing 24
400	TLX1	T-cell leukemia homeobox 1
401	TLX3	T-cell leukemia homeobox 3
402	TMPRSS2	transmembrane protease, serine 2
403	TNFAIP3	tumor necrosis factor, alpha-induced protein 3
404	TNFRSF14	tumor necrosis factor receptor superfamily, member 14 (herpesvirus entry mediator)
405	TNFRSF17	tumor necrosis factor receptor superfamily, member 17
406	FAS	Fas (TNF receptor superfamily, member 6)
407	TOP1	topoisomerase (DNA) I
408	TP53	tumor protein p53
409	TPM3	tropomyosin 3
410	TPM4	tropomyosin 4
411	TPR	translocated promoter region (to activated MET oncogene)
412	TRIM27	tripartite motif-containing 27
413	TRIM33	tripartite motif-containing 33
414	TRIP11	thyroid hormone receptor interactor 11
415	TSC1	tuberous sclerosis 1
416	TSC2	tuberous sclerosis 2
417	TSHR	thyroid stimulating hormone receptor
418	TTL	tubulin tyrosine ligase
419	U2AF1	U2 small nuclear RNA auxiliary factor 1
420	USP6	ubiquitin specific peptidase 6 (Tre-2 oncogene)
421	VHL	von Hippel-Lindau tumor suppressor
422	VTI1A	vesicle transport through interaction with t-SNAREs homolog 1A (yeast)
423	WAS	Wiskott-Aldrich syndrome (eczema-thrombocytopenia)
424	WHSC1	Wolf-Hirschhorn syndrome candidate 1
425	WHSC1L1	Wolf-Hirschhorn syndrome candidate 1-like 1

426	WIF1	WNT inhibitory factor 1
427	WRN	Werner syndrome, RecQ helicase-like
428	WT1	Wilms tumor 1
429	AMER1	family with sequence similarity 123B
430	WWTR1	WW domain containing transcription regulator 1
431	XPA	xeroderma pigmentosum, complementation group A
432	XPC	xeroderma pigmentosum, complementation group C
433	XPO1	exportin 1 (CRM1 homolog, yeast)
434	YWHAE	tyrosine 3-monooxygenase/tryptophan 5-monooxygenase activation protein, epsilon polypeptide
435	ZBTB16	zinc finger and BTB domain containing 16
436	ZMYM2	zinc finger, MYM-type 2
437	PATZ1	POZ (BTB) and AT hook containing zinc finger 1
438	ZNF331	zinc finger protein 331
439	ZNF384	zinc finger protein 384
440	ZNF521	zinc finger protein 521
441	CNBP	CCHC-type zinc finger, nucleic acid binding protein
442	ZRSR2	zinc finger (CCCH type), RNA-binding motif and serine/arginine rich 2
443	NCOR1	nuclear receptor corepressor 1
444	ZNF703	zinc finger protein 703
445	ZNF217	zinc finger protein 217
446	ROBO2	roundabout, axon guidance receptor, homolog 2 (Drosophila)
447	RNF43	ring finger protein 43
448	PEG3	paternally expressed 3
449	XIRP2	xin actin-binding repeat containing 2
450	PCDHA13	protocadherin alpha 13
451	NDC80	NDC80 homolog, kinetochore complex component (S. cerevisiae)
452	RADIL	Ras association and DIL domains

453	LAMA2	laminin, alpha 2
454	FAT4	FAT tumor suppressor homolog 4 (Drosophila)
455	SMAD4	SMAD family member 4
456	EPHA2	EPH receptor A2
457	EPHA5	EPH receptor A5
458	EPHA6	EPH receptor A6
459	EPHA7	EPH receptor A7
460	EPHB6	EPH receptor B6
461	EPHA3	EPH receptor A3
462	PRKDC	protein kinase, DNA-activated, catalytic polypeptide
463	CHEK1	CHK1 checkpoint homolog (S. pombe)
464	DDR2	discoidin domain receptor tyrosine kinase 2
465	GPR124	G protein-coupled receptor 124
466	SMAD3	SMAD family member 3
467	AURKA	aurora kinase A
468	IGF1R	insulin-like growth factor 1 receptor
469	TNKS2	tankyrase, TRF1-interacting ankyrin-related ADP-ribose polymerase 2
470	SMAD2	SMAD family member 2
471	EPHB1	EPH receptor B1
472	ATR	ataxia telangiectasia and Rad3 related
473	TGFBR2	transforming growth factor, beta receptor II (70/80kDa)
474	SRC	v-src sarcoma (Schmidt-Ruppin A-2) viral oncogene homolog (avian)
475	MMP2	matrix metallopeptidase 2 (gelatinase A, 72kDa gelatinase, 72kDa type IV collagenase)
476	PTPRT	protein tyrosine phosphatase, receptor type, T
477	UBR5	ubiquitin protein ligase E3 component n-recognin 5
478	GLI1	GLI family zinc finger 1
479	TRRAP	transformation/transcription domain-associated protein

480	CENPF	centromere protein F, 350/400kDa (mitosin)
481	CNTN1	contactin 1
482	CTSH	cathepsin H
483	DLC1	deleted in liver cancer 1
484	DYRK2	dual-specificity tyrosine-(Y)-phosphorylation regulated kinase 2
485	ICK	intestinal cell (MAK-like) kinase
486	IKBKB	inhibitor of kappa light polypeptide gene enhancer in B-cells, kinase beta
487	ING4	inhibitor of growth family, member 4
488	LATS2	LATS, large tumor suppressor, homolog 2 (Drosophila)
489	MAPK13	mitogen-activated protein kinase 13
490	MARK4	MAP/microtubule affinity-regulating kinase 4
491	MMP16	matrix metallopeptidase 16 (membrane-inserted)
492	PARP1	poly (ADP-ribose) polymerase 1
493	PDK3	pyruvate dehydrogenase kinase, isozyme 3
494	PIM2	pim-2 oncogene
495	PTPN9	protein tyrosine phosphatase, non-receptor type 9
496	RAD21	RAD21 homolog (S. pombe)
497	RAD54B	RAD54 homolog B (S. cerevisiae)
498	RGL2	ral guanine nucleotide dissociation stimulator-like 2
499	SGK3	serum/glucocorticoid regulated kinase family, member 3
500	SOX11	SRY (sex determining region Y)-box 11
501	TYK2	tyrosine kinase 2
502	CHD8	chromodomain helicase DNA binding protein 8
503	CLSPN	claspin
504	CTNND2	catenin (cadherin-associated protein), delta 2 (neural plakophilin-related arm-repeat protein)
505	EP400	E1A binding protein p400
506	FYN	FYN oncogene related to SRC, FGR, YES

507	MACF1	microtubule-actin crosslinking factor 1
508	MAP3K2	mitogen-activated protein kinase kinase kinase 2
509	NEK8	NIMA (never in mitosis gene a)- related kinase 8
510	SIK1	salt-inducible kinase 1
511	TOP2B	topoisomerase (DNA) II beta 180kDa
512	NEK11	NIMA (never in mitosis gene a)- related kinase 11
513	PRKCA	protein kinase C, alpha
514	PRKD2	protein kinase D2
515	AXL	AXL receptor tyrosine kinase
516	GLI3	GLI family zinc finger 3
517	MAP3K6	mitogen-activated protein kinase kinase kinase 6
518	PAK7	p21 protein (Cdc42/Rac)-activated kinase 7
519	SPEN	spen homolog, transcriptional regulator (Drosophila)
520	MTOR	mechanistic target of rapamycin (serine/threonine kinase)
521	LTBP1	latent transforming growth factor beta binding protein 1
522	MAP4K4	mitogen-activated protein kinase kinase kinase kinase 4
523	HIF1A	hypoxia inducible factor 1, alpha subunit (basic helix-loop-helix transcription factor)
524	MINK1	misshapen-like kinase 1
525	ROCK1	Rho-associated, coiled-coil containing protein kinase 1
526	ROCK2	Rho-associated, coiled-coil containing protein kinase 2
527	N4BP2	NEDD4 binding protein 2
528	NLRP1	NLR family, pyrin domain containing 1
529	RPS6KA2	ribosomal protein S6 kinase, 90kDa, polypeptide 2
530	ADAMTS18	ADAM metallopeptidase with thrombospondin type 1 motif, 18
531	TNK2	tyrosine kinase, non-receptor, 2
532	PREX2	phosphatidylinositol-3,4,5-trisphosphate-dependent Rac exchange factor 2
533	WNK2	WNK lysine deficient protein kinase 2

534	CTNNA1	catenin (cadherin-associated protein), alpha 1, 102kDa
535	CYB5D2	cytochrome b5 domain containing 2
536	TNFRSF8	tumor necrosis factor receptor superfamily, member 8
537	NFKB1	nuclear factor of kappa light polypeptide gene enhancer in B-cells 1
538	RAD50	RAD50 homolog (S. cerevisiae)
539	XRCC6	X-ray repair complementing defective repair in Chinese hamster cells 6
540	HDAC4	histone deacetylase 4
541	ERCC6	excision repair cross-complementing rodent repair deficiency, complementation group 6
542	MAP2K7	mitogen-activated protein kinase kinase 7
543	CDC7	cell division cycle 7 homolog (S. cerevisiae)
544	ACVR1B	activin A receptor, type IB
545	CHUK	conserved helix-loop-helix ubiquitous kinase
546	TTK	TTK protein kinase
547	CSF1R	colony stimulating factor 1 receptor
548	MLL4	myeloid/lymphoid or mixed-lineage leukemia 4
549	SENP5	SUMO1/sentrin specific peptidase 5
550	TERT	telomerase reverse transcriptase
551	FN1	fibronectin 1
552	RPS6KA3	ribosomal protein S6 kinase, 90kDa, polypeptide 3
553	IRF2	interferon regulatory factor 2
554	SMARCA1	SWI/SNF related, matrix associated, actin dependent regulator of chromatin, subfamily a, member 1
555	SMARCD1	SWI/SNF related, matrix associated, actin dependent regulator of chromatin, subfamily d, member 1
556	ARID1B	AT rich interactive domain 1B (SWI1-like)
557	BAZ2B	bromodomain adjacent to zinc finger domain, 2B
558	BRD8	bromodomain containing 8
559	BPTF	bromodomain PHD finger transcription factor
560	BRE	brain and reproductive organ-expressed (TNFRSF1A modulator)

561	HIST1H4B	histone cluster 1, H4b
562	PARK2	parkinson protein 2, E3 ubiquitin protein ligase (parkin)
563	MAGI3	membrane associated guanylate kinase, WW and PDZ domain containing 3
564	AKT3	v-akt murine thymoma viral oncogene homolog 3 (protein kinase B, gamma)
565	MAP3K1	mitogen-activated protein kinase kinase kinase 1
566	GRM8	glutamate receptor, metabotropic 8
567	BAI3	brain-specific angiogenesis inhibitor 3
568	KEAP1	kelch-like ECH-associated protein 1
569	CHD6	chromodomain helicase DNA binding protein 6
570	RSF1	remodeling and spacing factor 1
571	ABL2	v-abl Abelson murine leukemia viral oncogene homolog 2
572	BMPR1A	bone morphogenetic protein receptor, type IA
573	BUB1B	budding uninhibited by benzimidazoles 1 homolog beta (yeast)
574	NEK10	NIMA (never in mitosis gene a)- related kinase 10
575	CASK	calcium/calmodulin-dependent serine protein kinase (MAGUK family)
576	CDC42BPA	CDC42 binding protein kinase alpha (DMPK-like)
577	NEK9	NIMA (never in mitosis gene a)- related kinase 9
578	SRPK2	SRSF protein kinase 2
579	CDKL2	cyclin-dependent kinase-like 2 (CDC2-related kinase)
580	MYLK2	myosin light chain kinase 2
581	CDC42BPB	CDC42 binding protein kinase beta (DMPK-like)
582	BRD2	bromodomain containing 2
583	GUCY2F	guanylate cyclase 2F, retinal
584	SMG1	SMG1 homolog, phosphatidylinositol 3-kinase-related kinase (C. elegans)
585	ERN1	endoplasmic reticulum to nucleus signaling 1
586	ERN2	endoplasmic reticulum to nucleus signaling 2
587	LRRK2	leucine-rich repeat kinase 2

588	MAST4	microtubule associated serine/threonine kinase family member 4
589	STK19	serine/threonine kinase 19
590	MGC42105	serine/threonine-protein kinase NIM1
591	ULK2	unc-51-like kinase 2 (C. elegans)
592	AATK	apoptosis-associated tyrosine kinase
593	ANKK1	ankyrin repeat and kinase domain containing 1
594	ARAF	v-raf murine sarcoma 3611 viral oncogene homolog
595	BMPR1B	bone morphogenetic protein receptor, type IB
596	BRDT	bromodomain, testis-specific
597	BRSK1	BR serine/threonine kinase 1
598	CIT	citron (rho-interacting, serine/threonine kinase 21)
599	DYRK1B	dual-specificity tyrosine-(Y)-phosphorylation regulated kinase 1B
600	EIF2AK4	eukaryotic translation initiation factor 2 alpha kinase 4
601	EPHA4	EPH receptor A4
602	EPHA8	EPH receptor A8
603	EPHB3	EPH receptor B3
604	EPHB4	EPH receptor B4
605	ERBB3	v-erb-b2 erythroblastic leukemia viral oncogene homolog 3 (avian)
606	ERBB4	v-erb-a erythroblastic leukemia viral oncogene homolog 4 (avian)
607	FASTK	Fas-activated serine/threonine kinase
608	FGFR4	fibroblast growth factor receptor 4
609	FLT1	fms-related tyrosine kinase 1 (vascular endothelial growth factor/vascular permeability factor receptor)
610	FLT4	fms-related tyrosine kinase 4
611	GAK	cyclin G associated kinase
612	INSR	insulin receptor
613	INSRR	insulin receptor-related receptor
614	IRAK1	interleukin-1 receptor-associated kinase 1

615	MAP2K1	mitogen-activated protein kinase kinase 1
616	MAP2K2	mitogen-activated protein kinase kinase 2
617	MAP2K3	mitogen-activated protein kinase kinase 3
618	MAP2K5	mitogen-activated protein kinase kinase 5
619	MAP2K6	mitogen-activated protein kinase kinase 6
620	MAP3K10	mitogen-activated protein kinase kinase kinase 10
621	MAP3K11	mitogen-activated protein kinase kinase kinase 11
622	MAP3K12	mitogen-activated protein kinase kinase kinase 12
623	MAP3K13	mitogen-activated protein kinase kinase kinase 13
624	MAP3K14	mitogen-activated protein kinase kinase kinase 14
625	MAP3K3	mitogen-activated protein kinase kinase kinase 3
626	MAP3K4	mitogen-activated protein kinase kinase kinase 4
627	MAP3K5	mitogen-activated protein kinase kinase kinase 5
628	MAP3K7	mitogen-activated protein kinase kinase kinase 7
629	MAP3K8	mitogen-activated protein kinase kinase kinase 8
630	MAP3K9	mitogen-activated protein kinase kinase kinase 9
631	MAP4K1	mitogen-activated protein kinase kinase kinase kinase 1
632	MAP4K2	mitogen-activated protein kinase kinase kinase kinase 2
633	MAP4K3	mitogen-activated protein kinase kinase kinase kinase 3
634	MAP4K5	mitogen-activated protein kinase kinase kinase kinase 5
635	MAPK1	mitogen-activated protein kinase 1
636	MAPK10	mitogen-activated protein kinase 10
637	MAPK11	mitogen-activated protein kinase 11
638	MAPK12	mitogen-activated protein kinase 12
639	MAPK3	mitogen-activated protein kinase 3
640	MAPK4	mitogen-activated protein kinase 4
641	MAPK6	mitogen-activated protein kinase 6

642	MAPK7	mitogen-activated protein kinase 7
643	MAPK9	mitogen-activated protein kinase 9
644	MAPKAPK2	mitogen-activated protein kinase-activated protein kinase 2
645	MAPKAPK3	mitogen-activated protein kinase-activated protein kinase 3
646	MAPKAPK5	mitogen-activated protein kinase-activated protein kinase 5
647	MAST1	microtubule associated serine/threonine kinase 1
648	MELK	maternal embryonic leucine zipper kinase
649	MERTK	c-mer proto-oncogene tyrosine kinase
650	MYO3A	myosin IIIA
651	MYO3B	myosin IIIB
652	NEK6	NIMA (never in mitosis gene a)-related kinase 6
653	NPR1	natriuretic peptide receptor A/guanylate cyclase A (atrionatriuretic peptide receptor A)
654	RPS6KC1	ribosomal protein S6 kinase, 52kDa, polypeptide 1
655	SCYL2	SCY1-like 2 (S. cerevisiae)
656	SGK2	serum/glucocorticoid regulated kinase 2
657	SLK	STE20-like kinase
658	PLK2	polo-like kinase 2
659	SNRK	SNF related kinase
660	STK3	serine/threonine kinase 3
661	STK32B	serine/threonine kinase 32B
662	STK33	serine/threonine kinase 33
663	STK36	serine/threonine kinase 36
664	STK38L	serine/threonine kinase 38 like
665	TAF1	TAF1 RNA polymerase II, TATA box binding protein (TBP)-associated factor, 250kDa
666	TAOK1	TAO kinase 1
667	TEC	tec protein tyrosine kinase
668	TEK	TEK tyrosine kinase, endothelial

669	TESK1	testis-specific kinase 1
670	TEX14	testis expressed 14
671	TGFBR1	transforming growth factor, beta receptor 1
672	TIE1	tyrosine kinase with immunoglobulin-like and EGF-like domains 1
673	TRIO	triple functional domain (PTPRF interacting)
674	WEE1	WEE1 homolog (S. pombe)
675	ZAP70	zeta-chain (TCR) associated protein kinase 70kDa
676	CSMD3	CUB and Sushi multiple domains 3
677	RHOA	ras homolog gene family, member A
678	RHOB	ras homolog gene family, member B
679	RHOC	ras homolog gene family, member C
680	BCL3	B-cell CLL/lymphoma 3
681	BMI1	BMI1 polycomb ring finger oncogene
682	CDKN1A	cyclin-dependent kinase inhibitor 1A (p21, Cip1)
683	CDKN1C	cyclin-dependent kinase inhibitor 1C (p57, Kip2)
684	CDKN2B	cyclin-dependent kinase inhibitor 2B (p15, inhibits CDK4)
685	CRK	v-crk sarcoma virus CT10 oncogene homolog (avian)
686	CRKL	v-crk sarcoma virus CT10 oncogene homolog (avian)-like
687	E2F1	E2F transcription factor 1
688	ELK1	ELK1, member of ETS oncogene family
689	CTTN	cortactin
690	EPHA1	EPH receptor A1
691	FES	feline sarcoma oncogene
692	FGF3	fibroblast growth factor 3
693	FGF4	fibroblast growth factor 4
694	FGR	Gardner-Rasheed feline sarcoma viral (v-fgr) oncogene homolog
695	FOXG1	forkhead box G1

696	FOS	FBJ murine osteosarcoma viral oncogene homolog
697	FOSL1	FOS-like antigen 1
698	FOSL2	FOS-like antigen 2
699	GLI2	GLI family zinc finger 2
700	JUNB	jun B proto-oncogene
701	JUND	jun D proto-oncogene
702	CD82	CD82 molecule
703	LYN	v-yes-1 Yamaguchi sarcoma viral related oncogene homolog
704	EPCAM	epithelial cell adhesion molecule
705	RALA	v-ral simian leukemia viral oncogene homolog A (ras related)
706	TIAM1	T-cell lymphoma invasion and metastasis 1
707	TNF	tumor necrosis factor
708	TP73	tumor protein p73
709	WNT2	wingless-type MMTV integration site family member 2
710	WNT5A	wingless-type MMTV integration site family, member 5A
711	WWOX	WW domain containing oxidoreductase
712	PTPRD	protein tyrosine phosphatase, receptor type, D
713	APAF1	apoptotic peptidase activating factor 1
714	GAB2	GRB2-associated binding protein 2
715	TWIST1	twist homolog 1 (Drosophila)
716	MAD1L1	MAD1 mitotic arrest deficient-like 1 (yeast)
717	GSK3B	glycogen synthase kinase 3 beta
718	SMARCA2	SWI/SNF related, matrix associated, actin dependent regulator of chromatin, subfamily a, member 2
719	SMARCA5	SWI/SNF related, matrix associated, actin dependent regulator of chromatin, subfamily a, member 5
720	SMARCC1	SWI/SNF related, matrix associated, actin dependent regulator of chromatin, subfamily c, member 1
721	MAPK14	mitogen-activated protein kinase 14
722	PAX2	paired box 2

723	DDR1	discoidin domain receptor tyrosine kinase 1
724	ARID4A	AT rich interactive domain 4A (RBP1-like)
725	ARID4B	AT rich interactive domain 4B (RBP1-like)
726	ARID5B	AT rich interactive domain 5B (MRF1-like)
727	PRDM2	PR domain containing 2, with ZNF domain
728	PIK3CB	phosphoinositide-3-kinase, catalytic, beta polypeptide
729	PIK3CD	phosphoinositide-3-kinase, catalytic, delta polypeptide
730	PIK3R2	phosphoinositide-3-kinase, regulatory subunit 2 (beta)
731	PIK3R3	phosphoinositide-3-kinase, regulatory subunit 3 (gamma)
732	PIK3R4	phosphoinositide-3-kinase, regulatory subunit 4
733	PREX1	phosphatidylinositol-3,4,5-trisphosphate-dependent Rac exchange factor 1
734	PTPRA	protein tyrosine phosphatase, receptor type, A
735	PTPRE	protein tyrosine phosphatase, receptor type, E
736	PTPRJ	protein tyrosine phosphatase, receptor type, J
737	SIRT1	sirtuin 1
738	KDM5B	lysine (K)-specific demethylase 5B
739	ACVR2A	activin A receptor, type IIA
740	CASP8	caspase 8, apoptosis-related cysteine peptidase
741	CDC27	cell division cycle 27 homolog (S. cerevisiae)
742	EDNRB	endothelin receptor type B
743	FZD3	frizzled homolog 3 (Drosophila)
744	GPC6	glypican 6
745	KIAA1804	mixed lineage kinase 4
746	MAP7	microtubule-associated protein 7
747	MIER3	mesoderm induction early response 1, family member 3
748	MSH3	mutS homolog 3 (E. coli)
749	MYO1B	myosin IB

750	PTPN12	protein tyrosine phosphatase, non-receptor type 12
751	SLC9A9	solute carrier family 9 (sodium/hydrogen exchanger), member 9
752	SOX9	SRY (sex determining region Y)-box 9
753	TCERG1	transcription elongation regulator 1
754	NAV2	neuron navigator 2
755	TCF7L1	transcription factor 7-like 1 (T-cell specific, HMG-box)
756	RBFOX1	RNA binding protein, fox-1 homolog (C. elegans) 1
757	KLF5	Kruppel-like factor 5 (intestinal)
758	FZD10	frizzled homolog 10 (Drosophila)
759	SCN5A	sodium channel, voltage-gated, type V, alpha subunit
760	RSPO2	R-spondin 2 homolog (Xenopus laevis)
761	RSPO3	R-spondin 3 homolog (Xenopus laevis)
762	POLE	polymerase (DNA directed), epsilon
763	FAT1	FAT tumor suppressor homolog 1 (Drosophila)
764	ZNRF3	zinc and ring finger 3
765	DCC	deleted in colorectal carcinoma
766	TARBP2	TAR (HIV-1) RNA binding protein 2
767	YAP1	Yes-associated protein 1
768	TBX5	T-box 5
769	BIRC5	baculoviral IAP repeat-containing 5
770	BCL2L1	BCL2-like 1
771	POLD1	polymerase (DNA directed), delta 1, catalytic subunit 125kDa
772	DCLK1	doublecortin-like kinase 1
773	IL23R	interleukin 23 receptor
774	PPM1L	protein phosphatase, Mg2+/Mn2+ dependent, 1L
775	SALL2	sal-like 2 (Drosophila)
776	RAB2B	RAB2B, member RAS oncogene family

777	ADAM9	ADAM metallopeptidase domain 9
778	ABCA1	ATP-binding cassette, sub-family A (ABC1), member 1
779	ADAMTSL3	ADAMTS-like 3
780	C10orf137	chromosome 10 open reading frame 137
781	CACNA2D3	calcium channel, voltage-dependent, alpha 2/delta subunit 3
782	CSMD1	CUB and Sushi multiple domains 1
783	DNAH1	dynein, axonemal, heavy chain 1
784	ERCC6L	excision repair cross-complementing rodent repair deficiency, complementation group 6-like
785	EVC2	Ellis van Creveld syndrome 2
786	FBN2	fibrillin 2
787	FNDC1	fibronectin type III domain containing 1
788	GUCY1A2	guanylate cyclase 1, soluble, alpha 2
789	HUWE1	HECT, UBA and WWE domain containing 1
790	UNC79	KIAA1409
791	LAMA1	laminin, alpha 1
792	NAV3	neuron navigator 3
793	OR51E1	olfactory receptor, family 51, subfamily E, member 1
794	PLCG2	phospholipase C, gamma 2 (phosphatidylinositol-specific)
795	SDK1	sidekick homolog 1, cell adhesion molecule (chicken)
796	STAB1	stabilin 1
797	TBX22	T-box 22
798	TGM3	transglutaminase 3 (E polypeptide, protein-glutamine-gamma-glutamyltransferase)
799	TNN	tenascin N
